# NASA’s ECOSTRESS satellite reveals widespread midday depression in ecosystem evapotranspiration

**DOI:** 10.1038/s41467-026-76873-x

**Published:** 2026-08-25

**Authors:** Jingyi Bu, Jingfeng Xiao, Joshua B. Fisher, Yiqi Luo

**Affiliations:** 1 Earth Systems Research Center, Institute for the Study of Earth, Oceans, and Space, University of New Hampshire, Durham, NH USA; 2 Schmid College of Science and Technology, Chapman University, Orange, CA USA; 3 Hydrosat, Inc., Washington, DC USA; 4 Soil and Crop Sciences Section, School of Integrative Plant Science, Cornell University, Ithaca, NY USA

**Keywords:** Ecology, Carbon cycle, Ecosystem ecology, Climate-change ecology

## Abstract

Plants often exhibit a midday depression in water use (i.e., transpiration), reflecting a constraint on their ability to sustain maximum water transport, which may occur at the cost of reduced photosynthesis. Eddy covariance observations and geostationary satellites cannot quantify this widespread phenomenon globally while resolving fine-scale spatial variability. Using evapotranspiration measurements from the ECOsystem Spaceborne Thermal Radiometer Experiment on Space Station (ECOSTRESS) and machine learning, we quantify the global distribution of midday depression in evapotranspiration. Midday depression primarily occurs during peak-growing seasons in temperate zones and dry periods in the tropics, with a morning shift of the peak evapotranspiration time by > 0.5 h. Droughts and heatwaves intensify midday depression, with peak evapotranspiration time shifting earlier, typically by 0.5 to 1.5 h and approaching 2 h during local extremes. By contrast, the coarse resolution upscaled flux products and hourly meteorological reanalysis datasets cannot capture this phenomenon. Midday depression of evapotranspiration detected using ECOSTRESS and machine learning is strongly associated with plant water stress, and helps us better understand how plants use water during drought and heatwaves.

## Abstract (web summary)

Plants often reduce water use around midday when heat and dry air increase water stress. Using ECOSTRESS satellite data and machine learning, the study shows that this midday depression of evapotranspiration is widespread, intensifies during droughts and heatwaves, and is not captured by coarser datasets.

## Introduction

Plants primarily lose water through transpiration via stomata in their leaves, which open to assimilate CO_2_ for photosynthesis and conserve water upon closure^1–3^. Within a single day, even slight variations in environmental factors could trigger immediate changes in stomatal behavior^4–6^. Under environmental stresses, such as high atmospheric demand and water scarcity, typically in the afternoon, plants close stomata to minimize water loss, which also limits photosynthesis, with additional influence from increasing internal leaf CO_2_ concentration^3–12^. This adaptation strategy serves to avoid xylem embolism, further affecting plants’ ability to transport water and nutrients^2,13–15^. The occurrence and degree of midday stomatal closure indicate plant water status and can provide insights into water use and stress levels within plants^16,17^.

Field experiments and observations have confirmed the existence of midday stomatal closure and its response to environmental factors^8,10,18–22^. However, it is not known how prevalent diurnal asymmetry in evapotranspiration (ET) is across plant functional types (PFTs) and climatic zones globally. Research on diurnal ET dynamics and environmental responses has previously relied on eddy covariance (EC) measurements^19,23–28^. However, EC flux sites have small footprints (e.g., <1 km in diameter) and are sparsely distributed, which challenges the scaling of instantaneous observations from the ecosystem level to the globe^16,29^.

Satellites enable observations of ecosystem functioning across regional or global scales. The study of global water and carbon cycles based on satellite observations has been concentrated on daily or longer time scales, primarily using data from polar-orbiting satellites and sensors such as Landsat^30^, the Advanced Very Higher Resolution Radiometer (AVHRR)^31^, and the Moderate Resolution Imaging Spectroradiometer (MODIS)^32,33^. With a polar orbit, each of these satellites or sensors provides only one or two snapshots of a given location at fixed times of day and does not have diurnal sampling capability^16,34,35^. Geostationary satellites can provide continuous observations throughout the day and night and have been used to investigate diurnal ET^36–38^, but individual geostationary satellites lack the global coverage and fine spatial resolution needed to capture global or field-scale surface variations^16^.

Since its inception in June 2018, the ECOsystem Spaceborne Thermal Radiometer Experiment on Space Station (ECOSTRESS) has been revolutionizing Earth observation of plant water stress and water use globally, between 52° North and 52° South^17^. Remarkably, with the precessing orbit of the International Space Station, ECOSTRESS provides land surface temperature (LST) and ET data for different times of day. ECOSTRESS also achieves an exceptional resolution of 70 m, allowing for finer detail and greater accuracy in ET observations than ever before, particularly useful for disentangling different water use strategies throughout landscapes. Additionally, the 1–5-day revisit cycle ensures frequent and up-to-date data collection^39^ and is pivotal in understanding and monitoring environmental changes, water resource management, and agricultural practices^29,40–46^. However, accurate quantification of diurnal ET dynamics is hindered by the sparse and discontinuous sampling of ECOSTRESS, and requires lumping all the snapshots over multiple weeks or months. With plant growth and rapid environmental changes, the variability in ET among these snapshots can include both diurnal and day-to-day variability^16^. While the ECOSTRESS mission covers a broad portion of Earth’s land surface, earlier investigations based on ECOSTRESS ET data have also been limited to certain specific regions^16,29,41,42,44,45^.

Here we generate a continuous global hourly ET dataset based on ECOSTRESS observations and examine the diurnal asymmetry of global land ET. We first develop a gridded, hourly ET dataset from 2018 to 2022 by training a machine learning (Random Forest) model on ECOSTRESS ET snapshots using continuous ancillary data as predictors. The gridded, hourly ET dataset, referred to as ECHO-ET (i.e., ECOSTRESS-based Hourly ET) for simplicity hereafter, is then used to examine how ET varies over the course of the diurnal cycle for every location across the globe and how the diurnal pattern changes throughout the year. This work monitors the diurnal asymmetry (e.g., midday depression) of land ET on a global scale. Our study provides an extensive analysis of convergence and variability of diurnal pattern in ET among PFTs and across diverse climatic zones. The analysis of the responses of ET to drought and heatwaves over the course of the day can provide valuable insights into the impacts of climate change on diverse ecosystems and their resilience to environmental stresses. Understanding the diurnal patterns of ET can also help improve land surface models that predict land ET and surface energy exchange.

## Results

### Patterns of diurnal shifts in global ET

**Fig. 1:**
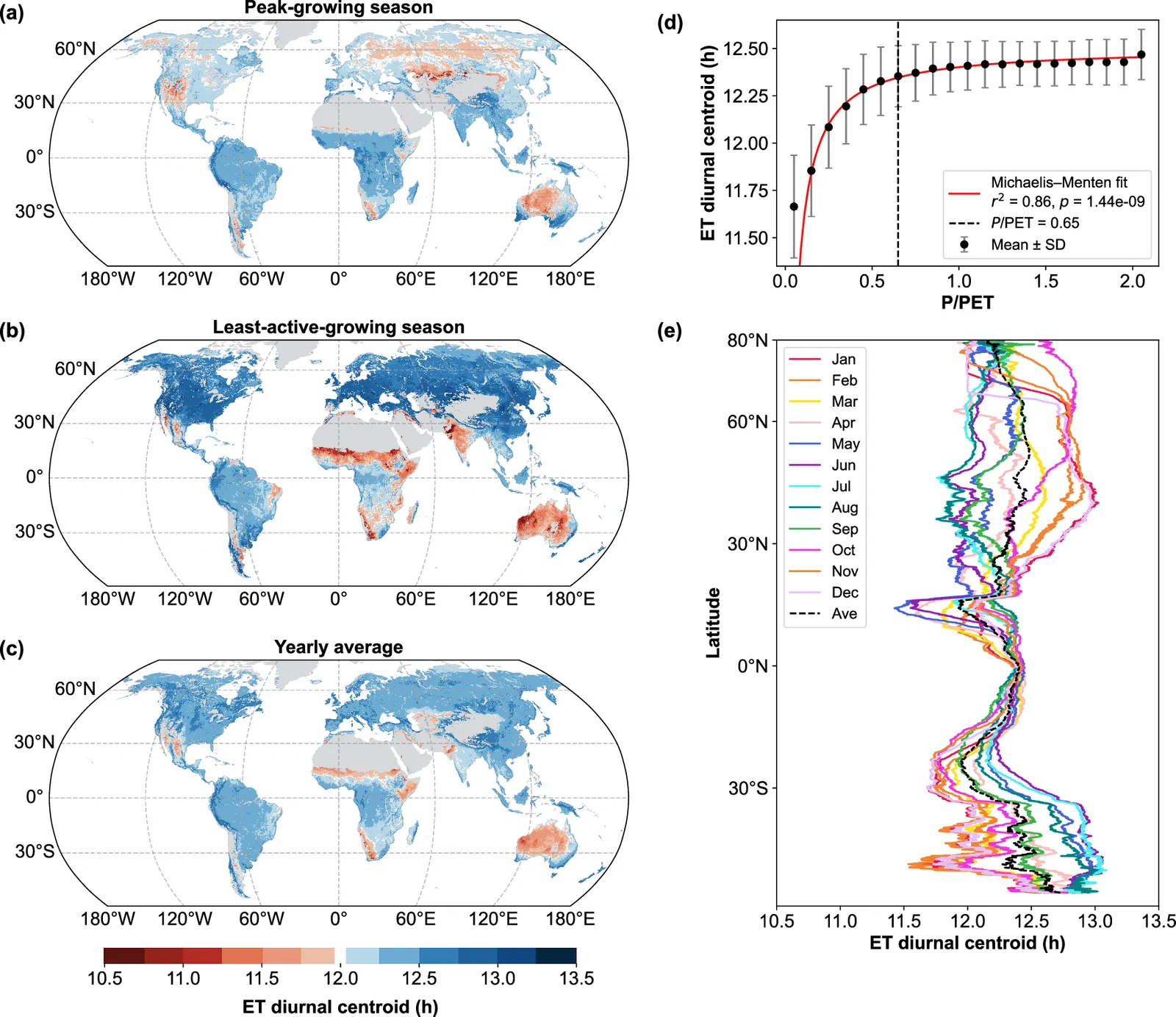
Variations in global diurnal centroid of evapotranspiration (ET) (C_ET_: time of peak ET) based on our ECHO-ET (i.e., ECOSTRESS-based Hourly ET) dataset. Widespread morning shift of C_ET_ was observed in different growing seasons of vegetation, and it was more pronounced in drylands. **a** C_ET_ in peak-growing season. **b** C_ET_ in least-active-growing season. **c** Yearly average of C_ET_. See Fig. S1 for monthly C_ET_ over a year averaged from 2018 to 2022. The difference in C_ET_ between the peak-growing and least-active-growing seasons, along with the standard deviation of monthly C_ET_, is presented in Fig. S4. **d** The yearly average of C_ET_ as a function of P/PET, the ratio of annual precipitation (P) to potential evapotranspiration (PET). The P/PET was divided into intervals of 0.1, and then the value of yearly average C_ET_ was calculated for each interval. The C_ET_ values were classified into one group if P/PET was greater than 2. Data points (sample size = 21) represent mean C_ET_ values within each P/PET bin, and error bars indicate ±1 standard deviation. **e** The latitudinal profile of monthly C_ET_.

We calculated the mean diurnal centroid of hourly ET (C_ET_) globally from ECHO-ET during 2018–2022 as a metric of diurnal variation. The diurnal centroid can describe the central point of a variable (i.e., ET in this study) over the course of a day, indicating whether the peak ET predominantly occurs around noon or is shifted towards either the morning or afternoon^24^. The spatial patterns of C_ET_ from the gridded ET (Fig. S1) closely resembled those from 194 EC flux sites around the world (mean difference of 9 ± 28 minutes and median difference of 7 ± 28 min), particularly in drylands (Fig. S2). C_ET_ generally varied globally with PFT, though significant variations in the distribution of the C_ET_ were evident in different growing stages (Figs. 1a–c, S3). During the peak-growing seasons in extratropical regions, a morning shift of C_ET_ (before local solar noon) was found (Fig. 1a), and C_ET_ of the least-active-growing season lagged that of the peak-growing season by two hours (Fig. 1a, b). In Mediterranean regions, the morning shift in ET occurred during the dry summer months, coinciding with the least-active-growing season (Fig. 1b and Fig. S3). In the tropics, there was a distinct morning shift from January to April and from August to October, relative to the rest of the year (Fig. S1). The yearly average C_ET_ varied in magnitude with water limitations, as evidenced by the Michaelis-Menten fitting model (*r*^2^ = 0.86, *p* = 1.44 × 10^−9^) (Fig. 1d). In arid and semi-arid areas, C_ET_ generally exhibited a positive correlation with P/PET, the ratio of annual precipitation (*P*) to potential ET (PET). However, as P/PET surpassed a certain threshold of 0.65 mm mm^−1^ (transitioning from semi-arid to humid regions), C_ET_ tended to stabilize at 12.45 h local solar time. Time is expressed as decimal hours (e.g., 12.45 h = 12:27 local solar time).

The latitudinal profile of C_ET_ exhibited temporal variation throughout the year, with considerable differences observed across latitudes (Fig. 1e). In the Northern Hemisphere (above 0 degrees), the C_ET_ advanced by about one hour around the middle of the year (i.e., the Northern Hemisphere’s summer). Similarly, in the temperate regions of the Southern Hemisphere, approximately south of 23.5°S, a similar shift occurred in the beginning and the end of the year (i.e., the Southern Hemisphere’s summer). While in tropical regions of the Southern Hemisphere (from 0 to 23.5°S), there were almost no fluctuations in the mean C_ET_ throughout the year. Overall, the results from the latitudinal profile of the C_ET_ were consistent with the spatial pattern presented in Fig. 1a, b.

### Contrasting diurnal patterns between ET and potential ET

**Fig. 2:**
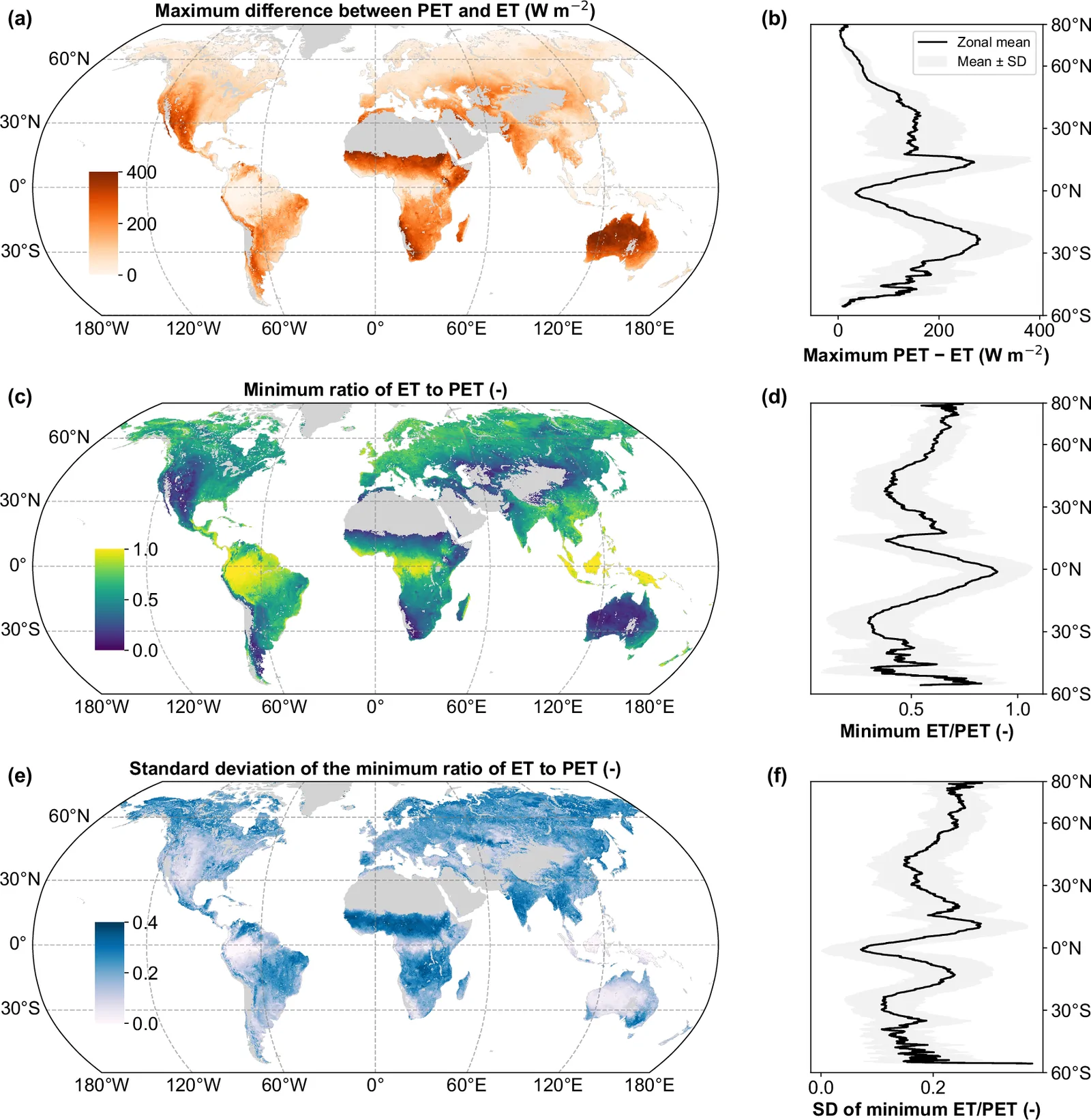
Global patterns of discrepancies between hourly evapotranspiration (ET) and hourly potential ET (PET). The discrepancies between ET and PET were more pronounced in drylands, which also coincided with the patterns of midday depression occurrences (Fig. S7). **a** Maximum difference between hourly PET and ET. **b** The latitudinal profile of maximum hourly PET-ET. **c** Minimum hourly ET/PET ratio. **d** The latitudinal profile of minimum hourly ET/PET. **e** Standard deviation of the minimum hourly ET/PET. **f** The latitudinal profile of the standard deviation of minimum hourly ET/PET. In (**b**), (**d**), and (**f**), black lines represent zonal mean values, and gray shaded bands indicate ±1 standard deviation across pixels within each latitude band.

Next, we quantified the spatial and temporal variability of the disparity between ET and PET as an indicator of water stress intensity. PET characterizes the maximum ET that can be reached with adequate water supply. The difference between PET and ET (i.e., PET-ET) and the ratio of ET to PET (i.e., ET/PET) are both used to quantify the magnitude of ET suppression, as ET will deviate further from PET throughout the day when there is more suppression. The maximum PET-ET, the minimum ET/PET, and the standard deviation of ET/PET on a monthly-average hourly basis are illustrated in Fig. 2. The regions with higher PET-ET values (Fig. 2a) and lower ET/PET values (Fig. 2c) were concentrated in arid areas, such as the North American drylands, the Sahel region in Africa, the Middle East, and the Australian Outback. The lowest PET-ET values were observed near the equator, the regions covered by dense rainforest. The ET/PET ratio demonstrated a lower degree of seasonal variation in the North American drylands and the Australian Outback, as well as around the equator, as indicated by the standard deviation (Fig. 2e). This suggests that the former regularly face conditions of water scarcity, while in the latter, actual ET remains relatively close to PET throughout the year.

To illustrate the discrepancies in the temporal fluctuations between peak ET and peak PET, we analyzed the difference between the diurnal centroid of ET (i.e., C_ET_) and that of PET (C_PET_) (C_PET_–C_ET_). The C_PET_ occurred in most regions and times of the year from 13.00 to 14.00 h local solar time (Fig. S5). However, C_P__ET_–C_ET_ showed considerable spatial and temporal variability in most regions, with C_ET_ preceding C_PET_ (Fig. S6). Specifically, in the temperate zone of the Northern Hemisphere, Cp_ET_–C_ET_ had a value greater than 0.5 h during the peak-growing season, which is also usually summer, whereas in the tropics and in the Southern Hemisphere, the large disparity mostly occurred in the least-active-growing period, which is usually the dry season.

These differences between ET and PET were generally consistent with the  the spatial and temporal patterns of the midday depression in ET (Fig. 1), particularly in drylands (Fig. S7). In these regions, even if energy is sufficient, it cannot be fully utilized for ET due to limited water supply^47–49^.

### Water and energy limitation on midday ET

**Fig. 3:**
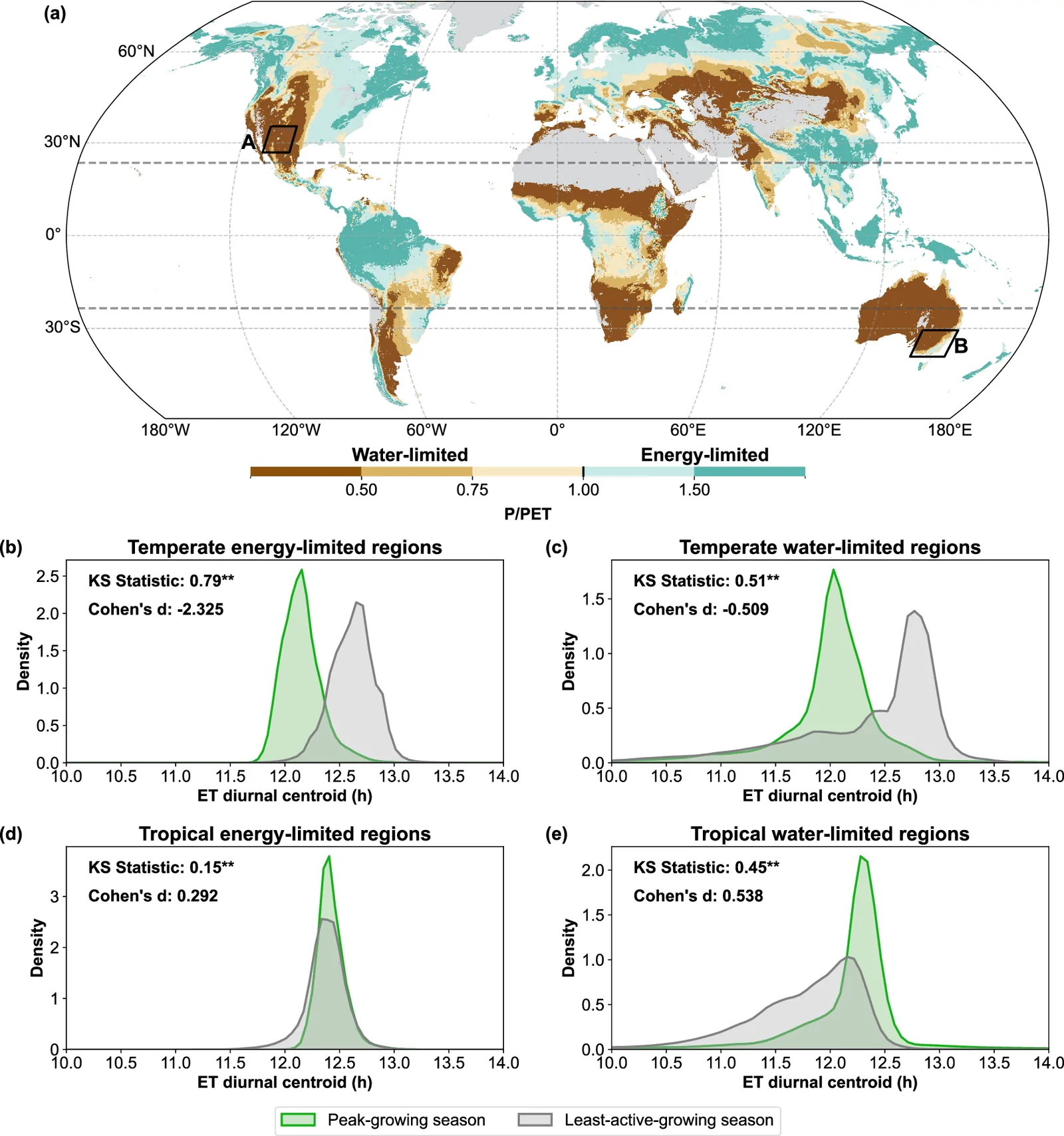
Convergence and divergence in diurnal variations in evapotranspiration (ET) across environmental conditions. Midday depression generally occurs during the peak-growing season in temperate regions, while in the tropics, it coincides with times of water shortage. Two-sided two-sample Kolmogorov-Smirnov (KS) statistic test and Cohen’s d effect size value were employed to assess the similarity between two distributions of ET diurnal centroid (C_ET_). The asterisk (**) denotes that the result is statistically significant at the 0.001 significance level. All comparisons marked with ** had *p* values < 1 × 10⁻³⁰⁰. **a** Global distribution of energy-limited and water-limited regions following McVicar et al.^50^. The ratio of mean annual precipitation (P) to mean annual potential ET (PET), P/PET, of 2018–2022 is shown, where regions with ratios >1.0 are referred to as energy limited and regions with ratios <1.0 are referred to as water limited. The distribution of the C_ET_ in (**b**) energy-limited regions in temperate zones, **c** water-limited regions in temperate zones, **d** energy-limited regions in tropical zones, and **e** water-limited regions in tropical zones. For the distribution analyses, sample size represents the number of valid land pixels included in each comparison. The peak-growing and least-active-growing sample sizes were 2,068,140 and 2,067,809 pixels for temperate energy-limited regions, 1,989,844 and 1,997,286 pixels for temperate water-limited regions, 578,941 and 578,909 pixels for tropical energy-limited regions, and 1,041,699 and 1,047,860 pixels for tropical water-limited regions, respectively.

To explore the zonal characteristics of midday depression and how midday depression varies with environmental conditions, we investigated the characteristics of ET diurnal patterns in regions with water and energy constraints in temperate and tropical zones (Fig. 3a). In the temperate zone, the distribution patterns of C_ET_ in the areas limited by energy (Fig. 3b) and water (Fig. 3c) were essentially similar. The two distributions of C_ET_ over the peak growing season and least-active-growing season yielded Kolmogorov-Smirnov (KS) statistics of 0.79 (energy-limited) and 0.51 (water-limited), suggesting a significant difference between the distributions for both energy-limited and water-limited regions (two-sided two-sample KS test; energy-limited: KS statistic = 0.79, *p* < 1 × 10⁻³⁰⁰; water-limited: KS statistic = 0.51, *p* < 1 × 10⁻³⁰⁰). The Cohen’s d with value of –2.325 (energy-limited) and –0.509 (water-limited) indicates a large negative effect size, suggesting that the C_ET_ of the peak-growing season substantially preceded that of the least-active-growing season. During the peak-growing season, key physiological processes such as carbon assimilation and stomatal regulation could reach their peak earlier in the day to avoid environmental stress, while during the least-active-growing season, when heat is often the primary limiting factor in temperate zones, peak activity may occur later in the day when temperatures are more moderate.

However, for the tropical zone, the distribution patterns of areas limited by energy (Fig. 3d) and water (Fig. 3e) exhibited distinct characteristics. Only a small disparity was observed in energy-limited regions, with KS statistic of 0.15 and Cohen’s d of 0.292, suggesting that the diurnal patterns of vegetation activity tended to be more consistent throughout the year (two-sided two-sample KS test; KS statistic = 0.15, *p* < 1 × 10⁻³⁰⁰). By contrast, in the water-limited regions, the KS statistic of 0.45 and Cohen’s d of 0.538 indicated significant differences between the two distributions for least-active-growing and peak-growing season periods (two-sided two-sample KS test; KS statistic = 0.45, *p* < 1 × 10⁻³⁰⁰). Despite a peak in the distribution of C_ET_ for peak-growing season around 12.00–12.50 h local solar time, the distribution for the least-active-growing season appeared flatter and extended to earlier hours. This suggests that the morning shift was more pronounced in the least-active-growing season, and vegetation in these areas typically displayed a pronounced response to water availability^48,50,51^.

Based on the above findings, we also investigated the convergence and differences among PFTs (Fig. S8). In the peak-growing season, the C_ET_ distribution and averages tended to advance compared to those of the least-active-growing season, except for Evergreen Broadleaf Forests (EBF), Closed Shrublands (CSH), and Open Shrublands (OSH). Among these, EBF exhibited the smallest differences in mean and median C_ET_ between the two periods. This muted seasonal variability in EBF likely reflects the relatively stable environmental conditions and canopy structure characteristic of tropical evergreen forests, including limited seasonal variation in water availability, temperature, and effective canopy area. The complexity of shrublands may be correlated with their widespread distribution, as they are found in tropical, high-latitude, and high-altitude regions.

### Impacts of drought and heatwaves on diurnal cycling of ET

**Fig. 4:**
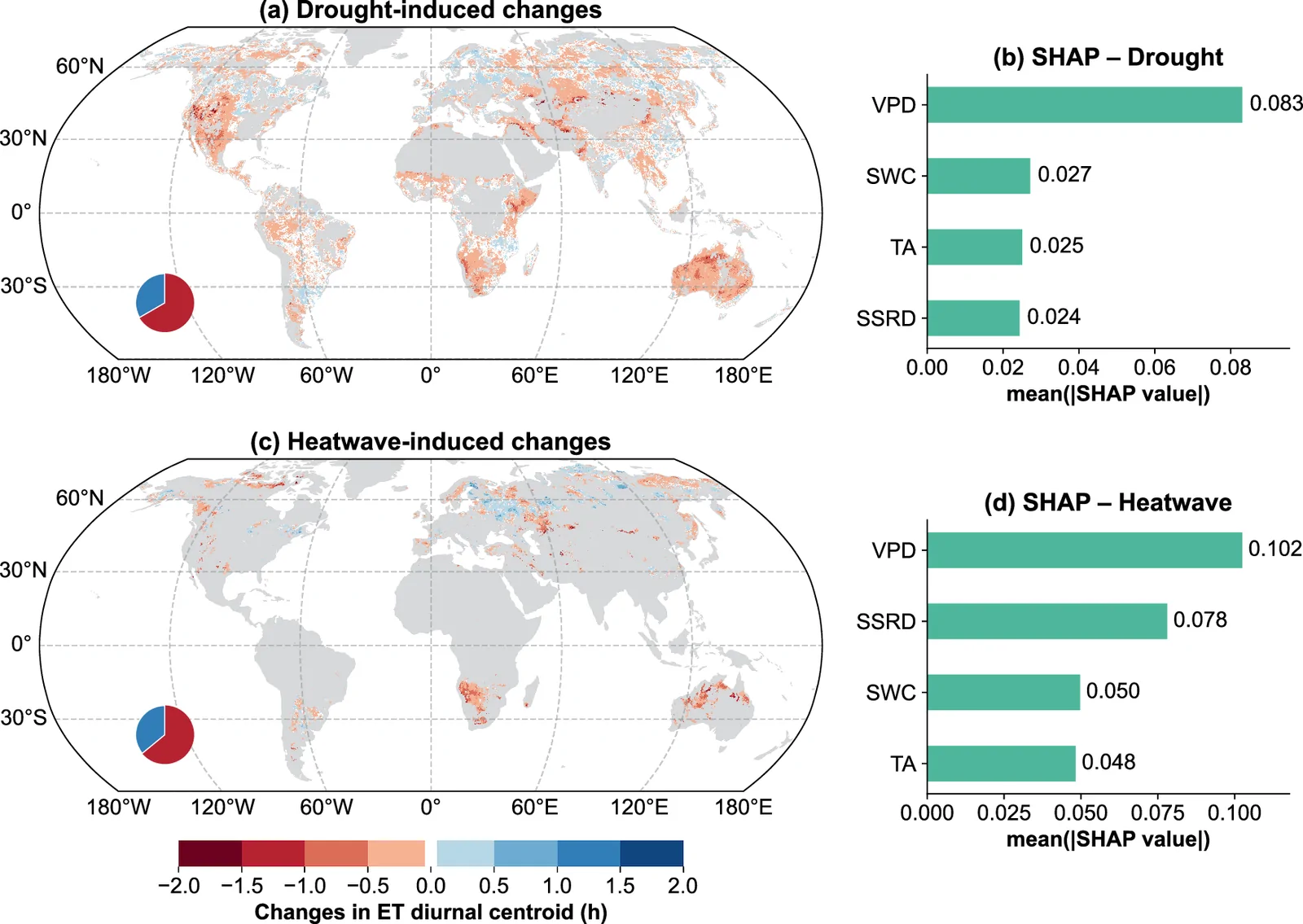
Global patterns and drivers of drought- and heatwave-induced shifts in diurnal centroid of evapotranspiration (ET) (C_ET_) during the peak-growing season from 2018 to 2022. **a** Global spatial distribution of drought-induced changes in the C_ET_. C_ET_ anomalies were calculated as the difference in C_ET_ between drought conditions and the corresponding near-normal baseline at the same location. Negative values (red) indicate earlier C_ET_ (advanced ET peak), whereas positive values (blue) indicate delayed timing. **b** Relative importance of environmental drivers explaining drought-induced C_ET_ anomalies, quantified using Shapley Additive Explanations (SHAP) values. Bars represent the mean absolute SHAP value (mean |SHAP | ) for vapor pressure deficit (VPD), soil water content (SWC), air temperature (TA), and downward shortwave radiation (SSRD), indicating each variable’s overall contribution to model predictions. **c** Global spatial distribution of heatwave-induced changes in C_ET_. As in (**a**), negative values denote earlier C_ET_ and positive values denote delayed C_ET_. **d** SHAP-based attribution of heatwave-induced C_ET_ anomalies. Pie charts in (**a**) and (**c**) summarize the global proportion of pixels exhibiting earlier (red) versus delayed (blue) C_ET_ shifts.

We next examined how extreme climate conditions alter the diurnal dynamics of ET during the peak-growing season. Specifically, we quantified shifts in the C_ET_ under drought and heatwave events relative to near-normal conditions. Under drought conditions, widespread shifts in the C_ET_ were observed across major vegetated regions globally (Fig. 4a). Negative anomalies, indicating earlier timing of peak ET, dominated large areas of western North America, southern Europe, East Asia, southern Africa, and Australia. The magnitude of drought-induced shifts commonly ranged from –0.5 to –1.5 h, with localized extremes approaching –2 h. Positive anomalies were comparatively sparse and mainly confined to limited high-latitude or humid regions. Overall, the spatial pattern revealed a broad and coherent advancement of the C_ET_ during drought events, with the majority of affected grid cells exhibiting a morning shift. In contrast, heatwave-induced shifts displayed a more spatially fragmented and regionally concentrated pattern (Fig. 4c). Significant changes were primarily detected in Australia, southern Africa, parts of South America, and mid-latitude Eurasia. Similar to drought conditions, most affected regions exhibited negative anomalies, reflecting an earlier occurrence of peak ET during prolonged high-temperature events. However, compared to drought, the spatial extent of heatwave-induced shifts was smaller and more heterogeneous, although locally the magnitude of advancement was comparable, occasionally reaching -2 h. Taken together, both drought and heatwave events were associated with systematic advancements in the C_ET_ during the peak-growing season. Drought impacts appeared more spatially extensive, whereas heatwave responses were more regionally concentrated but could exhibit comparable intensity at local scales.

To further disentangle the relative contributions of meteorological drivers to these event-induced shifts, we applied a SHapley Additive exPlanations (SHAP)-based attribution analysis. By decomposing model predictions into additive feature contributions, we quantified the importance of vapor pressure deficit (VPD), soil water content (SWC), air temperature (TA), and downward shortwave radiation (SSRD) in explaining variations in C_ET_ anomalies. Under drought conditions, VPD emerged as the dominant driver, with high VPD values consistently associated with negative SHAP values (Fig. S9), indicating a systematic shift toward earlier C_ET_ (Fig. 4b). SWC exhibited a secondary influence, further highlighting water limitation as the primary control mechanism, whereas TA and SSRD played comparatively weaker roles. During heatwave events, high VPD consistently showed negative SHAP values, indicating a systematic shift toward earlier C_ET_ (Fig. 4d). Notably, the influence of SSRD became substantially stronger, suggesting that enhanced radiative forcing played a critical role alongside atmospheric dryness. These patterns indicate that drought responses are primarily water-limited, whereas heatwave responses involve combined effects of atmospheric demand and radiative stress.

**Fig. 5:**
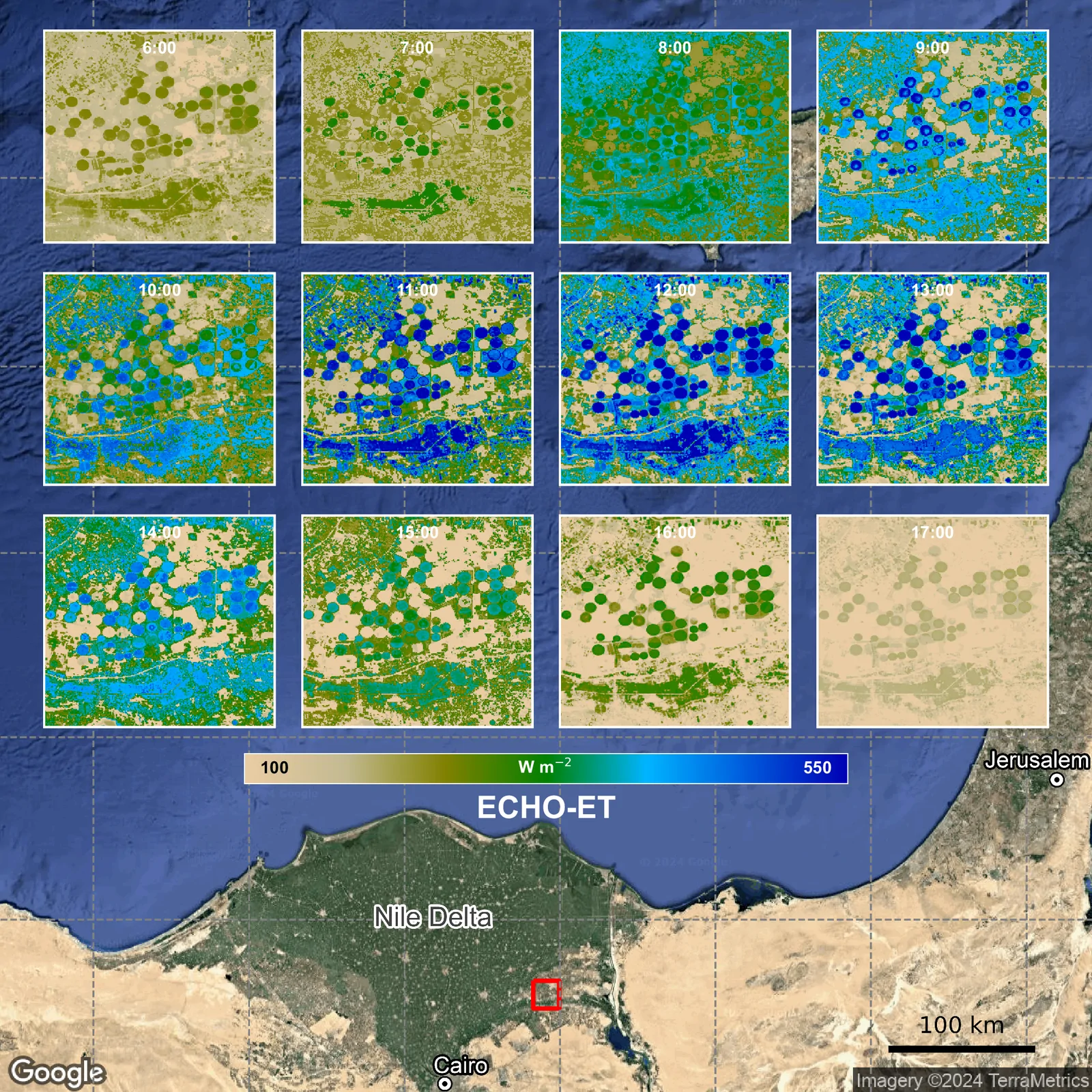
Diurnal cycle of evapotranspiration (ET) over agricultural fields (center pivot irrigation) in the Nile Delta, Egypt, on August 24, 2018. One irrigation event occurred on that day. The center of the images is located at 30.54° N and 31.85° E. The inset maps show ET values at hourly intervals from 6:00 to 17:00 local time, with the study area highlighted by a red box in the background map. The inset maps are shown at 70 m resolution using the ECHO-ET framework, with the global product at 0.05˚ resolution. This figure highlights the water use dynamics within a single day in a predominantly agricultural region of the Nile Delta, demonstrating clear temporal and spatial variability. The background map was obtained from Google Static Maps using the GoogleVisibleMap function in the Salem Python package (version 0.3.11).

The ECHO-ET framework (0.05˚ globally), when applied at 70 m resolution for specific regions, can enable analysis of diurnal patterns of ET at the field scale, especially in understanding the impact of extreme climate events on the diurnal ET dynamics. For example, cropland ET varied substantially over the course of the day in the Nile Delta; near midday, some crop fields exhibited high ET, whereas others showed much lower ET (Fig. 5). This pattern suggests that certain crop fields were experiencing water stress and may require irrigation.

**Fig. 6:**
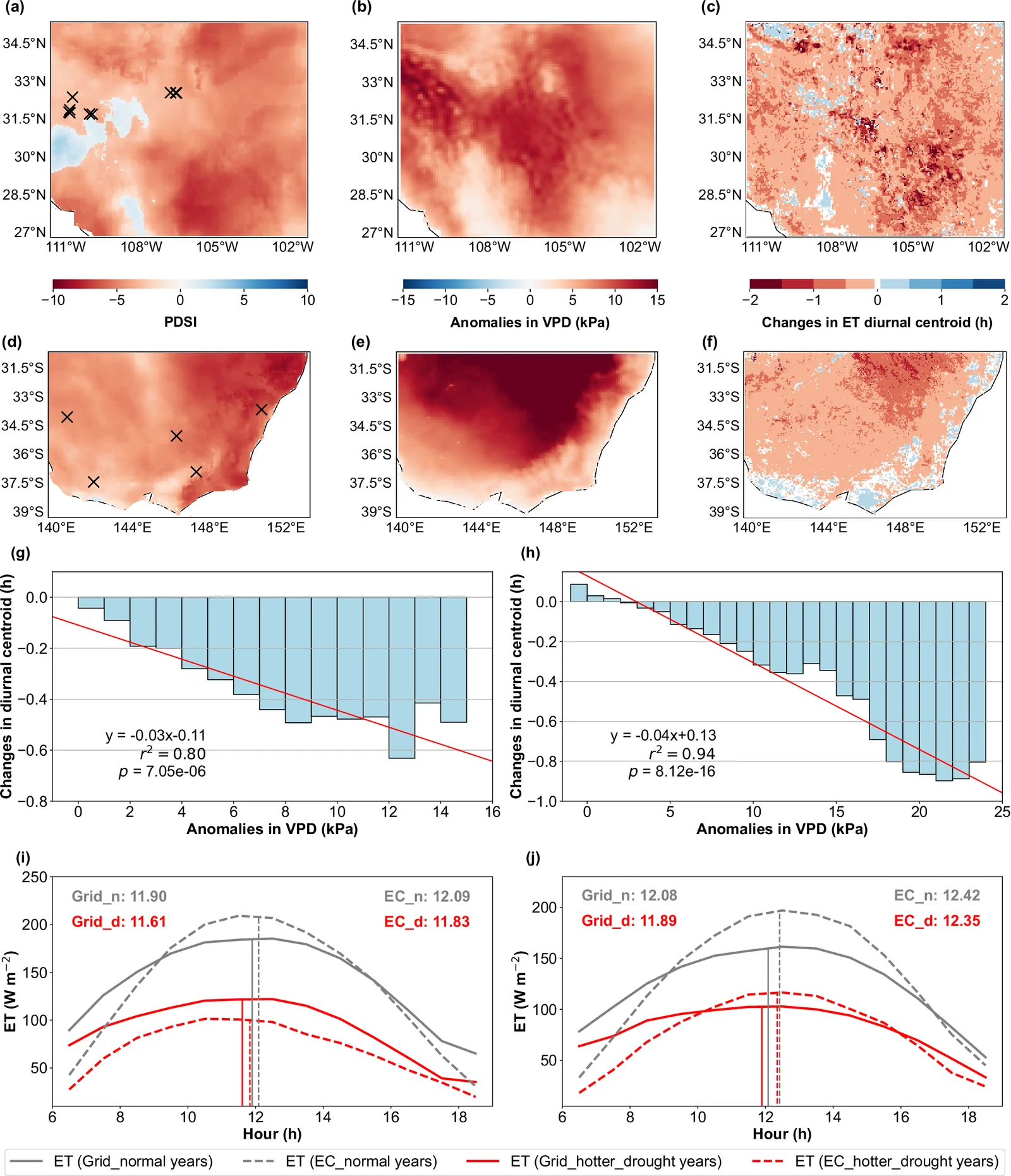
Effects of compound drought and heatwave events on the diurnal dynamics of evapotranspiration (ET). Effects of drought and heatwave compound events on diurnal variations of ET in (**a**–**c**, **g**, **i**) southwestern North America during August 2020 and (**d**–**f**, **h**, **j**) southern Australia during December 2019. Droughts and heatwaves exacerbated the diurnal asymmetry of ET. **a**, **d** the Palmer Drought Severity Index (PDSI) map and the locations of eddy covariance (EC) towers (black crosses). The locations of the two study domains are shown in Fig. 3a (A: southwestern North America, B: southern Australia). The PDSI values during the normal years are also illustrated in Fig. S10. The EC towers in southwestern North America include US-MtB, US-xSR, US-SRG, US-SRM, US-Whs, US-Wkg, US-xJR, US-Jo1, and US-Jo2. In southern Australia, the EC towers include AU-Ync, AU-APL, AU-Cum, AU-Cpr, and Gatum Pasture. More details on the EC towers are given in Supplementary Data 1. **b**, **e** The vapor pressure deficit (VPD) anomaly map, represented by the difference between the VPD in a drought/heatwave year and the average VPD in normal years. **c**, **f** Difference in diurnal centroid in ET (C_ET_) between drought/heatwave and normal years, former minus later. **g**, **h** Average changes in C_ET_ response to VPD intervals. We divided the VPD into intervals of 1 kPa and then calculated the average value of changes in C_ET_ for each interval (southwestern North America: sample size = 15; southern Australia: sample size = 25). The changes in C_ET_ and anomalies in VPD showed a strong negative correlation, indicating that greater anomalies in VPD were associated with a more pronounced morning shift in C_ET_. **i**, **j** Time series of average gridded ET (solid lines) in the whole domain and the ET observed by the EC technique (dotted lines). The suffixes _d and _n represent drought/heatwave and normal years, respectively. The vertical lines and numbers represent the C_ET_ in each case.

Two compound drought and heatwave events that occurred in southern Australia during December 2019^16^ and southwestern North America during August 2020^34,52^ were studied in this research (Fig. 6). Both study regions are primarily water-limited ecosystems dominated by herbaceous plants and shrubs. The severity and extent of the heatwaves in these regions were characterized using air temperature and VPD anomalies (Figs. 6b, e, and S10). The drought extent and severity of the two regions were revealed by the Palmer Drought Severity Index (PDSI) (Fig. 6a, d and S10). The anomaly maps of C_ET_ (Fig. 6c, f) indicated a widespread midday depression in ET during these drought and heatwave compound events. The maps of PDSI, VPD anomalies, and C_ET_ anomalies showed similar spatial patterns. The shift in C_ET_ exhibited a significant correlation with VPD anomalies and PDSI across regions, showing that regions that experienced more severe heatwaves/drought had an earlier morning shift of C_ET_ (Fig. 6g, h, and S10).

We also compared the temporal variations in the hourly ET averaged over all grid cells between drought and normal years, which did not experience heatwaves or droughts, and the variations in the observed ET between drought and normal years (Fig. 6i, j). Similar to the anomaly maps, the C_ET_ of the average time series for ECHO-ET and EC observations was shifted by –0.26 h and –0.29 h, respectively, over southwestern North America (Fig. 6i), and by –0.19 h and –0.07 h, respectively, over southern Australia (Fig. 6j). Both the ECHO-ET dataset and the EC observations showed that drought reduced ET but increased ET asymmetry throughout the day.

**Fig. 7:**
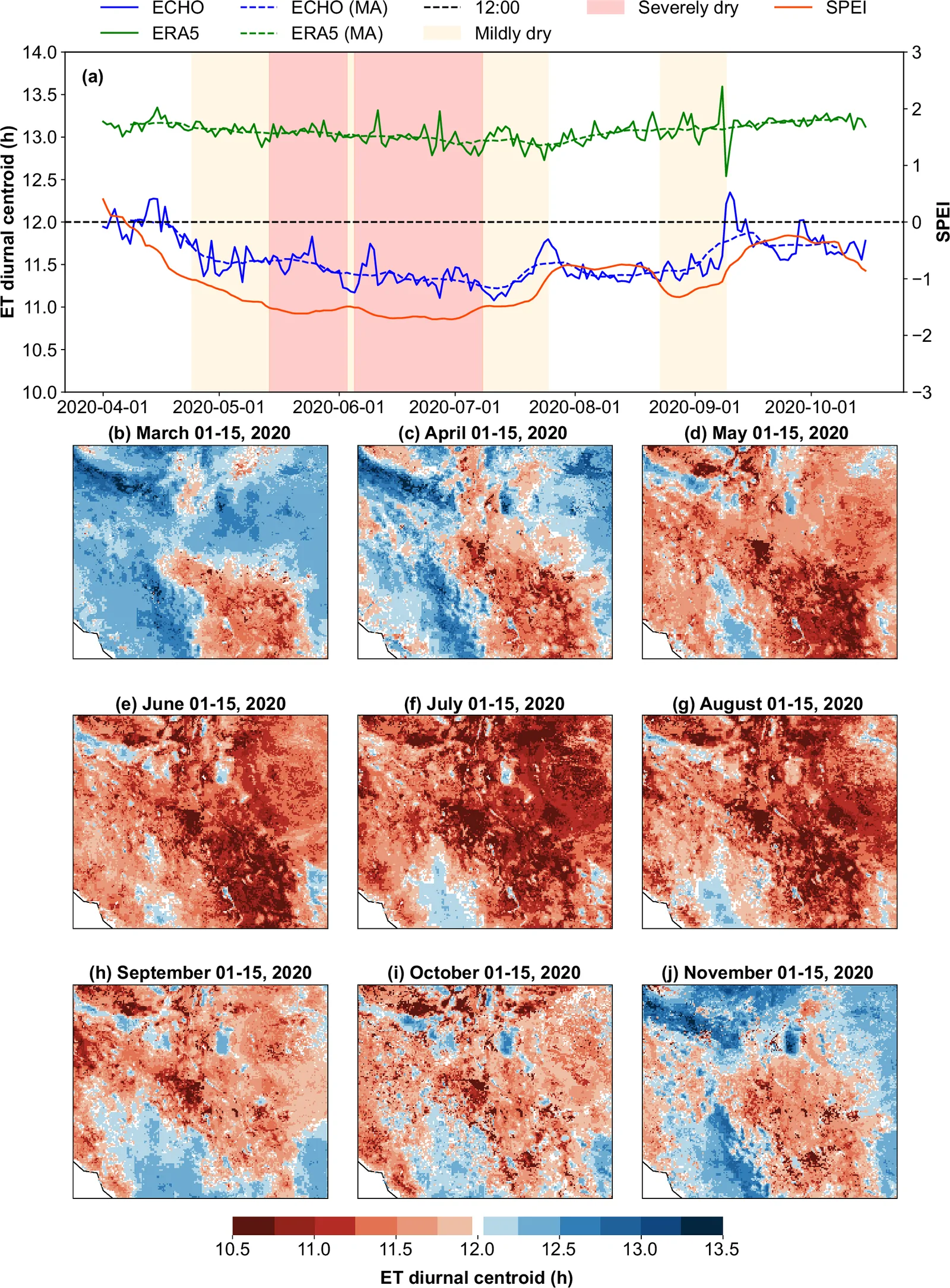
Evolution of daily diurnal centroid in evapotranspiration (ET) (C_ET_) dynamics and standardized precipitation-evapotranspiration index (SPEI) 53 trends in southwestern North America during drought and heatwave compound events in 2020. **a** Time series of daily average C_ET_ from ECHO-ET (i.e., ECOSTRESS-based Hourly ET) and ERA5-Land, along with SPEI values, from April to October 2020, illustrating the relationship between morning shifts in C_ET_ and drought severity (indicated by SPEI and shaded regions for “mildly dry” and “severely dry” periods). The dashed curves indicate the 5-day moving average (MA). **b**–**j** Spatial distribution of C_ET_ across the study area during different 15-day periods from March (pre-drought) to November (post-drought) in 2020.

We further examined the relationship between daily C_ET_ and standardized precipitation-evapotranspiration index (SPEI)^53^ to explore how the asymmetry in diurnal cycling of ET evolves throughout the processes of drought development and recovery (Fig. 7). SPEI demonstrated a declining trend in the early months in southwestern North America, with a sharp drop in June and July indicating severe dry conditions, and then began to recover in September (Fig. 7a). The average C_ET_ showed significant fluctuations, generally ranging between 11.00 and 12.50 h local solar time (Fig. 7a). Consistent with the onset of drought, C_ET_ began to exhibit a morning shift trend starting in April. As the drought progressed, its intensity peaked in June and July. Following this peak, C_ET_ gradually recovered, eventually returning to its pre-drought state by late September. The spatial variation in diurnal centroids during different 15-day periods from March to November 2020 was strongly correlated with drought severity (Fig. 7b–j). A widespread and significant morning shift in the C_ET_ occurred in June and July, with most regions peaking around 10.50–11.50 h local solar time, aligning with the peak of drought intensity. By November, the spatial patterns closely resembled those of March, indicating a return to pre-drought conditions. The temporal and spatial evolution of C_ET_ throughout the drought period in southern Australia indicated similar patterns (Fig. S11). The morning shift peaked during the most severe drought conditions in December 2019 and January 2020 and gradually recovered to pre-drought states by late February 2020. This highlighted the sensitivity of C_ET_ as an indicator of environmental stress.

### Comparison with diurnal ET from global observation-driven and reanalysis datasets

**Fig. 8:**
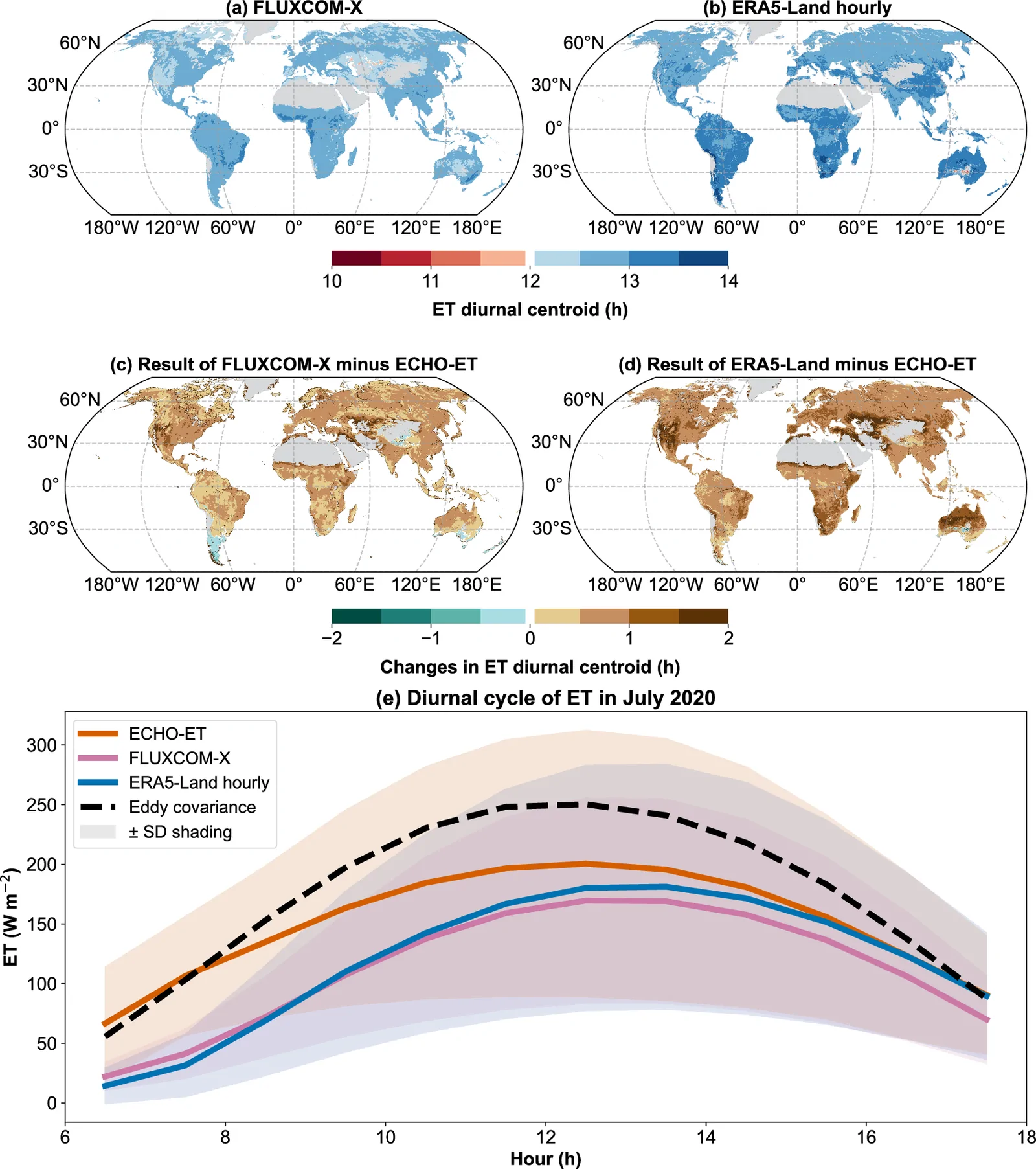
Diurnal variation of global hourly evapotranspiration (ET) in July 2020 derived from FLUXCOM-X and ERA5-Land. Compared to ECHO-ET (i.e., ECOSTRESS-based Hourly ET) and eddy covariance (EC) observations, FLUXCOM-X and ERA5-Land substantially overestimated ET in the afternoon. **a** Global diurnal centroid of ET (C_ET_) from FLUXCOM-X. **b** Global CET from ERA5-Land. **c** Difference in CET between FLUXCOM-X and ECHO-ET. **d** Difference in CET between ERA5-Land and ECHO-ET. **e** Diurnal cycling of ET from ECHO-ET, FLUXCOM-X, ERA5-Land, and EC. The shaded area represents ±1 standard deviation (SD).

We compared the diurnal cycling of ET based on the ECHO-ET dataset against that based on both an observation-driven product (FLUXCOM-X) and a global meteorological reanalysis dataset (ERA5-Land). Across vegetated regions, the C_ET_ of FLUXCOM-X occurred at approximately 12.50 h local solar time (Fig. 8a), whereas ERA5-Land exhibited a later centroid near 13.00 h local solar time (Fig. 8b). Spatial differences relative to ECHO-ET indicated that FLUXCOM-X showed broadly similar C_ET_, with a slight delay relative to ECHO-ET in many regions (Fig. 8c). ERA5-Land exhibited a more pronounced lag, averaging approximately one hour across humid and temperate regions and extending to nearly two hours in drylands (Fig. 8d). The disparities were equally pronounced across other months (Figs. S12–S16). Global spatially weighted mean diurnal ET in July 2020 from ECHO-ET, FLUXCOM-X, ERA5-Land, and EC observations was illustrated in Fig. 8e. Significant differences were observed between ECHO-ET and the two comparison datasets. ECHO-ET peaked around 12.00 h local solar time and showed symmetry between 6.00 and 18.00 h local solar time, where the afternoon ET (158 W m^-2^) roughly equaled the morning ET (142 W m^-2^), closely resembling the pattern of EC observations (r^2^ = 1, *p* = 2.16 × 10⁻¹⁴, sample size = 12). In contrast, both ERA5-Land and FLUXCOM-X exhibited clear afternoon dominance. ERA5-Land showed markedly higher ET in the afternoon (150 W m^-2^) than in the morning (89 W m^-2^), and FLUXCOM-X similarly presented higher afternoon ET (135 W m^-2^) relative to morning levels (90 W m^-2^). Compared with ECHO-ET, C_ET_ from ERA5-Land during drought was more stable, fluctuating near the 13.00 h local solar time in both southwestern North America (Fig. 7a) and southern Australia (Fig. S11a). There were only slight day-to-day variations, with no apparent response to the evolution of the drought.

## Discussion

Exploring the diurnal asymmetry of global ET can detect the environmental stress on ecosystems and global differences in plant water use^16,17^. ECOSTRESS can capture diurnal cycling of ET that traditional polar orbiting satellites are unable to detect^16,29,34,52^. In this study, the sparse and discontinuous ECOSTRESS ET snapshots were used to generate global gridded and hourly ET estimates (ECHO-ET) using a machine learning approach. Additionally, systematic errors in ECOSTRESS ET were corrected using global flux site observations. To achieve a global spatiotemporal continuum, we developed a 0.05° hourly product covering the entire globe from remote sensing. The 0.05° resolution struck a balance between spatial detail and global coverage, providing more detail of regional variability and localized patterns compared to the coarser scales of many reanalysis datasets and process-based model outputs^54,55^. Such spatial resolution may also result in the loss of finer spatial detail and introduce representativeness errors, particularly in heterogeneous landscapes. The ECHO-ET framework can also be applied to specific regions to generate 70-m hourly ET estimates (Fig. 5). The case study in the Nile Delta demonstrated that the 70 m hourly estimates can offer a highly detailed representation of surface features and are valuable for continuous ET monitoring of heterogeneous regions.

Our study quantified the diurnal variations of ET globally and unveiled that the diurnal asymmetry in ET is prevalent across the globe. Previously, the diurnal variations of ecosystem function were primarily investigated at the site level or within specific regions, such as temperate zone, often relying on ground-based observations or localized studies. A series of studies^19,24–28^ analyzed the underlying causes of this phenomenon and its response to environmental drivers through field observations. However, while valuable for understanding localized processes, these investigations were limited in spatial coverage and were unable to explain comparative variations across different climate regions, particularly in capturing the diurnal dynamics of ET under seasonal changes, weather extremes, and broader regional-to-global scales. Our approaches bridge the gap between localized studies and global assessments, particularly in regions with limited observations, such as large tropical and high-latitude areas. Our effort provides evidence for the widespread midday depression and offers an approach for heatwave or drought monitoring through the investigation of midday depression.

Understanding water and/or energy limitations in different climate zones is essential for interpreting the diurnal patterns of vegetation activity and the ecological implications. Previous site-scale studies have shown that diurnal variation of ET is primarily controlled by solar radiation, VPD, and soil water availability^1,26,27,56^. Our study reveals that seasonal variation of diurnal patterns is widespread across the globe and prevalent in various climate zones and vegetation types. Midday depression of ecosystem functioning typically occurs under conditions of reduced soil water content and elevated VPD^1,7,29,34^. During dry seasons or droughts/heatwaves, VPD can increase the humidity gradient between the air and plant canopy, resulting in a higher rate of transpiration^5,26^. This process assists plants in regulating their canopy temperature through evaporative cooling^57^. When soil moisture levels are low, increased transpiration can lead to plant water deficiency, which may cause water stress^4,58^. Water stress results initially in reduced turgor pressure and stomatal closure, eventually leading to wilting. Under prolonged or severe deficit, hydraulic failure occurs as xylem embolisms may form, potentially leading to the death of cells, organs, or entire organisms^59–61^. At sub-daily timescales, even moderate imbalances between atmospheric demand and plant water supply can transiently reduce turgor at the cell-to-leaf scale, constraining the ability of stomata to sustain maximum conductance. Although plants possess a range of physiological and biochemical mechanisms to modulate stomatal behavior and mitigate hydraulic risk, these active responses operate within shared biophysical limits on water transport. Consequently, midday depression in ET emerges as a largely passive and ubiquitous outcome of water loss exceeding supply, rather than solely a coordinated, signaling-driven response. ECHO-ET captures integrated canopy-scale ET rather than physiological responses at the cellular, organ, or individual-plant scales. Nevertheless, the globally coherent emergence of midday depression in ET suggests that passive hydraulic constraints associated with dehydration outweigh the variability introduced by fine-scale physiological signaling.

In the temperate zone, the climate exhibits substantial seasonal fluctuations. As a result, plants experience distinct periods of growth, and the peak growing period coincides with summer when heat is abundant. Vegetation activity is susceptible to environmental stresses during summer, particularly in drylands, and often peaks earlier in the day as increasing atmospheric demand later in the day constrains physiological processes, despite the role of transpiration in alleviating heat stress. In tropical water-limited regions, environmental stresses tend to occur during periods of water scarcity, leading to midday depression in the dry season^48,50,51^. By contrast, in tropical zones with limited energy resources, prolonged rainy seasons provide plants with sufficient water supply^62^. Some areas, such as rainforests, that are largely decoupled from atmospheric turbulent transfer^63^, high temperatures or high VPD during heatwaves are more likely to threaten ecosystem health^18,44^. However, the impact of high temperatures or VPD on ET is very complex^27,64,65^, and evaporation cooling may provide negative feedback that modulates the temperature rise^57^. The phenomenon of midday depression provides insight into the extent of water and heat stresses on plants.

Our study also suggests that the asymmetry of diurnal ET increases due to the suppression of ET during heatwaves and droughts. As the drought progresses and eventually alleviated, the diurnal ET asymmetry underwent notable changes, reflecting the dynamic response of diurnal ET patterns to varying drought conditions over time. This phenomenon highlights the sensitivity of plants (e.g., crops) to water availability and their adaptive response to changing environmental conditions. Therefore, when examined over time at the same location, midday depression can serve as an indicator of plant stress responses, providing early warning of impending drought and supporting agricultural management. This type of information is valuable for informing farmers and agricultural managers of crop water stress, enabling timely irrigation decisions. Current drought indicators such as PDSI and SPEI primarily capture meteorological or hydrological droughts, which may not accurately reflect the drought conditions that directly impact plant physiological activities^66,67^. In comparison, the role of midday depression is critical for both early warning and monitoring recovery from drought, as it serves as a direct reflection of the drought stress experienced by vegetation. This response can signal early stages of environmental stress before its effects are visible on a larger scale, allowing for timely intervention and mitigation strategies. Early detection of stress through midday depression can enhance the effectiveness of drought preparedness and response plans, thereby minimizing the impacts on agriculture and conservation efforts. However, differences in the magnitude or timing of midday depression across sites should be interpreted with caution, as they may also reflect variation in plant structure, community composition, or dominant physiological traits rather than stress alone.

The midday depression of ET, caused by stomatal closure, helps mitigate water loss and prevents plant damage from stress. However, this occurs at the cost of reduced photosynthesis despite high light availability^29,34,68^, decreasing plant growth efficiency and carbon uptake. Repeated or prolonged stomatal closure under stress can significantly diminish plant growth and ecosystem carbon sequestration^34,52^. Diurnal asymmetry in ET can also affect regional energy balance and cloud formation. Midday depression of ET reduces latent heat flux and enhances sensible heating, leading to a drier and deeper atmospheric boundary layer. This feedback tends to amplify drought conditions and exacerbate plant water stress^9^. As droughts and heatwaves are projected to be more frequent and more severe^69–71^, understanding how plants regulate water loss under extreme atmospheric forcing becomes increasingly critical. It remains uncertain whether, under unprecedented heat extremes, plants will maintain stomatal closure to conserve water or whether hydraulic and thermal limits may instead constrain their ability to regulate transpiration. The relative roles of hydraulic depletion (“running out of water”)^72^ versus direct thermal damage (“getting too hot”)^73^ in driving ecosystem-scale canopy decline remain actively debated, as highlighted by recent extreme events such as the 2021 Pacific Northwest heat dome. In this context, the ability to monitor sub-daily ET dynamics at a global scale provides a powerful observational lens. By capturing shifts in the timing and magnitude of midday depression, ECHO-ET offers a means to diagnose plant hydraulic stress responses in near real time and to help disentangle the mechanisms governing vegetation resilience and failure under emerging climate extremes.

Understanding the diurnal cycling of ET can provide insights into how plants adapt to environmental stresses and regulate their mechanisms, which may offer implications for Earth system modeling. As global hourly simulations from Earth system models (ESMs) are not readily available, we analyzed the diurnal cycling of hourly ET from global observation-driven and reanalysis datasets. Compared to ECOSTRESS, the coarse resolution upscaled flux product (FLUXCOM-X; 0.25˚) and meteorological reanalysis dataset (ERA5-Land; 0.1˚) cannot capture the patterns of diurnal shifts in ET, especially in drylands, which likely reflects limitations in its representation of vegetation water stress. FLUXCOM-X cannot adequately resolve the diurnal dynamics of stomatal conductance, plant water stress, and ET. In ERA5-Land, stomatal conductance in land surface schemes is often parameterized as a quasi-steady function of radiation, humidity, and soil moisture, with limited sensitivity to rapid increases in VPD or plant hydraulic constraints^74,75^. Moreover, the coarse spatial resolution of both products mixes heterogeneous land-cover types and contrasting plant functional responses within a pixel, so averaging across differing diurnal ET patterns tends to smear out the actual sub-daily dynamics. These findings highlight the need for improved representation of plant water stress processes in reanalysis products over the course of the diurnal cycle, including stronger VPD sensitivity of stomatal conductance, dynamic plant hydraulic constraints, and tighter coupling between carbon assimilation and transpiration. As droughts and heatwavess are projected to be more severe and more frequent^69–71^, plants will be subject to environmental stresses more regularly^76–78^, and accurately capturing the responses of ecosystem processes to water and heat stresses will be essential for reliably projecting carbon and water cycle dynamics and climate feedbacks into the future.

ET at regional to global scales is typically estimated with remote sensing data derived from polar-orbiting satellites. These studies estimate ET at daily or longer timescales from instantaneous observations at the time of satellite overpass using constant solar radiation or evaporation fraction^79–81^. Our results demonstrate that the diurnal cycling of solar radiation and PET is not synchronized with that of ET. Therefore, using satellite observations at a given time of day may introduce considerable uncertainty into the resulting ET estimate at daily or longer timescales^34,82^. We used ET and LST snapshots from ECOSTRESS version 1.0 in this study. Future studies could benefit from utilizing the ECOSTRESS version 2.0 product, as it offers the potential for improved accuracy and broader spatial and temporal coverage.

In conclusion, we combined observations from a new-generation satellite, ECOSTRESS, and machine learning to reveal the widespread midday depression of ET on a global scale and investigated the effects of water availability and energy limitations on the diurnal pattern of ET in different PFTs and climate regions. Midday depression typically occurred during the peak-growing season in temperate regions, while in the tropics, it aligned with times of water scarcity. In the context of increasing global temperatures and more frequent heatwaves and droughts, our study offers a perspective on the effects of environmental stress on global ecosystems, highlighting that the diurnal asymmetry of ET can be utilized as a direct indicator of the stress perceived by ecosystems on the regional or global scale. This information can help us understand how plants use water over the course of the day, particularly during drought and heatwaves, inform agricultural management, and improve the representation of diurnal cycling of ecosystem processes in Earth system models.

## Methods

### Datasets used

#### ECOSTRESS datasets

The instantaneous ET snapshots across the globe were collected from ECOSTRESS L3_ET_PT-JPL product (ECO3ETPTJPL.001), with spatial resolution of 70 m and repeat time of 1–5 days^17^. This product utilizes the ECOSTRESS land surface temperature and emissivity product (L2_LSTE), in conjunction with atmospheric data from MODIS and surface properties from both MODIS and Landsat. The ECOSTRESS ET is retrieved using the PT-JPL algorithm that employs ecophysiological constraints to adjust potential ET to actual ET^17,83^. The L3 PT-JPL product processed only high-quality raster data, requiring precise geolocation (<50 m) and clear-sky conditions^84^, to minimize the impact of geolocation issues and cloud contamination on the imagery^85–88^. For the exploration of the midday depression, we prioritized quality-controlled ECOSTRESS ET over ECOSTRESS transpiration for the following two reasons: first, the variation of ET is dominated by plant transpiration^33,89^, and second, the partitioning of ET has substantial uncertainties due to the limited observations of transpiration^35,90,91^.

We also used the instantaneous LST snapshots collected from ECO2LSTE v001. For example, over the agricultural fields in the Nile Delta, Egypt (Fig. 5), the ECOSTRESS LST snapshots were acquired in local time on 2018-08-04 (14:03, 22:11), 2018-08-11 (11:12, 19:21), 2018-08-21 (07:22, 15:31), and 2018-08-24 (06:23, 14:32). On the same day, the sampling times were ensured to include periods before and after noon, as well as either evening or early morning. For quality control, we selected grid cells that met the following criteria^40^: (a) flagged as “Pixel produced, best quality” or “Pixel produced, nominal quality” in the quality layer; and (b) flagged as “Cloud Mask Flag determined,” “no Final Cloud Plus Region-growing,” and “no Final Cloud, either one of bits 2, 3, or 4 set” in the cloud mask layer of ECO2CLD v001.

#### ERA5-Land reanalysis dataset

We gathered hourly meteorological data from the ERA5-Land reanalysis dataset^54^, including 2-meter temperature (TA), 2-meter dewpoint temperature, skin temperature, incoming shortwave radiation (SSRD), volumetric soil water content (SWC), surface latent heat flux, and precipitation. Vapor pressure deficit (VPD) was calculated from 2-meter air temperature and dewpoint temperature data. We obtained root-zone soil moisture by calculating a weighted average of volumetric soil water across various depths: 0–0.07 m, 0.07–0.28 m, and 0.28–1 m. Hourly potential evapotranspiration (PET) data were collected from the 0.1° spatial resolution global hPET dataset^92^ derived from the ERA5-Land reanalysis output. The hPET values were computed using the FAO Penman-Monteith equation and net surface solar radiation. Surface latent heat flux, PET and precipitation were used for scientific analysis, while other variables were used for model training. To ensure consistency and compatibility with ECHO-ET on CMG with 0.05° resolution, we resampled all our gridded data to the Climate Modeling Grid (CMG) using the nearest neighbor approach.

#### MODIS datasets

Near-infrared reflectance of vegetation (NIRv), a remotely sensed measure of canopy structure, serves as a key indicator of vegetation characteristics and photosynthetic activity^93,94^. The daily NIRv data were derived from the MODIS MCD43C4.v061 dataset, which offers daily bidirectional reflectance distribution function (BRDF) and albedo nadir BRDF-adjusted reflectance information, at a spatial resolution of 0.05°. To calculate the daily NIRv values, we utilized both the Near-Infrared (NIR) and Red (R) reflectance components^94^:

1 $${{NIR}}_{v}=({NDVI}-0.08)\cdot {NIR}=\left(\frac{{NIR}-R}{{NIR}+R}-0.08\right)\cdot {NIR}$$

Considering that some grid cells had missing reflectance data during some days due to cloud contamination, we employed a temporal filling method to fill the data gaps following Zhao et al.^95^. First, all values less than 0 were set to 0, and values greater than 0.6 were capped at 0.6^93,94^. Then, for each day, we calculated the multiyear mean value of all valid grid cells, using linear interpolation to fill in gaps and ensure spatial-temporal continuity of the multiyear mean. If the first (or last) day’s NIRv value was missing, it was replaced with the multiyear mean. Other missing NIRv data were filled by the linear interpolation, using the nearest reliable values within a 15-day window before and after. If linear interpolation was not feasible due to the lack of valid data within this timeframe, the multiyear mean was used to fill the gap. Two examples of NIRv processing on 2020-01-01 and 2020-08-12 were shown in Fig. S17. Daily NIRv values from 2018 to 2022 were used to help generate the hourly ECOSTRESS ET and to also determine the peak-growing season and the least-active-growing season.

We utilized the MODIS land cover data product (MCD12C1.v006) with the International Geosphere-Biosphere Program (IGBP) classification scheme to identify the vegetation type for each grid cell. Grid cells representing land cover categories unrelated to our research, such as water and ice, were excluded for further analyses.

#### Landsat data

Landsat Collection 2 surface reflectance data, derived from the Landsat 8 OLI/TIRS sensors, were used to calculate NIRv at 70 m for the Nile Delta, Egypt. The NIRv values were computed from Landsat-8 images using cubic resampling to upscale the data from 30  to 70 m. To minimize systematic biases between sensors, linear regression against MODIS NIRv was applied to correct the Landsat-derived NIRv. Landsat 8 images acquired on August 2, 17, and September 2, 2018, were used, and the 70 m NIRv values were then linearly interpolated to generate a continuous daily dataset from August 4 to August 24.

#### Eddy covariance datasets

The eddy covariance (EC) technique is perhaps the best method for directly measuring the exchange of carbon dioxide and water vapor between ecosystems and the atmosphere. It captures data at fine temporal resolutions, typically half-hourly or hourly, providing detailed insights into ecosystem dynamics over time. In this study, ET and meteorological data were collected from 194 EC flux sites across the globe (Supplementary Data 1 and S18a). Most of these sites provided measurements of latent heat flux (i.e., ET), sensible heat flux, net radiation, and soil heat flux, except for a few sites (Nossa Senhora Do Carmo ranch^96^, PE-QFR, and PR-xGU) situated in tropical regions. To achieve energy closure, the flux data were processed by the Open Network-Enabled Flux processing pipeline (ONEFlux)^97^. To conduct comparisons with ECOSTRESS ET, local time was adjusted to local solar time, considering the specific time zone and longitude. Those years with consecutive high-quality observations during the growing season were selected for analysis. In addition, only days with complete observations throughout the day were used. Most sites provided at least three years of data (Fig. S18b).

#### Auxiliary datasets

To support model evaluation and uncertainty analysis, we incorporated independent ET and land cover datasets. The FLUXCOM-X (X-BASE) monthly diurnal cycle of ET was used to provide a global, observation-driven reference of hourly ET dynamics^98^. FLUXCOM-X integrates ET measurements from FLUXNET sites with machine learning models and meteorological forcing to generate spatially continuous ET estimates. In this study, we used the 0.25° resolution product to evaluate large-scale consistency in diurnal patterns of ET variability. Land cover information was derived from the ESA WorldCover product at 10 m spatial resolution^99^. WorldCover provides globally consistent land-cover classifications based on Sentinel-1 and Sentinel-2 observations. We used the 10 m dataset to quantify within-pixel heterogeneity, enabling assessment of scale mismatch effects between tower footprints and coarse-resolution model grids.

### Generation of ECHO-ET

While ECOSTRESS acquires measurements at different times of day, ECOSTRESS samples only a very small portion of the global land surface each day, and it takes months for the instrument to collect sufficient observations over the course of the diurnal cycle for a given location. To examine the diurnal asymmetry of land ET globally, a global gridded, hourly ET dataset that consists of spatially and temporally consistent data is needed. We developed such a gridded, hourly ET dataset (ECHO-ET) using ECOSTRESS snapshots, globally continuous MODIS and meteorological reanalysis data, and a machine learning method. To highlight the capability of fine spatial and temporal resolution ET in representing local surface details and capturing rapid dynamics in water and energy fluxes, the methodology could be modified and applied to specific regions to generate continuous hourly, 70 m ET data. For example, for the agricultural fields in the Nile Delta, Egypt (Fig. 5), we generated hourly, 70 m ET maps from August 4 to August 24, 2018.

#### Selection of sampling blocks

To obtain the global diurnal pattern from ECOSTRESS ET, we introduced the following sampling strategy (Fig. S19). First, we resampled the MODIS land cover map, which was originally at a fine resolution of 0.05°, to a coarser resolution (1°) and grouped certain land cover types with similar structural and functional characteristics. Specifically, we combined evergreen needleleaf forests and deciduous needleleaf forests into needleleaf forests, deciduous broadleaf forests and mixed forests into broadleaf forests, closed shrublands and open shrublands into shrublands, woody savannas and savannas into savannas, and croplands and croplands/natural vegetation mosaic into croplands, respectively. Second, we selected 100 distinct 1° × 1° blocks (Fig. S20). This selection was based on the proportional representation of various land cover types present within the ECOSTRESS coverage area, which spans from 52°S to 52°N. Despite the largest percentage of barren lands, only five blocks were selected in these areas, ensuring a balanced representation. Additionally, the climate types within the selected blocks closely mirrored the overall climatic distribution between 52°S and 52°N (Fig. S21). Third, we downloaded quality-controlled ECOSTRESS ET snapshots for each of these selected blocks, utilizing the Application for Extracting and Exploring Analysis Ready Samples (AρρEEARS) API (https://appeears.earthdatacloud.nasa.gov/). Fourth, we resampled all downloaded 70 m resolution ECOSTRESS ET snapshots into the 0.05° resolution CMG. A 0.05° grid cell was considered valid and adopted only if it had ECOSTRESS ET data for at least 95% of the 70-m pixels within the grid cell. Finally, we calculated the average values of ET snapshots within these 0.05° grid cells. This process resulted in a total of 1,385,888 valid 0.05° grid cells (Table S1). The distribution of these 0.05° ECOSTRESS ET samples across UTC hours and months are shown in Fig. S22.

#### Model training and validation

We used a machine learning method, the Random Forest (RF) algorithm^100^, to develop the predictive ET model. The model consisted of 100 trees and minimized mean squared error. All predictor variables were included without prior feature elimination, allowing the model to account for nonlinear relationships and feature interactions. Bootstrap aggregation was applied during training, with no constraint on tree depth and a minimum leaf size of one. The target variable of the model was ET, and the explanatory variables included TA, skin temperature, SSRD, root-zone SWC, VPD, and NIRv. All the variables except NIRv were at hourly time steps. We did not include precipitation and leaf area index (LAI) in the baseline model. Precipitation primarily affects ET through its influence on root-zone soil moisture, which already integrates antecedent precipitation and hydrologic memory^49^. Similarly, LAI is strongly correlated with NIRv and related vegetation proxies^101^. To ensure the model’s accuracy across different land cover types, we allocated 70% of the dataset from each land cover type, including numerous grid cells with ET and all explanatory variable data, for training, while reserving the remaining 30% for validation purposes. The validation (Fig. S23 and Table S2) showed that simulated ET was strongly correlated with ECOSTRESS ET with a Coefficient of Determination (r^2^) of 0.95 (*p* < 1 × 10⁻³⁰⁰, sample size = 415,773). Despite minor dispersion, the model demonstrated notable predictive accuracy, as indicated by the low Root Mean Squared Error (RMSE) of 29 W m^-2^ and Percent Bias (PBIAS) of 0.08%. Based on this, a global, gridded hourly ET dataset with a spatial resolution of 0.05° for 2018–2022 was generated using the trained RF model and globally continuous data of 2-meter temperature, skin temperature, incoming shortwave radiation, root-zone soil moisture, VPD, and NIRv. Benchmarking against observed values from EC, the simulation reported r^2^ of 0.65 (*p* < 1 × 10⁻³⁰⁰, sample size = 169,560), RMSE of 94 W m^-2^, and PBIAS of 147.67% (Fig. S24a).

#### ET correction

As noted in the comparison with flux data (Fig. S24a), the simulated ET values showed systematic overestimation (PBIAS = 147.67%). This is mainly because the ECOSTRESS L3_ET_PT-JPL ET product that we used exhibits a systematic overestimation issue. The systematic bias of the ECOSTRESS L3_ET_PT-JPL ET product has been documented by several previous studies^29,40,102–105^. To address this systematic error, we utilized the observed latent heat fluxes from 194 global flux sites to implement multiple sets of linear fitting (Fig. S25). The datasets were divided into various groups using phenological and daytime/nighttime thresholds. The phenological thresholds were determined using NIRv, with intervals of [0, 0.2, 0.4, 0.6, 0.8] multiplying the maximum value of NIRv at each site. Daytime and nighttime thresholds were determined using incoming shortwave radiation, with values greater than 5 W m^-2^ categorized as ‘daytime’ and those less than 5 W m^-2^ as ‘nighttime.’ All datasets were adjusted to local solar time. Ten groups per biome were obtained based on phenological and daytime/nighttime thresholds. For each biome, we used data from 70% of the sites to build the model (forming the calibration sets), while the remaining 30% were reserved for model validation (forming the validation sets). Within each calibration set group, we developed various regression models, including linear (y=kx+b), offset (y = x + m), upscale (y=kx), and original (y = x) models. The optimal model was selected to achieve the lowest mean absolute error when comparing corrected and observed ET within the validation sets. As shown in Fig. S24, after processing the ET data using optimal models, we observed a notable increase in r^2^ from 0.65 to 0.72 (*p* < 1 × 10⁻³⁰⁰, sample size = 169,560) along with a substantial reduction in both RMSE (from 94 to 45 W m^-2^) and PBIAS (from 147.67% to –1.02%) for monthly diurnal ET. Regarding potential biases during the first or last years of site operation, we performed a sensitivity analysis excluding the initial and final years of each EC site record to assess potential start-of-operation calibration issues or end-of-operation sensor degradation and malfunctions. The model was re-evaluated after removing these boundary years. The validation metrics after excluding the first and last years of each site record (RMSE = 45.004 W m^–2^, *r*^2^ = 0.718, *p* < 1 × 10⁻³⁰⁰, sample size = 124,464) were virtually identical to those obtained from the full-record analysis, indicating negligible influence of potential start- or end-of-operation biases. This indicates that potential early- or late-stage instrumental adjustments do not materially affect the calibration or validation of ECHO-ET. Despite the lack of ECOSTRESS coverage in high-latitude regions (beyond 52°N), the model remained reliable, as demonstrated by a comparison with measurements from 28 flux towers, achieving *r*^2^ = 0.63 (*p* < 1 × 10⁻³⁰⁰, sample size = 24,864) and RMSE = 31 W m^-2^ (Fig. S26). Compared with uncorrected ET, corrected ET had a more similar frequency distribution to flux tower data (Fig. S27). This further validates the effectiveness of the correction procedures. The time series of ET was also evaluated using flux tower data. Figure S28 compared the monthly diurnal variations in ET in 12 biomes. The graphs suggested that the corrected ET generally aligned well with the measured data, as seen by the overlapping lines, indicating the effectiveness of the correction process in accurately estimating ET.

We compared our ECHO-ET product with two widely used global hourly ET datasets, including an observation-driven product (FLUXCOM-X) and a reanalysis dataset (ERA5-Land hourly). Figure S29 showed that ECHO-ET exhibited strong positive agreement with both FLUXCOM-X (*r*^2^ = 0.85, RMSE = 34 W m⁻², *p* < 1 × 10⁻³⁰⁰, sample size = 65,572,106) and ERA5-Land hourly (r^2^ = 0.80, RMSE = 36 W m⁻², *p* < 1 × 10⁻³⁰⁰, sample size =  412,319,539) at the monthly-averaged diurnal scale, with slightly better consistency observed for FLUXCOM-X.

#### Generating local hourly ET at 70 m

The ECHO-ET framework can be applied to specific regions to generate 70-m hourly ET estimates. Our case study region is the Nile Delta, Egypt. Producing continuous hourly ET at 70 m resolution relied on obtaining spatiotemporally consistent LST at the same resolution as input data for the model^40^. It is worth noting that since the input variable for the ML model is the ERA5-Land hourly skin temperature, what we have essentially constructed is a downscaled ERA5-Land hourly skin temperature. Although surface skin temperature shares the same physical concept as the LST retrieved from satellites^106^, there may be some systematic biases between the two, especially in areas with sparse observational data. Therefore, using linear regression and the ERA5-Land hourly skin temperature at the corresponding time, we first corrected the ECOSTRESS LST snapshots (hereafter, all references to ECOSTRESS LST refer to the corrected values).

The diurnal temperature cycle (DTC) model^107^ was employed to reconstruct the diurnal cycle of LST under clear-sky conditions. Here, we used the model improved by Inamdar et al.^108^ to reconstruct the full diurnal cycle of LST under clear-sky conditions. It describes the daytime LST as a harmonic function and the nighttime LST as a hyperbolic decay, based on the principles of thermal diffusion and Newton’s law of cooling, respectively^109,110^. This DTC model is expressed as:

For t<t_s_ (daytime):

2 $$T\left(t\right)={T}_{0}+{T}_{a}\cos \left(\frac{\pi }{\omega }\left(t-{t}_{\max }\right)\right)$$

For t≥t_s_ (nighttime):

3 $$T\left(t\right)=\left({T}_{0}+{\delta }_{T}\right)+\left[{T}_{a}\cos \left(\frac{\pi }{\omega }\left({t}_{s}-{t}_{\max }\right)\right)-{\delta }_{T}\right]\frac{k}{k+t-{t}_{s}}$$

where T_0_ is residual temperature, approximately equivalent to the temperature at sunrise. T_a_ is temperature amplitude. ω is the width corresponding to ±π/2 (half the period of the cosine term). ${{{\rm{t}}}}_{\max }$ is time at which the maximum temperature occurs. ${{{\rm{t}}}}_{{{\rm{s}}}}$ is start time of the attenuation function. ${{\rm{k}}}$ is attenuation constant. ${{{\rm{\delta }}}}_{{{\rm{T}}}}$ is difference between the ${{{\rm{T}}}}_{0}$ and the temperature as time (t) approaches infinity, i.e., T_0_-T(t→∞). The calculation of these variables is described by Collins et al.^111^. and Hu et al.^110^.

We estimated key time- and temperature-related parameters using ECOSTRESS and ERA5-Land data. The time-related parameters, ${{{\rm{t}}}}_{\max }$ (time of maximum LST) and ${{{\rm{t}}}}_{{{\rm{s}}}}$ (time of thermal sunset marking the start of nighttime cooling), were derived from ERA5-Land hourly skin temperature and assumed constant within each ERA5-Land grid cell over a 21-day window^40^. Following Hu et al.^110^, the temperature-related parameters included T_sr,d_ and T_sr,d+1_, the LST at sunrise on days *d* and *d*+1, respectively, and T_max,d_, the maximum LST observed during the day. We leveraged the high spatial resolution of ECOSTRESS LST while using ERA5-Land to capture temporal variations^40^. Given the sparse observations from ECOSTRESS, with only eight scenes over four days in the study window, we fitted all days together. We also assumed that day-to-day variations in ${{{\rm{T}}}}_{{{\rm{sr}}},{{\rm{d}}}}$, ${{{\rm{T}}}}_{\max,{{\rm{d}}}}$, and last-day ${{{\rm{T}}}}_{{{\rm{sr}}},{{\rm{d}}}+1}$ for fine-resolution ECOSTRESS grid cells matched those in coarse-resolution ERA5-Land grid cells. We adapted an approach from Duan et al.^112,113^, reconstructing the diurnal LST curve using ERA5-Land temperatures at 8.50, 14.50, 20.50, and 2.50 h local time. The point closest to the ECOSTRESS overpass time was replaced with ECOSTRESS measurements to ensure that the daytime portion of the curve was primarily determined by ECOSTRESS LST. To refine the parameters, we applied the simulated annealing algorithm^114,115^, minimizing the RMSE between simulated and observed LST values from ECOSTRESS and ERA5-Land. This approach ensured a well-calibrated diurnal temperature cycle despite limited ECOSTRESS observations. For the agricultural fields in the Nile Delta, Egypt (Fig. 5), the leave-one-out validation results yielded a strong agreement between reconstructed LST and calibrated ECOSTRESS LST, with *r*^2^ = 0.97 (*p* < 1 × 10⁻³⁰⁰, sample size = 500,756), RMSE of 2.49 K, and a minimal PBIAS of 0.17% (Fig. S30). After generating the diurnal cycle of LST at 70 m from 4 to 24 August 2018, we used the trained RF model along with 70 m inputs of downscaled ERA5-Land hourly datasets (air temperature, incoming shortwave radiation, root-zone soil moisture, and VPD), Landsat NIRv, and the reconstructed LST to produce the diurnal cycle of ET at the same resolution and time window.

### Uncertainty analysis of ECHO-ET

#### Conformal prediction intervals

To characterize predictive uncertainty, we derived calibrated prediction intervals (PIs) for the RF-based ET estimates using a split conformal prediction framework^116,117^. Conformal prediction was adopted because it provides distribution-free, finite-sample prediction intervals with guaranteed empirical coverage and independent of model complexity. The training data were divided into a model-fitting subset (80%) and an independent calibration subset (20%).

To account for model variability, we quantified uncertainty using the tree-level ensemble within a single random forest model trained on the fitting subset using bootstrap aggregation and default hyperparameters. For a given input x, predictions from the 100 trees (equal to the number of estimators in the forest) form an empirical distribution ${\left\{{\bar{{{\rm{y}}}}}^{\left({{\rm{b}}}\right)}\left({{\rm{x}}}\right)\right\}}_{{{\rm{b}}}=1}^{100}$, from which we computed the ensemble mean $\bar{{{\rm{y}}}}\left({{\rm{x}}}\right)$ and the across-tree standard deviation ${{{\rm{\sigma }}}}_{{{\rm{tree}}}}\left({{\rm{x}}}\right)$.

The training data were divided into a model-fitting subset and an independent calibration subset. Calibration was performed by defining normalized nonconformity scores

4 $${s}_{i}=\frac{\left|{y}_{i}-\bar{y}\left({x}_{i}\right)\right|}{{\sigma }_{{tree}}\left({x}_{i}\right)}$$

and selecting a global scaling factor q as the finite-sample split-conformal $\left(1-{{\rm{\alpha }}}\right)$-quantile of $\left\{{{{\rm{s}}}}_{{{\rm{i}}}}\right\}$. All results are reported using a nominal 90% prediction interval $\left({{\rm{\alpha }}}=0.10\right)$. The resulting prediction interval for a new input x is

5 $${{PI}}_{1-\alpha }\left(x\right)=\left|\bar{y}\left(x\right)-q{\sigma }_{{tree}}\left(x\right),\bar{y}\left(x\right)+q{\sigma }_{{tree}}\left(x\right)\right|$$

Empirical coverage was evaluated on an independent test set and found to be consistent with the nominal coverage level. For large-scale mapping, uncertainty was summarized using the prediction interval half-width,

6 $${Half}\,{{PI}}_{1-\alpha }\left(x\right)=q{\sigma }_{{tree}}\left(x\right)$$

which was aggregated spatially and temporally to produce maps of predictive uncertainty.

Overall, daily-average hourly PIs showed coherent latitudinal gradients and strong seasonality (Fig. S31). Importantly, although ECOSTRESS has limited spatial coverage at high latitudes due to orbital constraints, the maps did not show systematically elevated PIs in those regions. High-latitude areas did not exhibit larger uncertainty compared to mid-latitude or semi-arid regions. This indicates that the reduced observational coverage of ECOSTRESS in high-latitude zones did not lead to amplified prediction intervals in the global product.

#### Uncertainty quantification of ET correction using eddy covariance measurements

To rigorously quantify observational and model-related uncertainties in ECHO-ET, we implemented an uncertainty-aware calibration framework based on EC measurements. We used energy-balance-closure corrected EC ET from the ONEFlux dataset. Measurement uncertainty was explicitly propagated using the joint uncertainty estimate, which integrates both random measurement errors and uncertainties associated with the energy balance closure correction. For sites lacking reliable uncertainty estimates, we adopted a conservative fallback uncertainty defined as 20% of the absolute ET, while preserving missing values. Uncertainty propagation was performed independently for each site using a Monte Carlo approach. For each realization, EC ET values were randomly sampled within their uncertainty bounds (corrected EC ET ± uncertainty), and a regression model was fitted between the sampled EC ET and uncorrected ET. This procedure was repeated 1000 times, yielding an ensemble of corrected ECHO-ET estimates. The resulting ensemble distribution was then used to derive the hourly mean and standard deviation of corrected ECHO-ET.

Uncertainty magnitudes exhibited a clear and consistent diurnal cycle across all vegetation types, with minimal uncertainty at night and pronounced peaks around noon (Fig. S32). Croplands and savannas showed the largest uncertainty in summer, whereas barren and sparsely vegetated regions exhibited comparatively small amplitudes.

#### Influence of EC footprint and ECHO-ET grid scale mismatch

To mitigate the remaining scale mismatch between EC tower footprints (hundreds of meters) and the coarser 0.05° ECHO-ET grid, we filtered sites based on within-pixel land-cover heterogeneity. Each tower was first located within its corresponding 0.05° ECHO-ET grid cell, and land-cover fractions were derived from the WorldCover (10 m) product to determine the dominant class and its proportional coverage. In addition, we quantified the dominant land-cover fraction within a 1 km radius centered on each EC tower.

Site subsets were constructed by applying dominant-class ratio thresholds of 0, 0.3, 0.4, 0.5, 0.6, 0.7, and 0.8. For each threshold, we further generated a stricter subset retaining only sites where the dominant land-cover class in the 0.05° ECHO-ET grid cell matched the dominant class within the 1 km tower-centered buffer. The hourly RMSE and R² values, as validated by EC measurements (Supplementary Data 1), were averaged for each subset. Across all homogeneity thresholds, hourly RMSE remained relatively stable (58–61 W m⁻²), and hourly R² ranged from 0.53 to 0.56 (Fig. S33). Increasing spatial homogeneity led to only marginal reductions in model error at the hourly scale. These modest improvements indicate that although scale mismatch contributes to prediction uncertainty, it is not the primary source of model error.

### Determination of the peak-growing season and the least-active-growing season

We used ECHO-ET over the period from January 2018 to December 2022 to investigate the diurnal variations of ET over the peak-growing season and the least-active-growing season. We identified the three months with the highest and lowest values of the NIRv within each grid cell as the peak-growing season and least-active-growing season, respectively, following Cooley et al.^45^.

Figure S3 illustrates the global maps of peak-growing season and least-active-growing season. For example, in temperate regions, the peak-growing season mainly occurred during summer (June to August in the Northern Hemisphere, December to February in the Southern Hemisphere), while the least-active-growing season predominated in winter (December to February in the Northern Hemisphere, June to August in the Southern Hemisphere). However, in tropical regions and some drylands, the peak-growing season was closely related to precipitation with the peak-growing season occurring during the rainy season while the least-active-growing season during the dry season. For example, the Indian subcontinent had the peak-growing season from August to October and the least-active-growing season from April to June. The Sahel region had its peak-growing season from August to October and its least-active-growing season from March to June. In Mediterranean climate regions, the peak-growing season primarily occurred during the warm and rainy winter and spring, while the least-active-growing season fell in the hot and dry summer. The Amazon region had a peak-growing season from September to April, and the least-active-growing season primarily during the dry season from May to July, varying by location.

Figure S34 demonstrates enhanced ET from 6.00 to 18.00 h local solar time during the peak-growing season, with the strongest activity during midday. This pattern was most apparent in the regions with more vegetation and thus more active ET during these peak hours.

### Determination of the energy-limited and water-limited regions

According to McVicar et al.^50^, the global distribution of energy-limited and water-limited areas was determined based on the annual average ratio of precipitation to PET (i.e., P/PET) from 2018 to 2022 (Fig. 3a). Precipitation was assumed to be the sole source of water. Regions where the P/PET ratio exceeded 1.0 were classified as energy limited, indicating that ET was limited by energy rather than water availability. Conversely, regions where the ratio was less than 1.0 were classified as water-limited, indicating that ET was constrained by water availability rather than energy. Water-limited regions are mostly located in arid or semi-arid regions, while energy-limited regions are more likely to be found in temperate or high latitude regions.

### Metrics for diurnal variation of ET

To characterize diurnal variations of ET and to measure its diurnal asymmetry (or symmetry), we calculated the centroid of the sub-daily curve as a diurnal indicator. Diurnal centroid is an effective metric used to assess the diurnal shift in ecosystem functioning caused by environmental stress^19,24,34^. The diurnal centroid (h) of ET was characterized as follows,

7 $${Diurnal}\,{Centroid}=\frac{{\sum }_{t=t1}^{t2}\left({{ET}}_{t}\cdot t\right)}{{\sum }_{t=t1}^{t2}\left({{ET}}_{t}\right)}$$

where t is a regular hourly time interval.

To derive a robust mean diurnal cycle, raw hourly ET estimates were first averaged by month for each grid cell, spanning 2018–2022. The monthly mean hourly ET values were then used to calculate the diurnal centroid of ET (C_ET_; Fig. S1), with barren lands excluded from the analysis. For seasonal analyses, monthly mean hourly ET values were further aggregated to seasonal mean diurnal cycles before calculating C_ET_. We did not convert negative hourly ET values to zero before aggregation, because such thresholding may introduce an artificial bias to the diurnal pattern inherent in the ECHO-ET estimates and the ECOSTRESS ET, particularly in low-ET regions or periods where negative values may arise from uncertainty in the linear ET correction procedure (Fig. S28). The time range was set from 6.00 to 18.00 h local solar time (t1 = 6.50 h local solar time and t2 = 17.50 h local solar time) in this study. Centroid values falling outside this range were excluded from subsequent analyses. The C_ET_ derived from the gridded ET data closely matched that at EC flux sites globally (Fig. S1), including the above 60°N region where the ECOSTRESS cannot cover (Fig. S35).

To prevent errors resulting from the selected time range, we also calculated C_ET_ from 7.00 to 17.00 h local solar time (Fig. S36). The global patterns of C_ET_ based on the two timespans were very similar. We also calculated the diurnal centroid of PET (C_PET_) using the hourly PET data based on ERA5-Land (Fig. S5). The diurnal patterns of PET and ET were contrasted using the difference in diurnal centroid between PET and ET (i.e., C_PET_ minus C_ET_) (Fig. S6).

To demonstrate the reliability of the results based on diurnal centroid, we calculated the diurnal change between morning and afternoon ET for comparison (Fig. S37). The diurnal change in ET (%) between mean morning ET (ET_AM_) and mean afternoon ET (ET_PM_) was characterized as follows^29,34^,

8 $${Diurnal}\,{Change}=\frac{{{ET}}_{{PM}}-{{ET}}_{{AM}}}{{{ET}}_{{AM}}}\cdot 100\%$$

### Comparison of ET and PET

PET minus ET (PET-ET) and the ratio of ET to PET (ET/PET) were used as the key indicators for quantifying the difference between PET and ET. The combined effect of a larger PET-ET and a smaller ET/PET reveals a stronger suppression of ET. For each grid cell, we calculated PET-ET and ET/PET on a monthly-average hourly basis. Based on the results, we identified the maximum PET-ET and the minimum ET/PET each month. These values were then averaged annually for each grid cell, as illustrated in Fig. 2a–d. Additionally, the standard deviation of monthly minimum ET/PET was computed and presented in Fig. 2e, f.

### Statistical methods

To explore the differences in diurnal variations across energy and water-limited regions, we employed the Kolmogorov-Smirnov Test (KS Test)^118^ and Cohen’s d^119^ effect size value. The Kolmogorov-Smirnov (KS) test is a non-parametric statistical test used to determine whether two distributions differ significantly. The two-sided two-sample K-S test assumes that the two samples are drawn from the same distribution,

9 $${KS}=max \left|{F}_{1}\left(x\right)-{F}_{2}\left(x\right)\right|$$

where ${{{\rm{F}}}}_{1}\left({{\rm{x}}}\right)$ and ${{{\rm{F}}}}_{2}\left({{\rm{x}}}\right)$ are the empirical distribution functions of the two samples. The KS statistic ranges from 0 to 1, with a larger value indicating a greater discrepancy between the two distributions. Additionally, the KS test yields a *p* value to determine whether the null hypothesis should be rejected. If the *p* value is less than the chosen significance level, typically 0.05, it is concluded that there is a statistically significant difference between the distributions.

Cohen’s d effect size was utilized to measure the magnitude of difference between the means of two groups,

10 $${Cohen} ^\prime s\,d=\frac{\overline{{x}_{1}}-\overline{{x}_{2}}}{{SD}}$$

where $\bar{{{{\rm{x}}}}_{1}}$ and$\bar{{{{\rm{x}}}}_{2}}$ are the means of the two groups, and SD is the standard deviation. A larger absolute value of Cohen’s d values of denotes a more substantial divergence. The categories start at 0.01 for “Very small”, 0.20 for “Small”, 0.50 for “Medium”, 0.80 for “Large”, 1.20 for “Very large”, and 2.0 for “Huge”.

We applied the KS test and Cohen’s d effect size to compare the distributions of the diurnal centroid between the peak-growing season and least-active-growing season in regions with water and energy constraints in temperate and tropical zones.

### Definition of drought and heatwave events

Drought events were identified at the grid level using the Palmer Drought Severity Index (PDSI). A drought month was defined as a month with PDSI ≤ −2.0, corresponding to at least moderate drought conditions. Months with PDSI ≥ 0.49 were classified as near-normal conditions and used as the non-drought reference state, whereas months falling between these thresholds were excluded from both drought and reference categories. The daily evolution of drought was characterized using daily Standardized Precipitation–Evapotranspiration Index (SPEI) in Fig. 7. Heatwave days were defined independently at each grid cell to account for local climate variability^23^. Specifically, a heatwave day was identified when (i) the daily maximum air temperature (Tmax) exceeded the 90th percentile of the historical Tmax distribution during 2000–2022 at that pixel, and (ii) the daily Tmax anomaly exceeded the multi-year (2000–2022) mean daily Tmax by at least 5 °C. Heatwave events were then defined as temporally continuous sequences of at least seven consecutive heatwave days; shorter sequences were excluded to remove short-lived or transient temperature extremes. All anomaly calculations were conducted only during the peak-growing season. For each identified heatwave or drought event, event-level anomalies were computed as the difference between the mean value of the C_ET_ during the event period and a corresponding multi-year daily (for heatwaves) or monthly (for drought) climatology representing normal conditions. Specifically, the event-level anomaly was defined as:

11 $${\triangle X}_{{event}}=\overline{{X}_{{event}}}-\overline{{X}_{{normal}}}$$

where $\overline{{\rm{X}}_{{\rm{event}}}}-\overline{{\rm{X}}_{{\rm{normal}}}}$ denotes the mean value of C_ET_ during the event period, and X_normal_ represents the climatological mean under normal conditions. For heatwave events, X_normal_ was calculated as the multi-year daily climatology corresponding to the same calendar days. For drought events, X_normal_ was defined as the multi-year monthly climatology for the same calendar month.

### SHAP-based attribution of meteorological drivers of diurnal ET

To quantify the meteorological controls on changes in the diurnal cycle of ET, we employed a SHapley Additive exPlanations (SHAP)^120^–based attribution framework coupled with an RF regression model. The RF model was trained to predict anomalies in C_ET_ using concurrent anomalies in SSRD, root-zone SWC, TA, and VPD. All variables were expressed as anomalies relative to multi-year climatological means to remove seasonal background effects and isolate stress-related deviations.

The attribution analysis was conducted during the peak- growing season. An event-focused analysis pooled all grid cells and months (days) associated with identified drought (heatwave) events, allowing us to identify the dominant model-attributed drivers of C_ET_ anomalies under extreme hydroclimatic stress. SHAP values were computed using a TreeExplainer with an interventional feature perturbation scheme, which decomposes the RF predictions into additive contributions from each meteorological variable while accounting for nonlinear responses and interactions among predictors. Positive SHAP values indicate that a given variable contributes to a later C_ET_, whereas negative SHAP values indicate a contribution to an earlier C_ET_. By aggregating SHAP values across all samples, we derived global importance rankings and dependence relationships that provide process-relevant insight into how atmospheric demand, radiation, temperature, and soil moisture jointly regulate diurnal ET responses across space and time.

### Reporting summary

Further information on research design is available in the Nature Portfolio Reporting Summary linked to this article.

## Supplementary information

Supplementary Information

Peer Review file

Description of Additional Supplementary Files

Supplementary Data 1

Reporting Summary

## Data Availability

The data used for this analysis are available at Zenodo: [10.5281/zenodo.20790910]. The ECHO-ET dataset generated in this study is listed on the Global Ecology Group website (https://globalecology.unh.edu/data.html) and has been deposited in the Global Ecology Data Repository (https://data.globalecology.unh.edu/ECHO-ET). All other sources of open access data used in this study are listed in Table S3.

## Code availability

The custom code used for data processing, model training and prediction, statistical analyses, and figure generation in this study is available at Zenodo: [10.5281/zenodo.20790910]. The repository includes the scripts necessary to reproduce the main analyses and figures reported in this manuscript.
