# Supplementary material for: NASA’s ECOSTRESS satellite reveals widespread midday depression in ecosystem evapotranspiration
Source: Nat Commun. 2026 Aug 25;17:8976. doi: 10.1038/s41467-026-76873-x (PMC13507112; doi:10.1038/s41467-026-76873-x)
Supplement: Supplementary file 1 [file 41467_2026_76873_MOESM1_ESM.pdf]

Supplementary Information for

NASA's ECOSTRESS satellite reveals widespread midday depression in ecosystem evapotranspiration

Jingyi Bu[1], Jingfeng Xiao[1*], Joshua B. Fisher[2,3], Yiqi Luo[4]

[1] Earth Systems Research Center, Institute for the Study of Earth, Oceans, and Space, University of New Hampshire, Durham, NH 03824, USA

[2] Schmid College of Science and Technology, Chapman University, Orange, CA 92866, USA

[3] Hydrosat, Inc., Washington, DC, 20036, USA

[4] Soil and Crop Sciences Section, School of Integrative Plant Science, Cornell University, Ithaca, NY 14853, USA.

*Correspondence to j.xiao@unh.edu

This PDF file includes:

Figs. S1 to S37
Tables S1 to S3

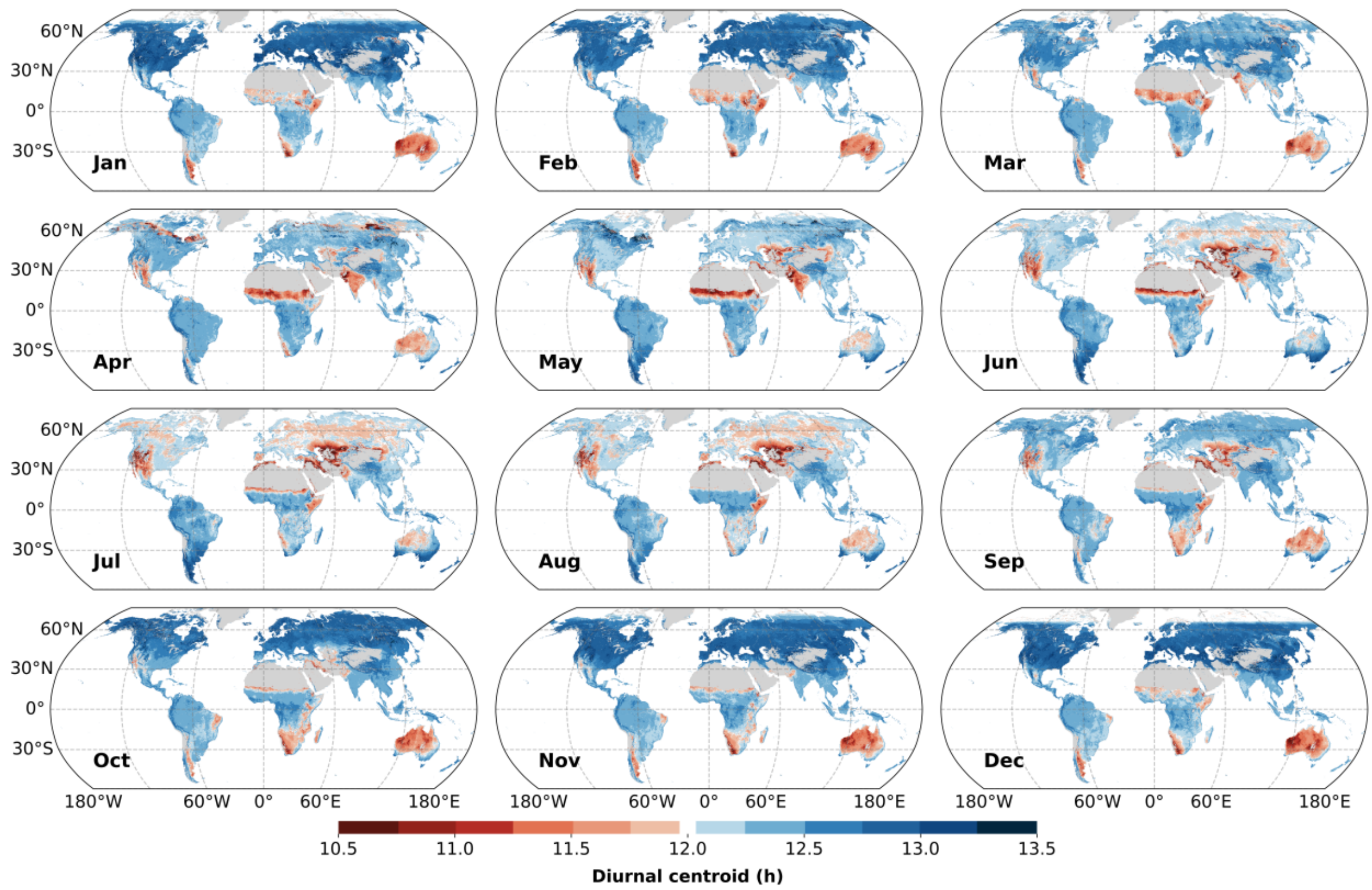

Fig. S1 Global patterns of the diurnal centroid of ET (C_{ET}) (6-18 h local solar time) derived from ECHO-ET dataset.

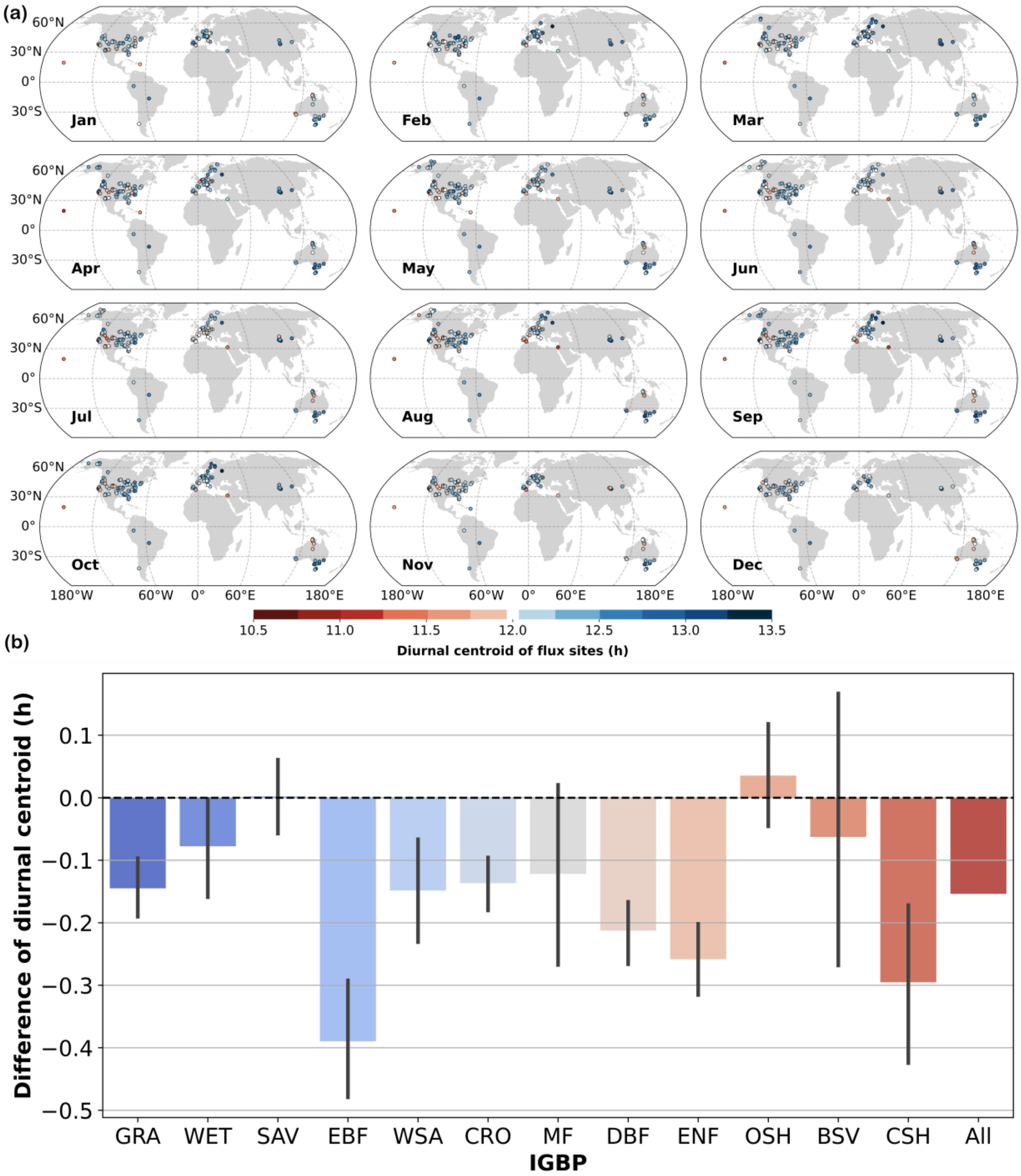

Fig. S2 (a) Global patterns of the diurnal centroid for ET (C_{ET}), derived from eddy covariance (EC) flux tower data. Records where the diurnal maximum is less than 20 W m^{-2} and the coefficient of variation is less than 5% were removed. (b) Difference of diurnal centroid from EC ET and ECHO-ET across Plant Functional Types (PFTs), GRA: Grasslands, WET: Permanent Wetlands, SAV: Savannas, EBF: Evergreen Broadleaf Forest, WSA: Woody Savannas, CRO: Croplands, MF: Mixed Forests, DBF: Deciduous Broadleaf Forest, ENF: Evergreen Needleleaf Forest, OSH: Open Shrublands, BSV: Barren sparse vegetation, CSH: Closed Shrublands, All: all PFTs.

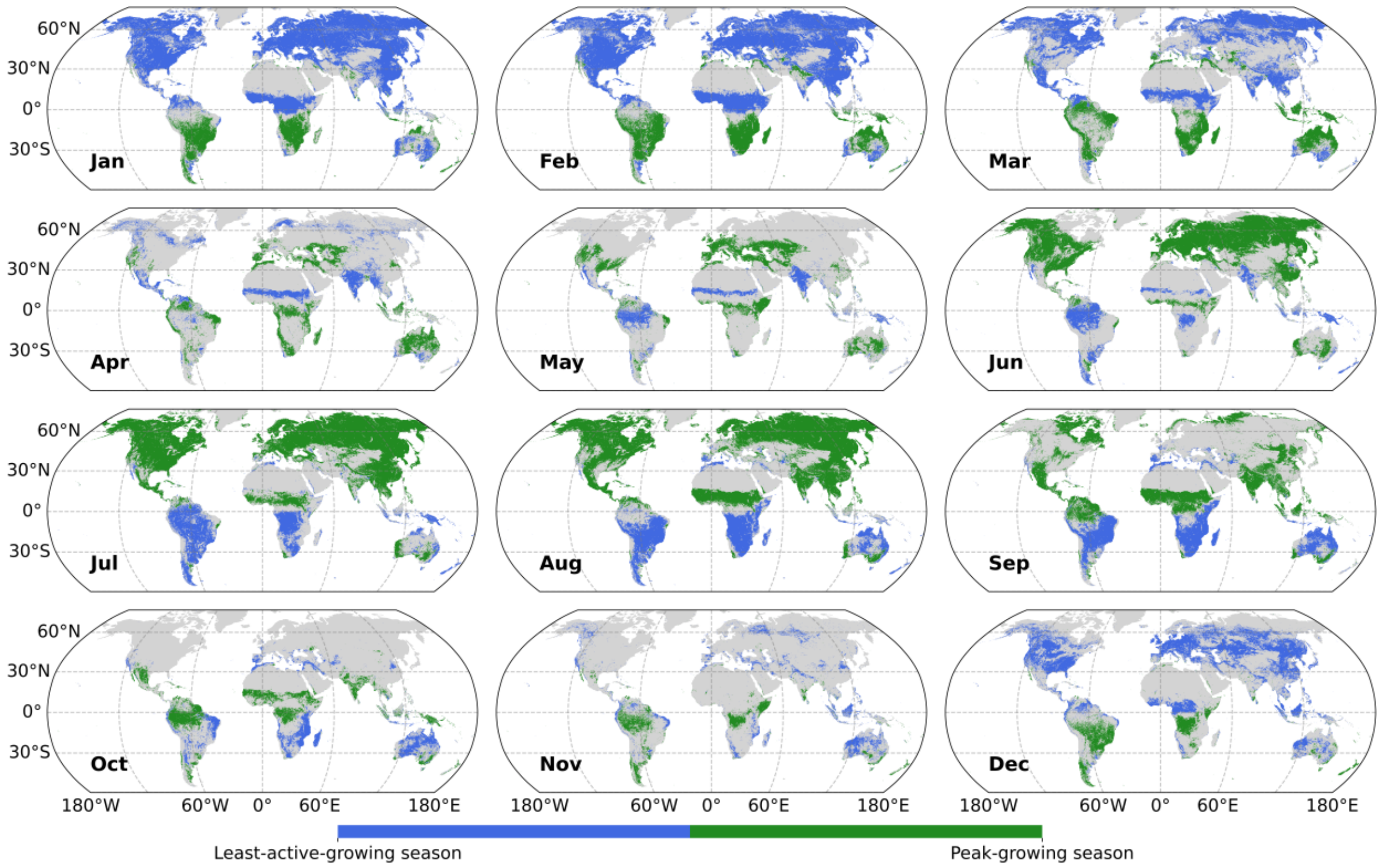

Fig. S3 Global maps of peak-growing season and least-active-growing season, derived from the NIRv index.

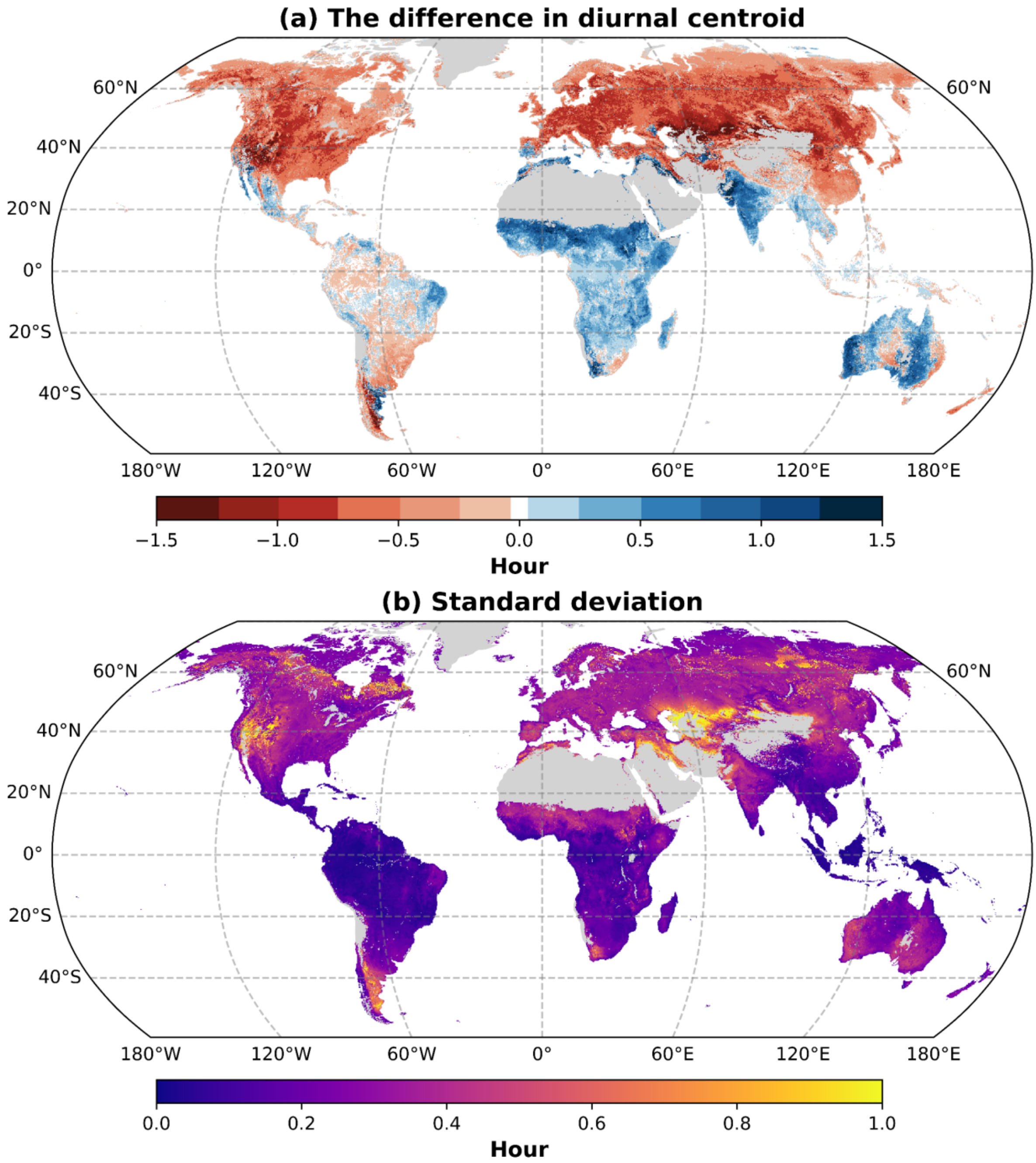

Fig. S4 (a) The difference in the diurnal centroid of ET (C_{ET}) between the peak-growing and least-active-growing seasons. (b) The standard deviation of monthly C_{ET}.

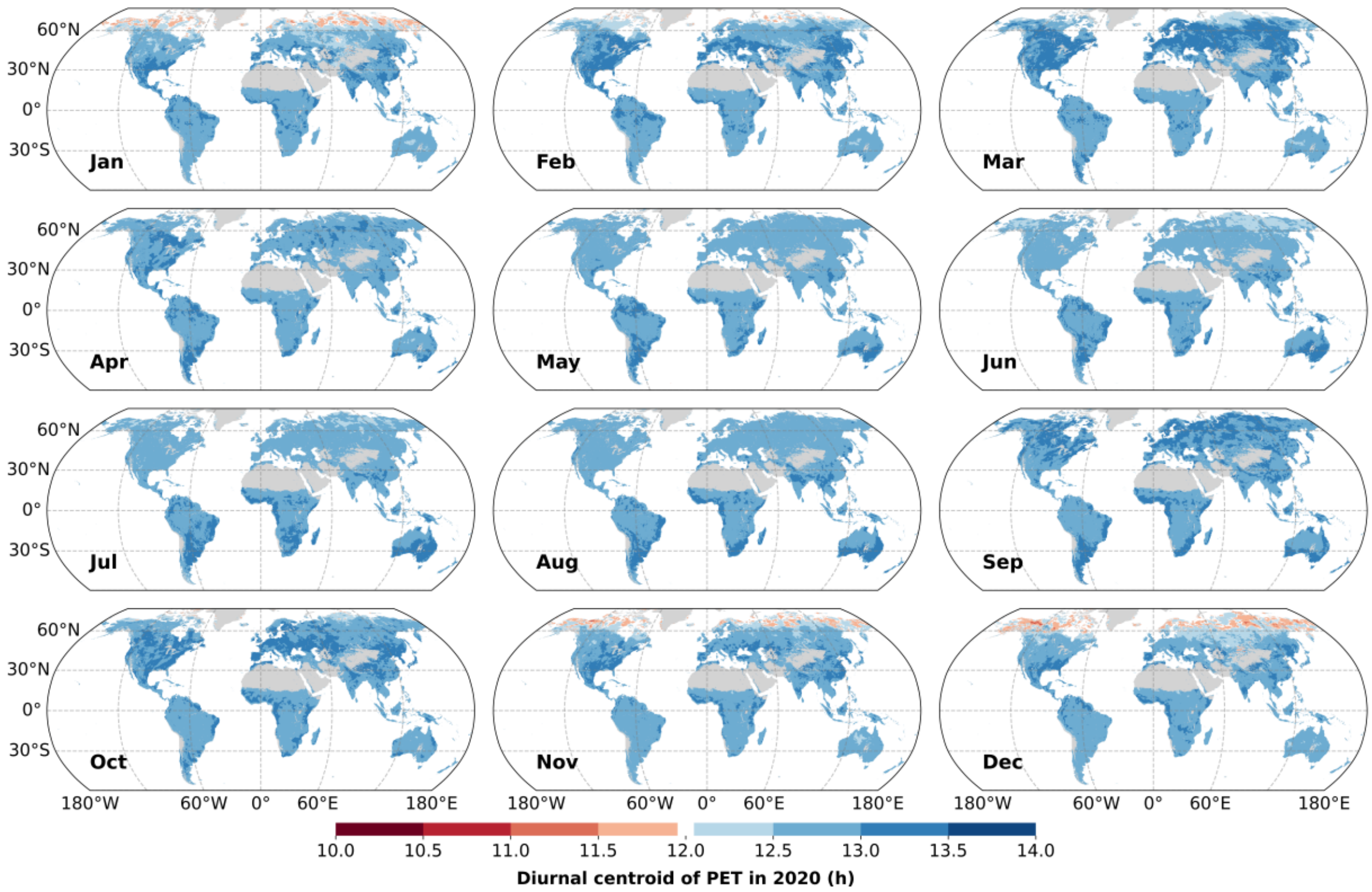

Fig. S5 Global patterns of the diurnal centroid for potential ET (C_{PET}) in 2020, derived from the hPET dataset.

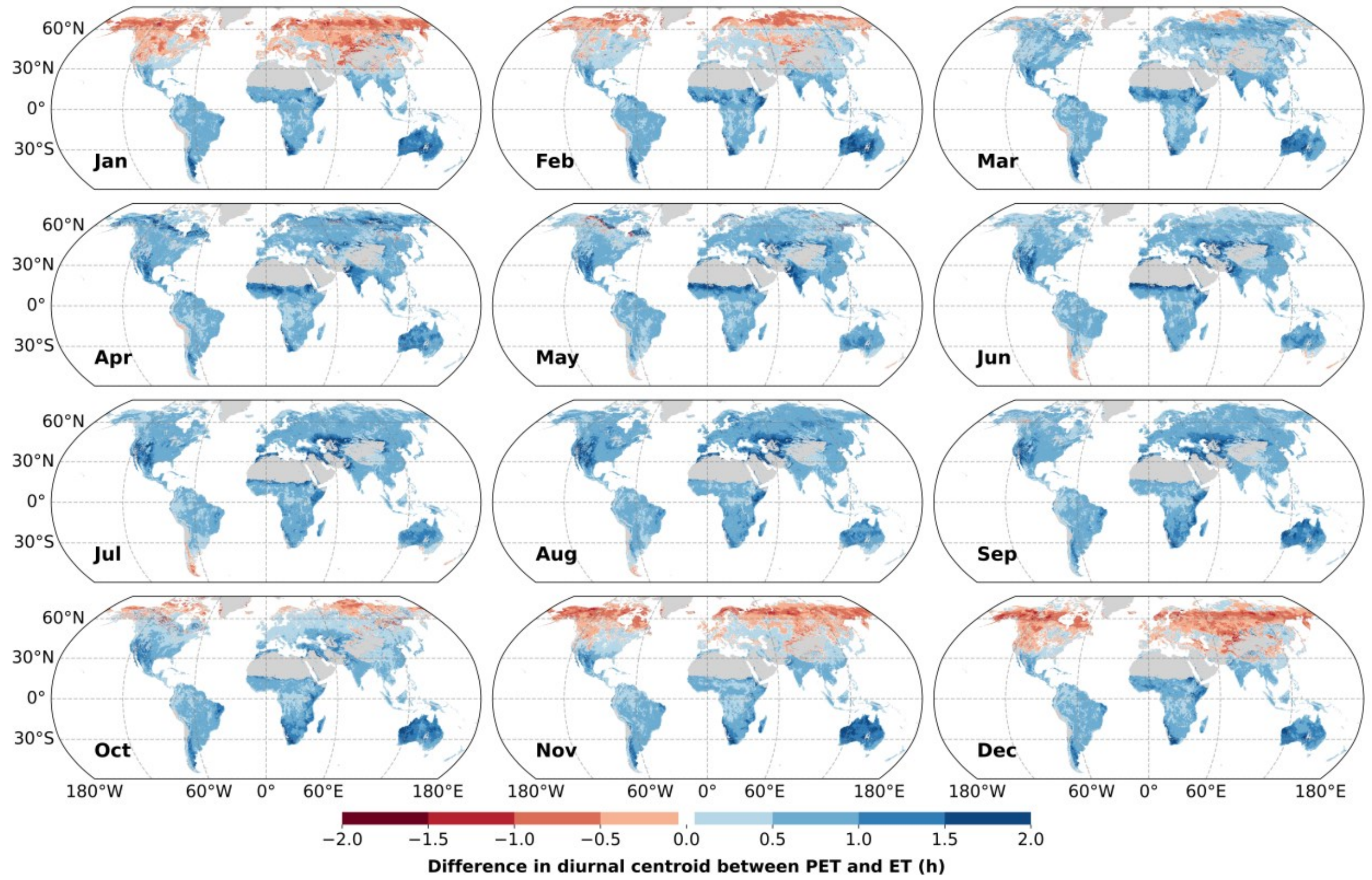

Fig. S6 Global patterns of the difference between diurnal centroid for PET (C_{PET}) and that for ET (C_{ET}) in 2020 ($C_{PET} - C_{ET}$).

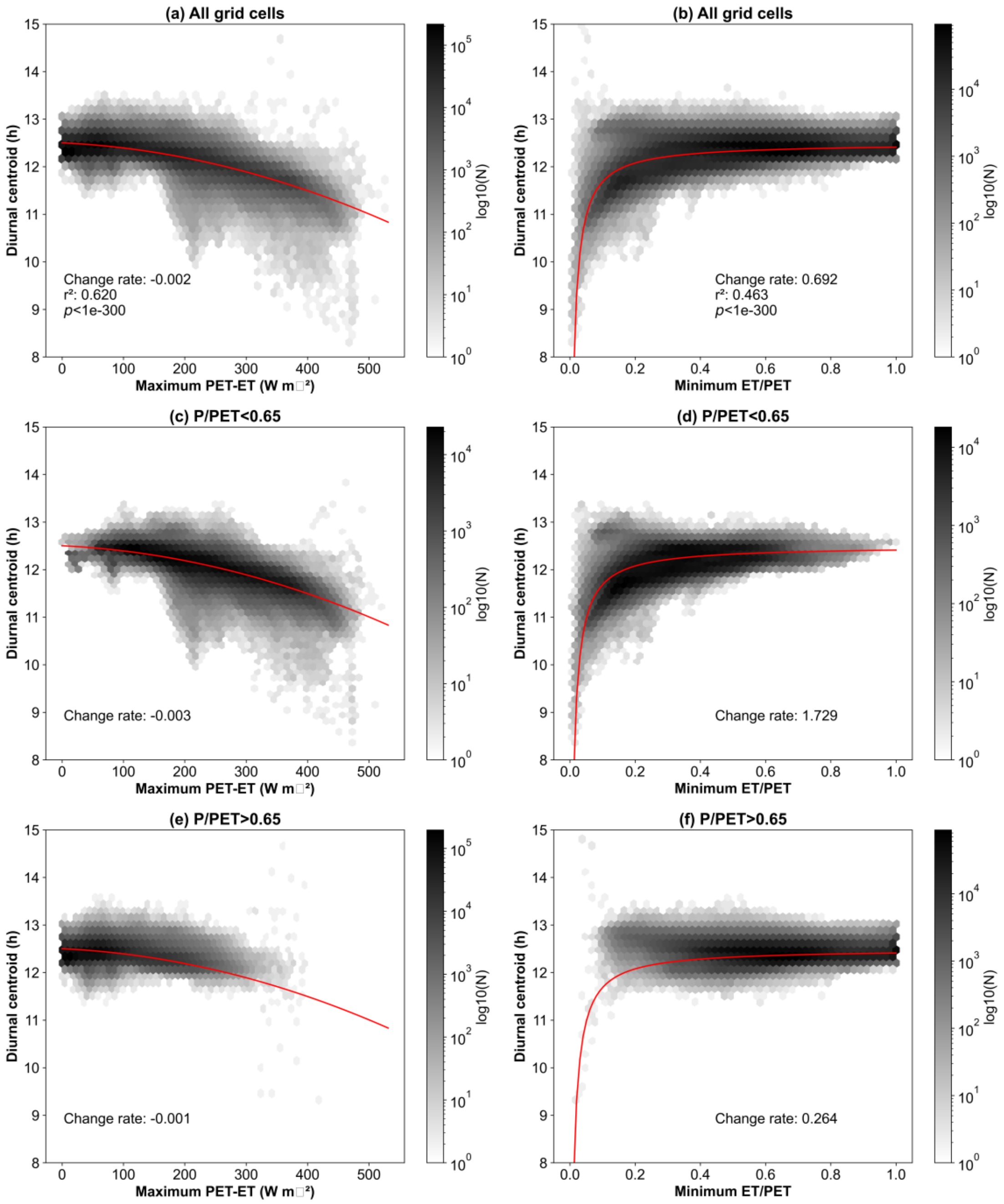

Fig. S7 The scatter plots of the yearly average of diurnal centroid for ET (C_{ET}) against discrepancies between hourly ET and hourly PET, (a, c, e) maximum PET-ET and (b, d, f) minimum ET/PET. Fitting curves were obtained based on all the data (maximum PET-ET: $y=-4.77\times10^{-6}x^2-6.07\times10^{-4}x+12.50$, minimum ET/PET: $y=12.50x/(0.007+x)$). The change rate is the mean rate of change of C_{ET} with respect to PET-ET or ET/PET, obtained from the derivative function of fitting curves in all data or subsets. C_{ET} has a greater rate of change with respect to (c) PET-ET and (d) ET/PET in arid areas (P/PET<0.65) than that (e, f) in humid areas (P/PET>0.65).

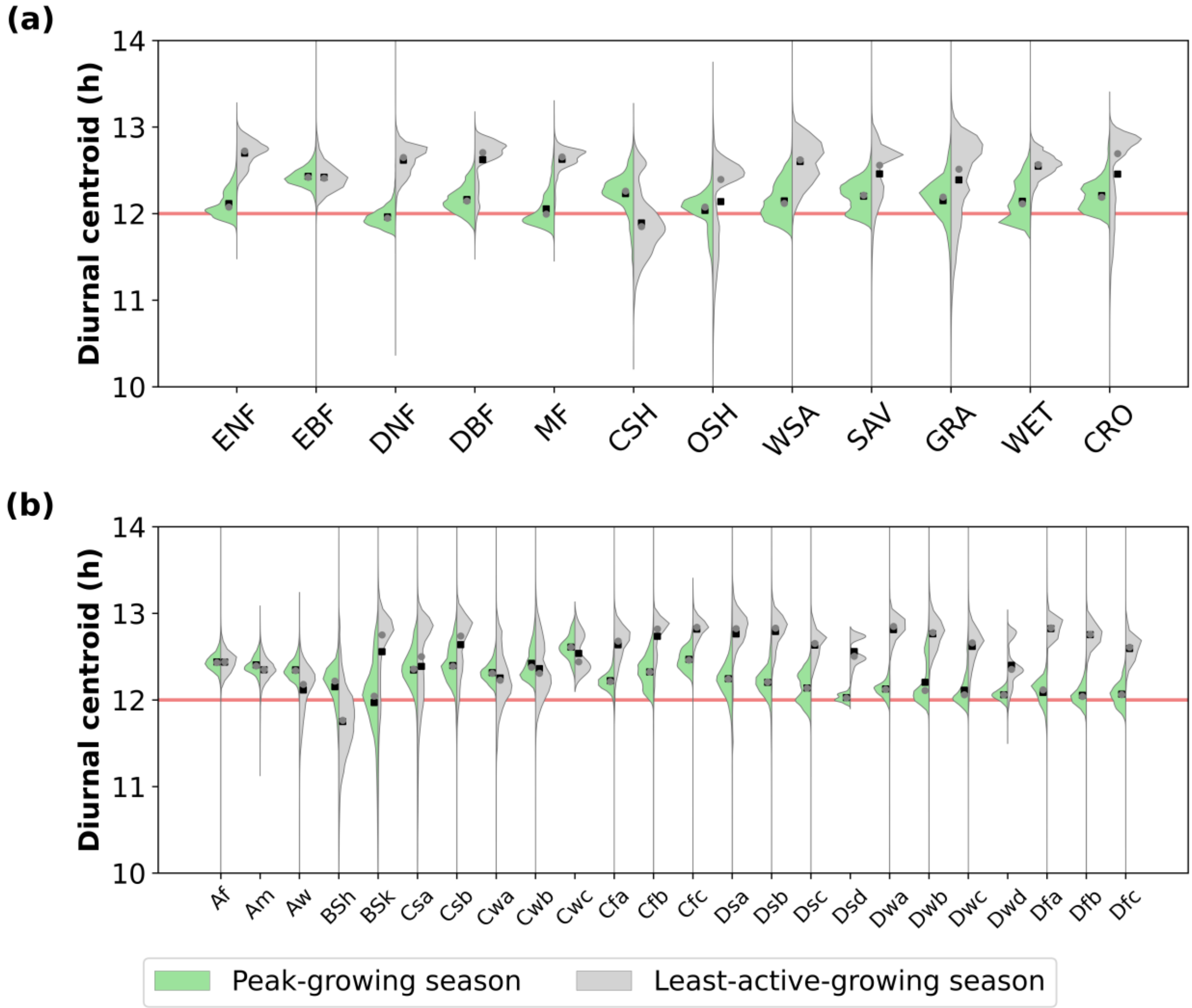

Fig. S8 Convergence and divergence in diurnal variation of ET across Plant Functional Types (PFTs) in different climate zones, and mean (black rectangles) and median (grey circles) values are also reported, (a) PFTs, ENF: Evergreen Needleleaf Forest, EBF: Evergreen Broadleaf Forest, DNF: Deciduous Needleleaf Forest, DBF: Deciduous Broadleaf Forest, MF: Mixed Forests, CSH: Closed Shrublands, OSH: Open Shrublands, WSA: Woody Savannas, SAV: Savannas, GRA: Grasslands, WET: Permanent Wetlands, CRO: Croplands, (b)Koppen climate categories, Af: Tropical Rainforest, Am: Tropical Monsoon, Aw: Tropical Savanna, BSh: Hot Semi-Arid, BSk: Cold Semi-Arid, Csa: Mediterranean, Hot Summer, Csb: Mediterranean, Warm Summer, Cwa: Subtropical, Dry Winter, Hot Summer, Cwb: Subtropical, Dry Winter, Warm Summer, Cwc: Subtropical, Dry Winter, Cold Summer, Cfa: Subtropical, Humid, Hot Summer, Cfb: Subtropical, Humid, Warm Summer, Cfc: Subtropical, Humid, Cold Summer, Dsa: Continental, Dry Summer, Hot Summer, Dsb: Continental, Dry Summer, Warm Summer, Dsc: Continental, Dry Summer, Cold Summer, Dsd: Continental, Dry Summer, Very Cold Winter, Dwa: Continental, Dry Winter, Hot Summer, Dwb: Continental, Dry Winter, Warm Summer, Dwc: Continental, Dry Winter, Cold Summer, Dwd: Continental, Dry Winter, Very Cold Winter, Dfa: Continental, Humid, Hot Summer, Dfb: Continental, Humid, Warm Summer, Dfc: Continental, Humid, Cold Summer.

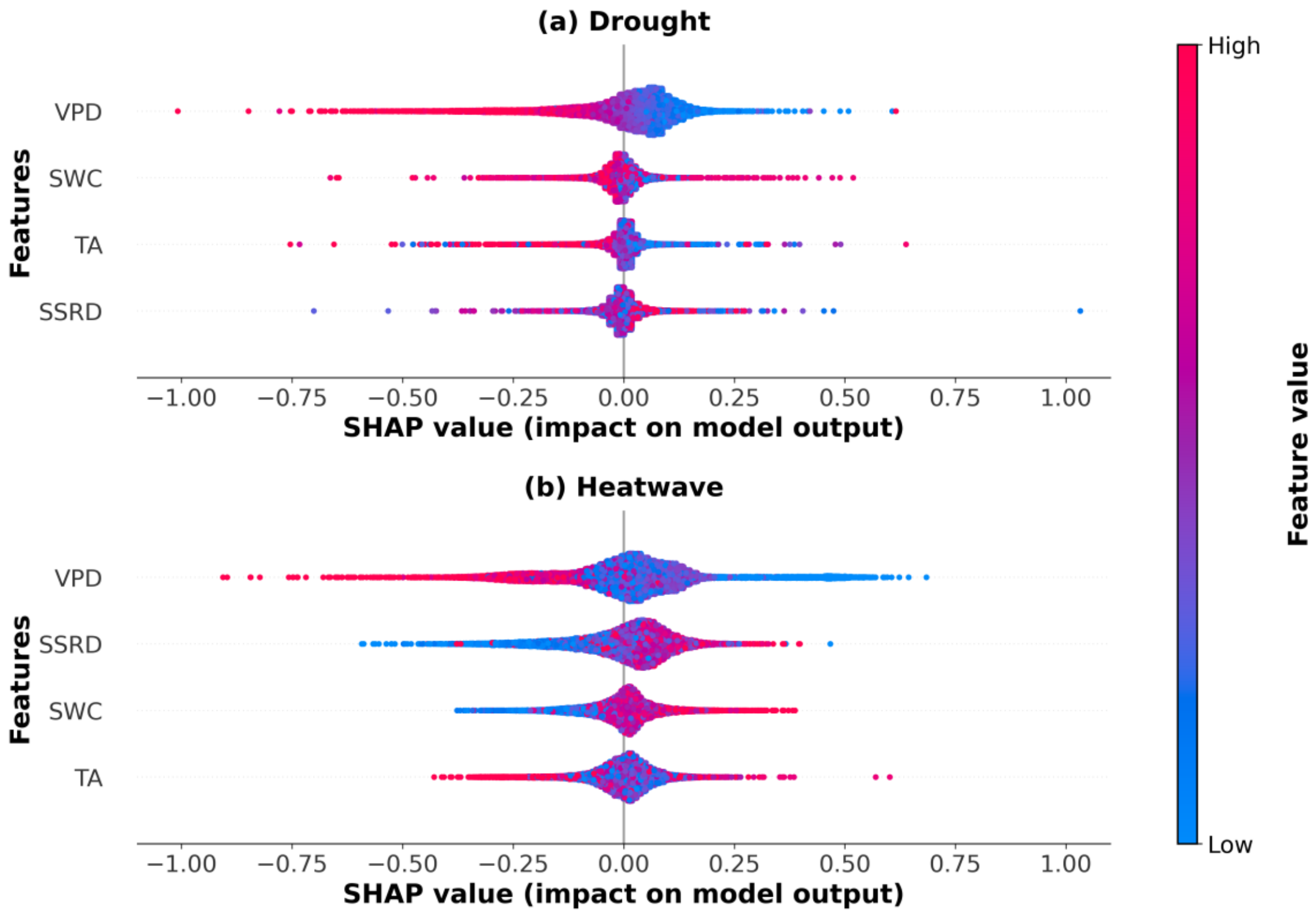

Fig. S9 SHAP-based attribution of vapor pressure deficit (VPD), soil water content (SWC), air temperature (TA), and downward shortwave radiation (SSRD) under (a) drought and (b) heatwave conditions.

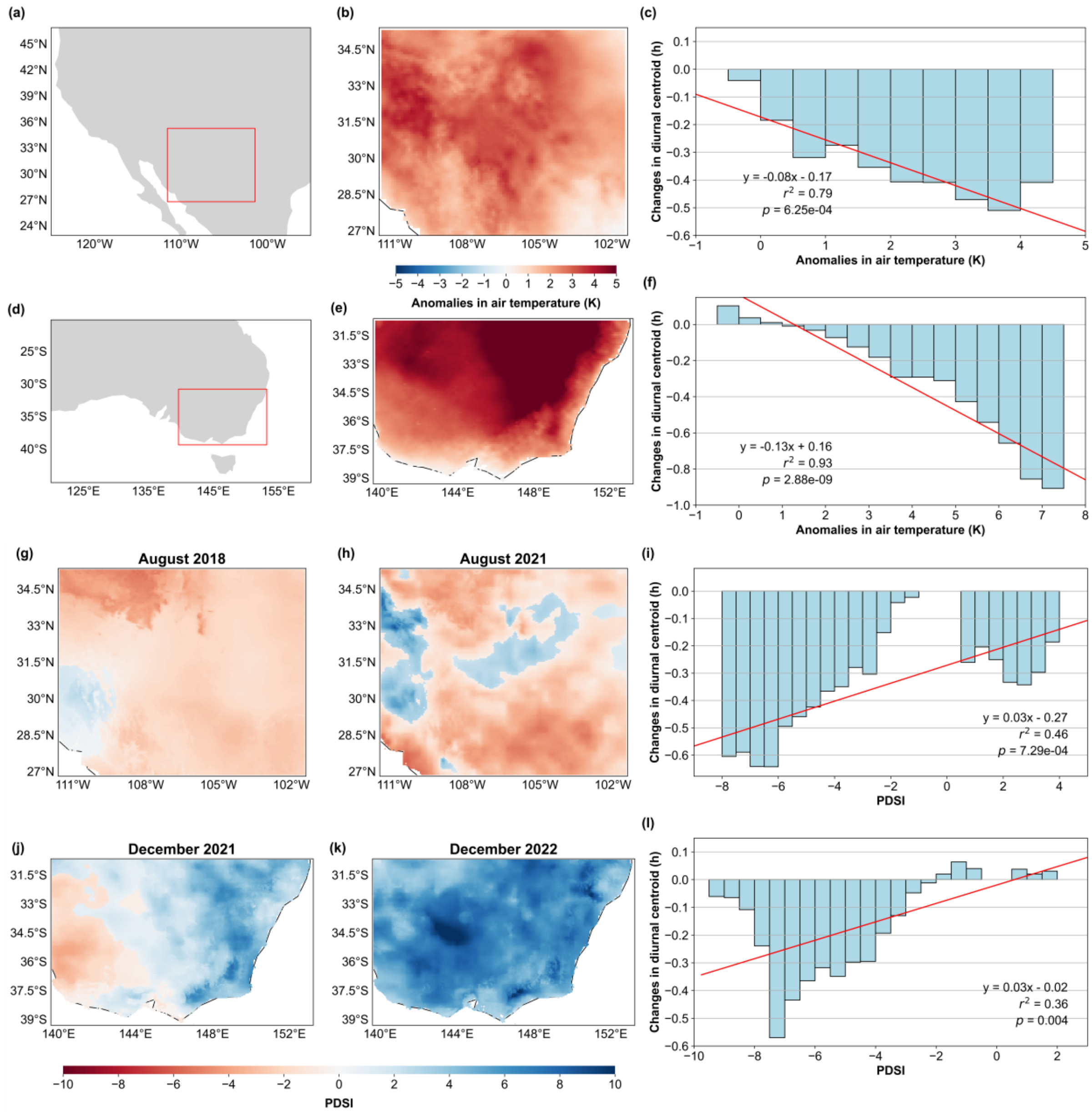

Fig. S10 Effects of drought and heatwave compound events on diurnal variations of ET in (a, b, c, g, h, i) southwestern North America and (d, e, f, j, k, l) southern Australia. (a, d) Location of study domain. (b, e) The anomalies in air temperature (Ta) map, represented by the difference between the Ta in a drought/heatwave year and the average Ta in normal years. (c, f) Average changes in C_{ET} response to Ta intervals. We divided the Ta into intervals of 0.5 K and then calculated the average value of changes in C_{ET} for each interval. The changes in C_{ET} and Ta generally showed a negative correlation, as indicated by the fitting line (red line). (g, h, j, k) Palmer Drought Severity Index (PDSI) during the normal years. (i, l) Average changes in C_{ET} response to PDSI intervals. We divided the PDSI into intervals of 0.5 and then calculated the average value of changes in diurnal centroid for each interval. The changes in diurnal centroid and PDSI generally showed a positive correlation, as indicated by the fitting line (red line).

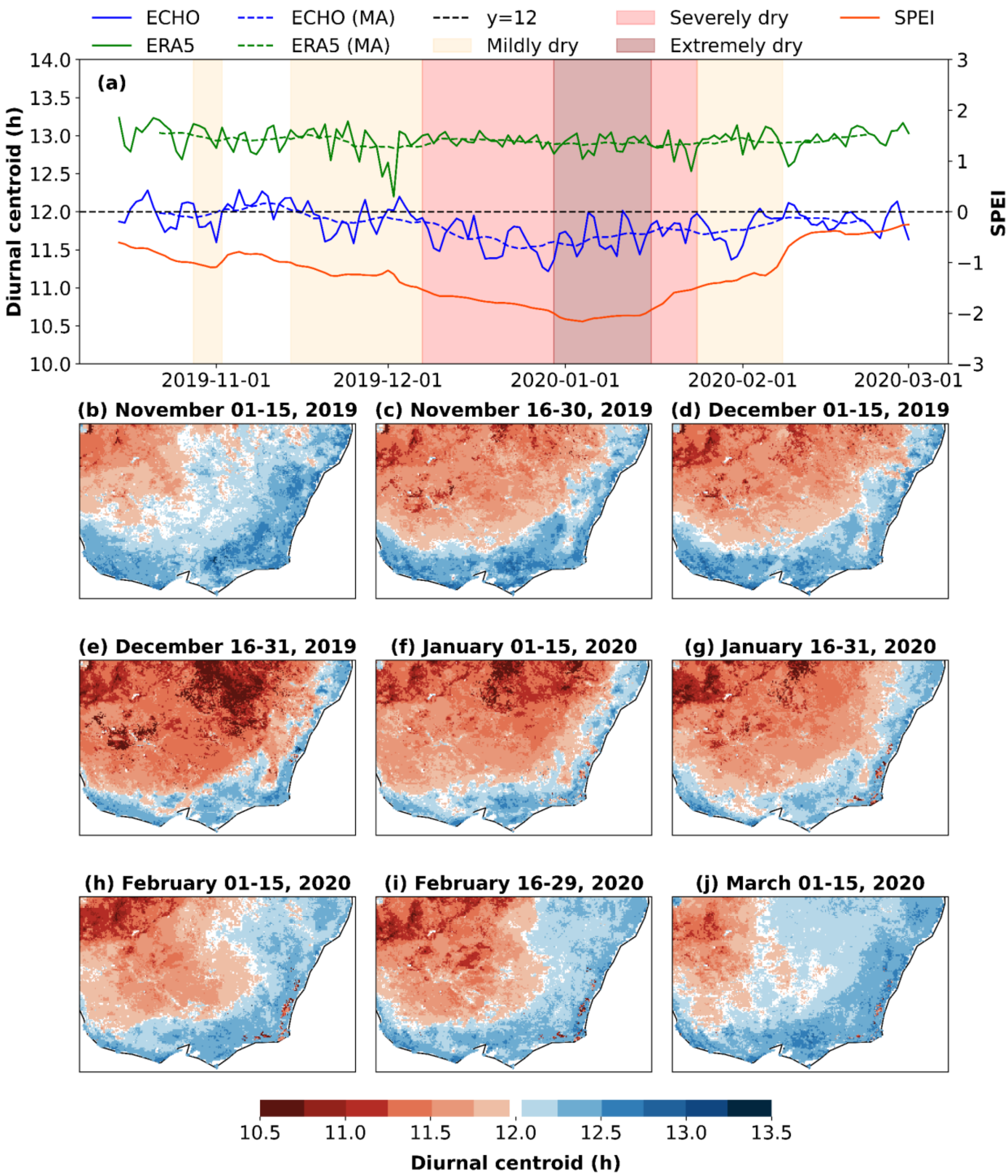

Fig. S11 Evolution of daily diurnal centroid in ET (C_{ET}) dynamics and standardized precipitation-evapotranspiration index (SPEI) trends in southern Australia during drought and heatwave compound events in 2019-2020. (a) Time series of daily average C_{ET} from ECHO-ET and ERA5-Land, along with SPEI values, from October 2019 to March 2020, illustrating the relationship between C_{ET} morning shifts and drought severity (indicated by SPEI and shaded regions for "mildly dry", "severely dry", and "extremely dry" periods). (b-j) Spatial distribution of C_{ET} across the study area during different 15-day periods from November (pre-drought) in 2019 to March (post-drought) in 2020.

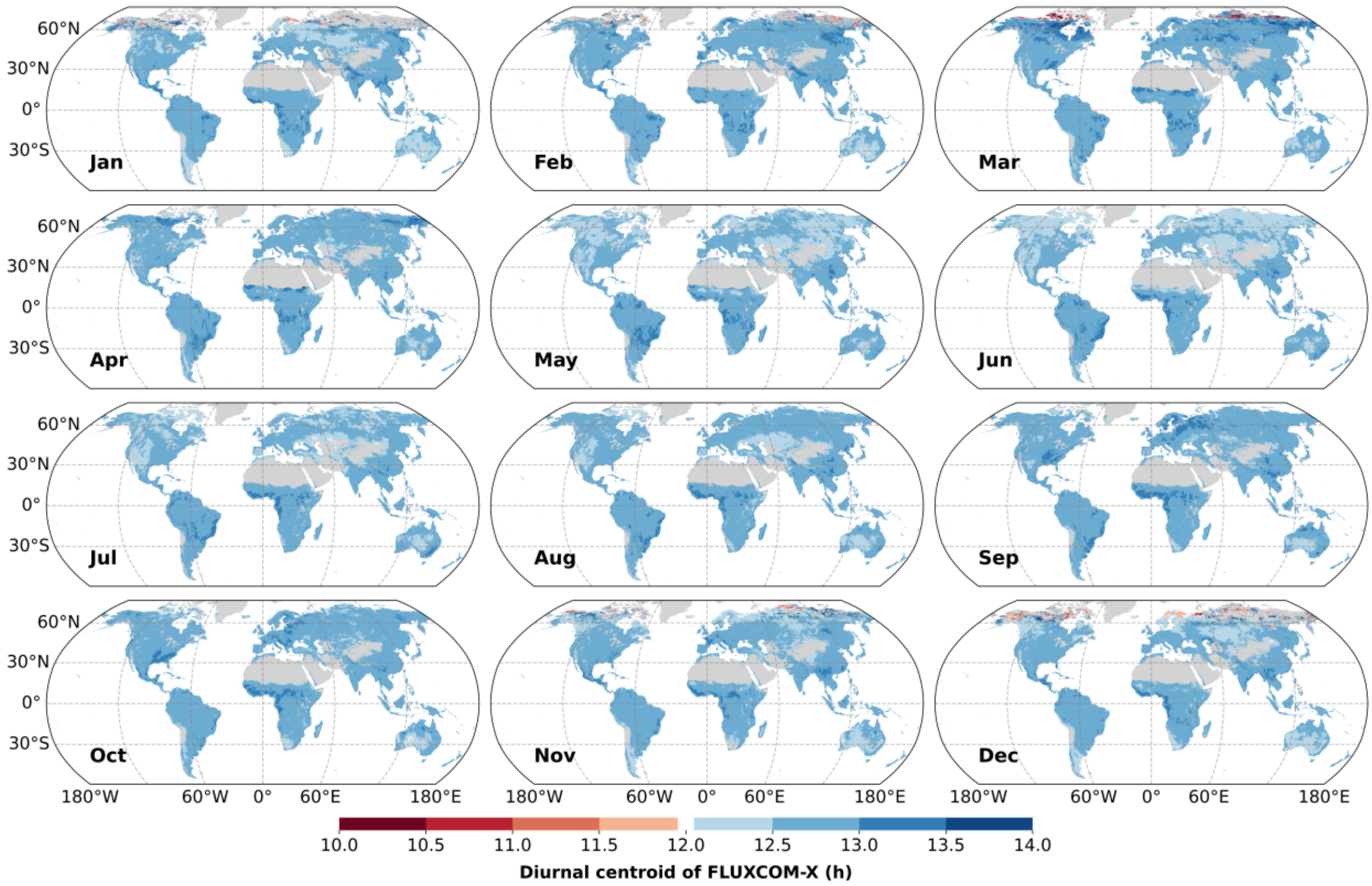

Fig. S12 Global patterns of the diurnal centroid for ET (C_{ET}) (6-18 h local solar time) in 2020, derived from FLUXCOM-X hourly dataset.

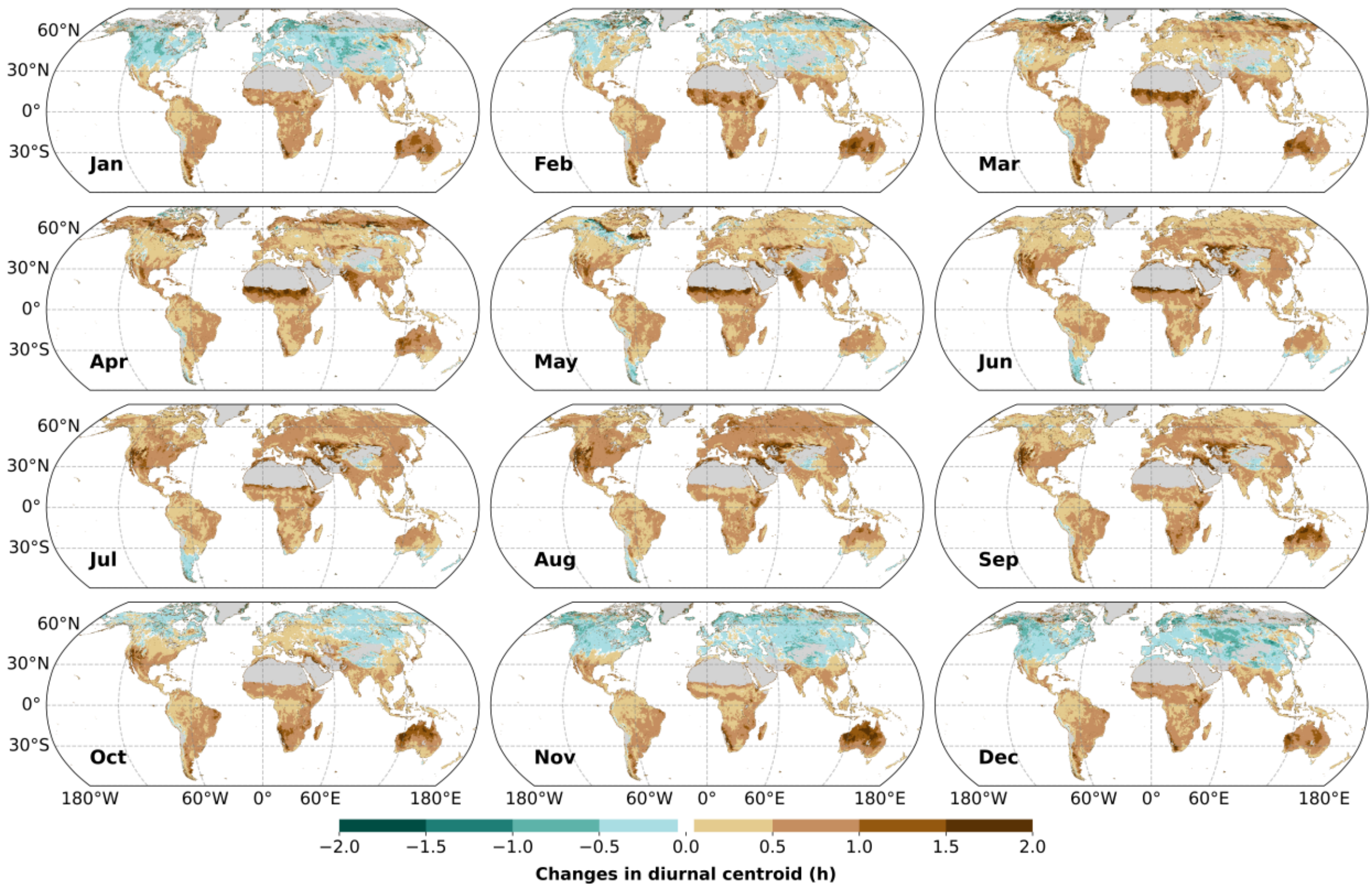

Fig. S13 Difference in diurnal centroid for ET between FLUXCOM-X and our ECHO-ET dataset (the diurnal centroid of FLUXCOM-X minus that of ECHO-ET).

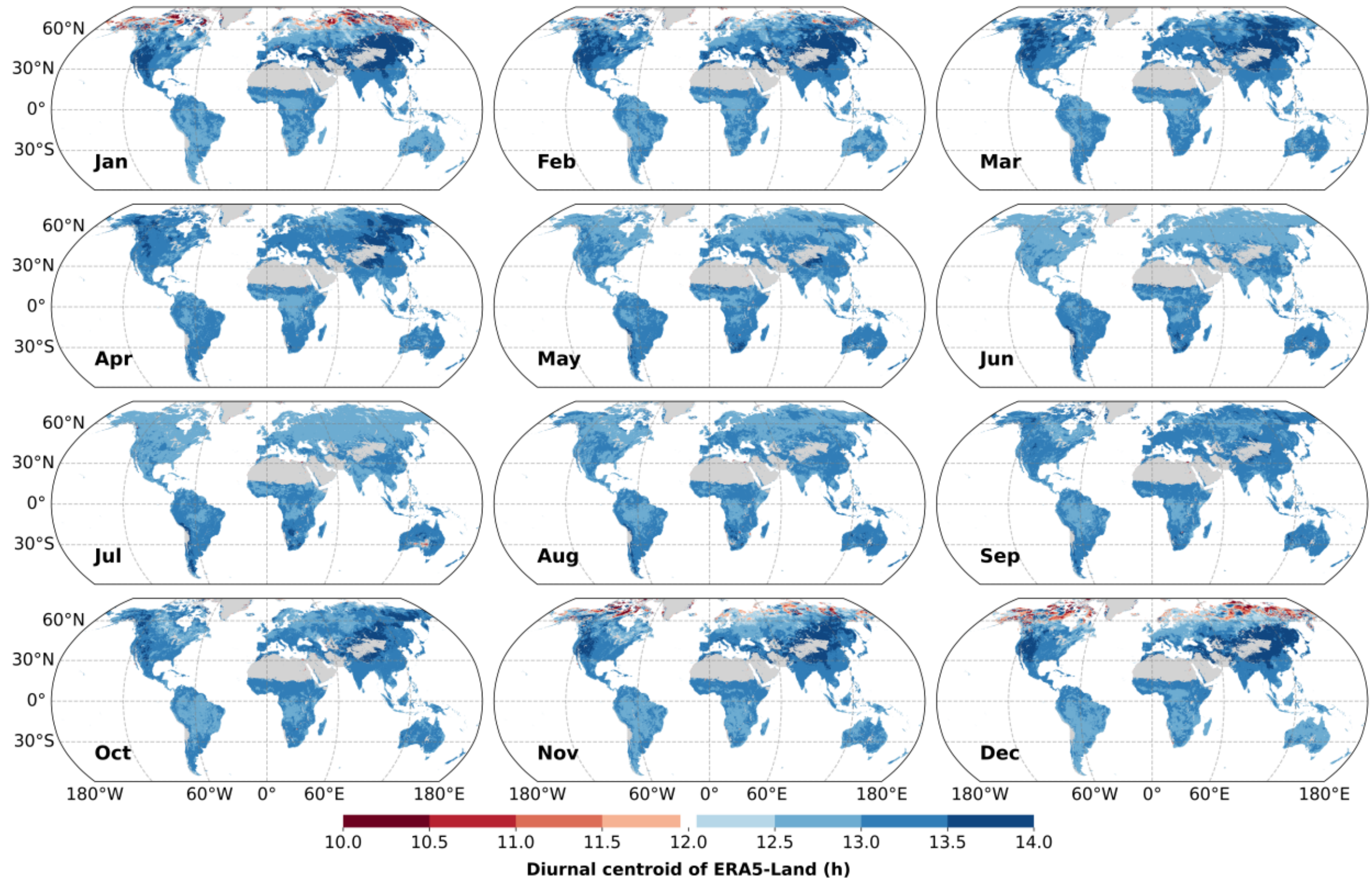

Fig. S14 Global patterns of the diurnal centroid for ET (C_{ET}) (6-18 h local solar time) in 2020, derived from ERA5-Land hourly dataset.

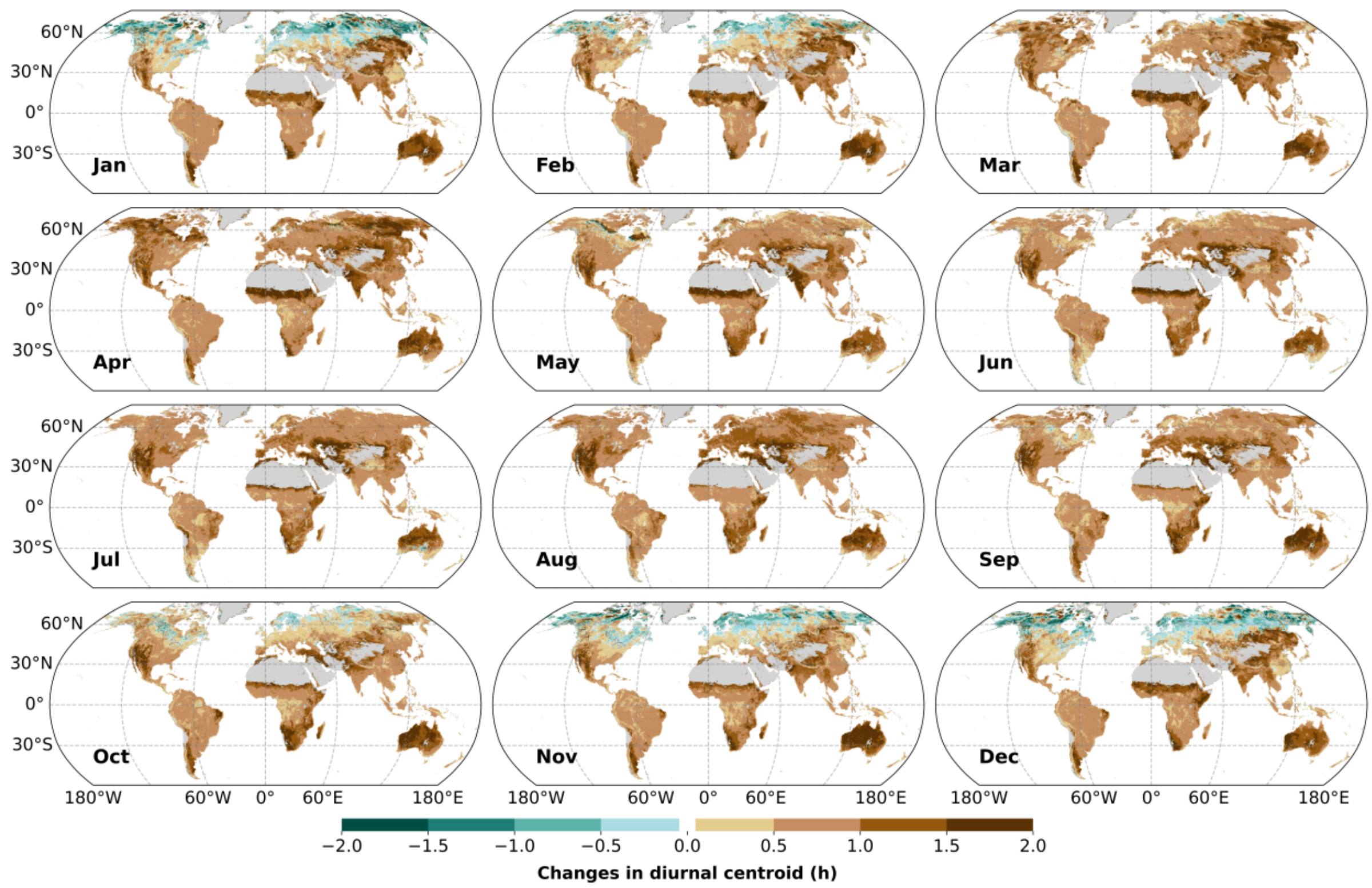

Fig. S15 Difference in diurnal centroid for ET between ERA5-Land Hourly and our ECHO-ET dataset (the diurnal centroid of ERA5-Land Hourly minus that of ECHO-ET).

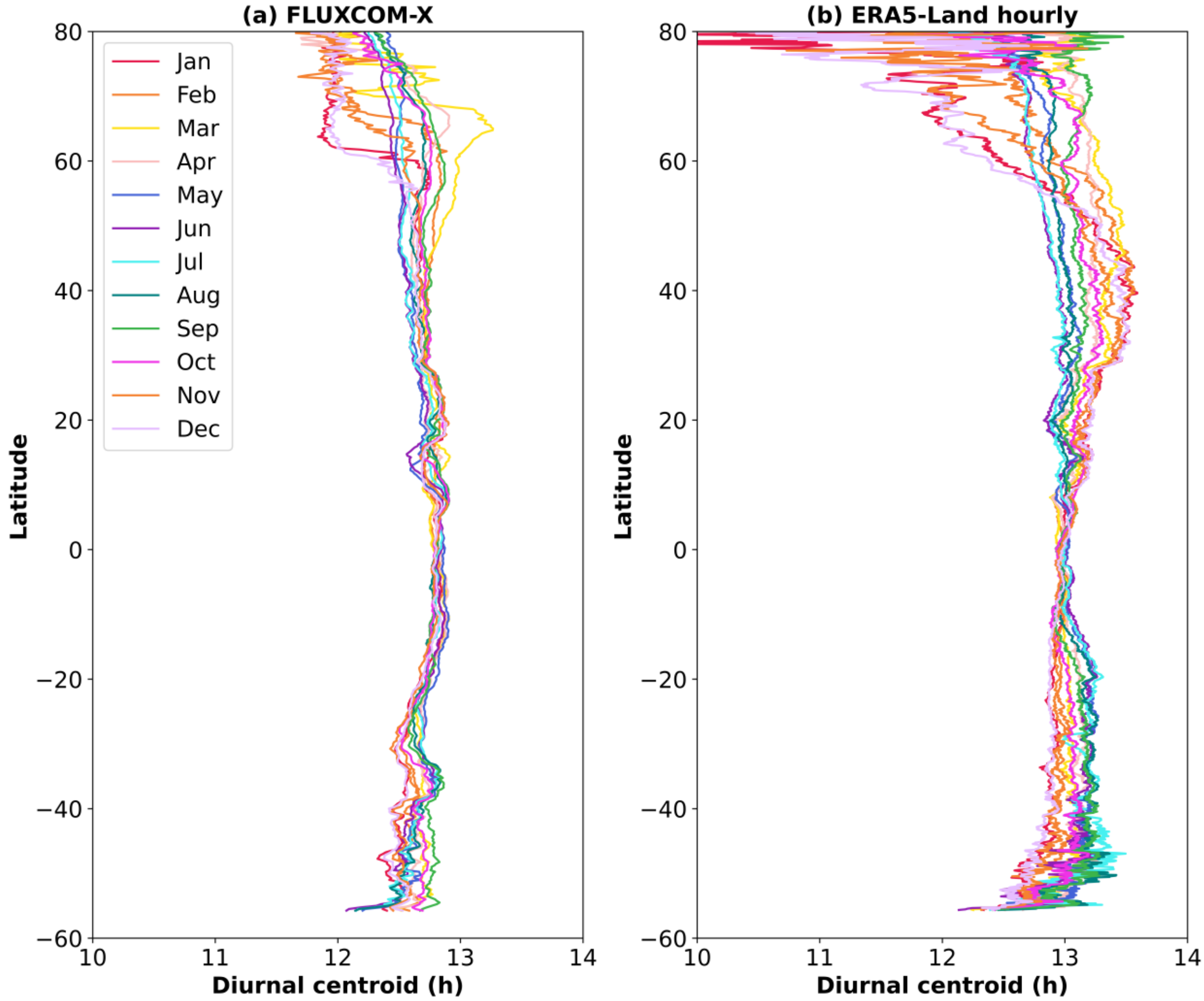

Fig. S16 The latitudinal profile of monthly diurnal centroid for ET from (a) FLUXCOM-X and (b) ERA5-Land hourly.

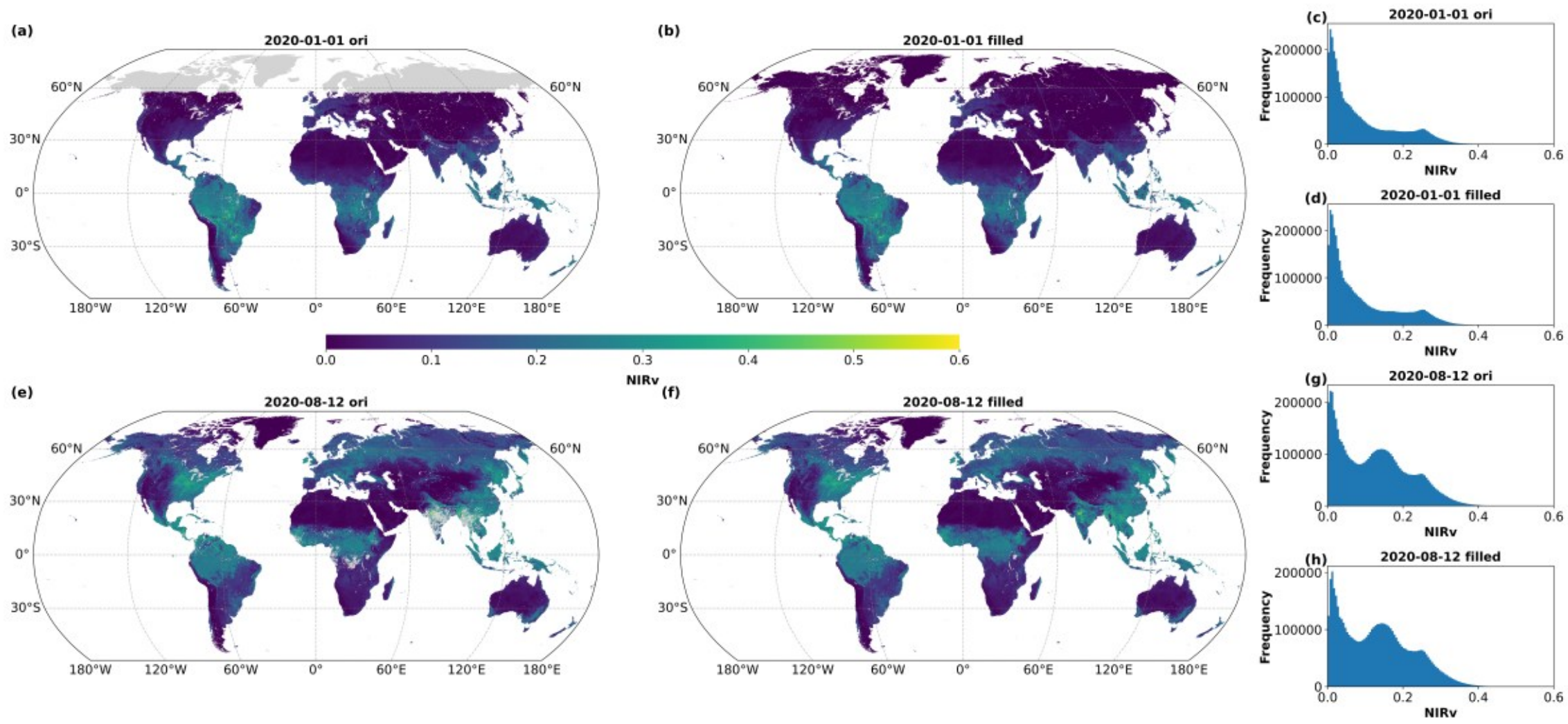

Fig. S17 Two examples of NIRv processing on 2020-01-01 and 2020-8-12. (a) and (e) maps before filling the gaps, (b) and (f) maps after filling the gaps, (c) and (g) frequency distribution charts before filling the gaps, (d) and (h) frequency distribution charts after filling the gaps.

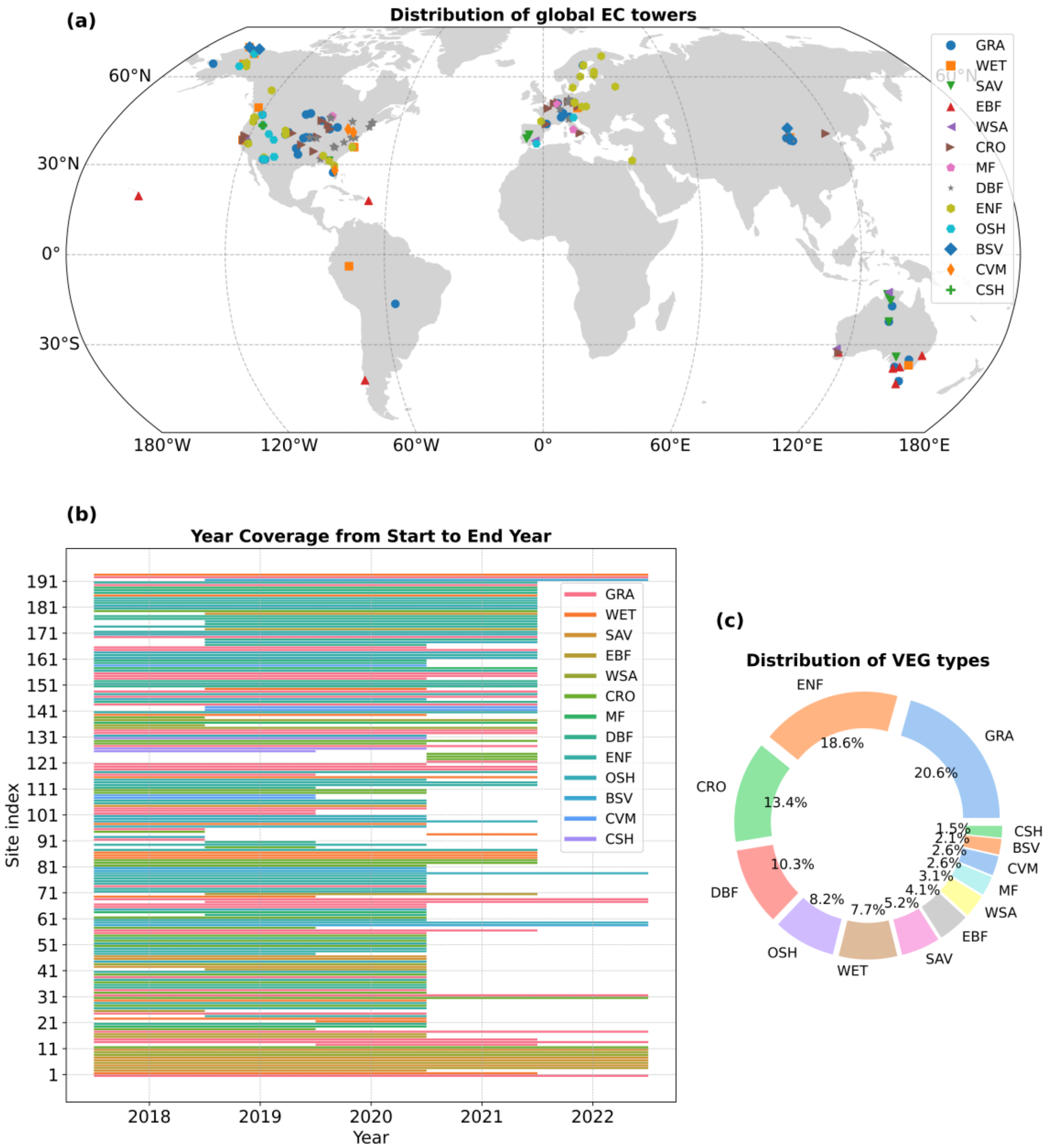

Fig. S18 (a) Distribution of eddy covariance sites, (b) Year coverage, (c) IGBP land cover types of eddy covariance sites.

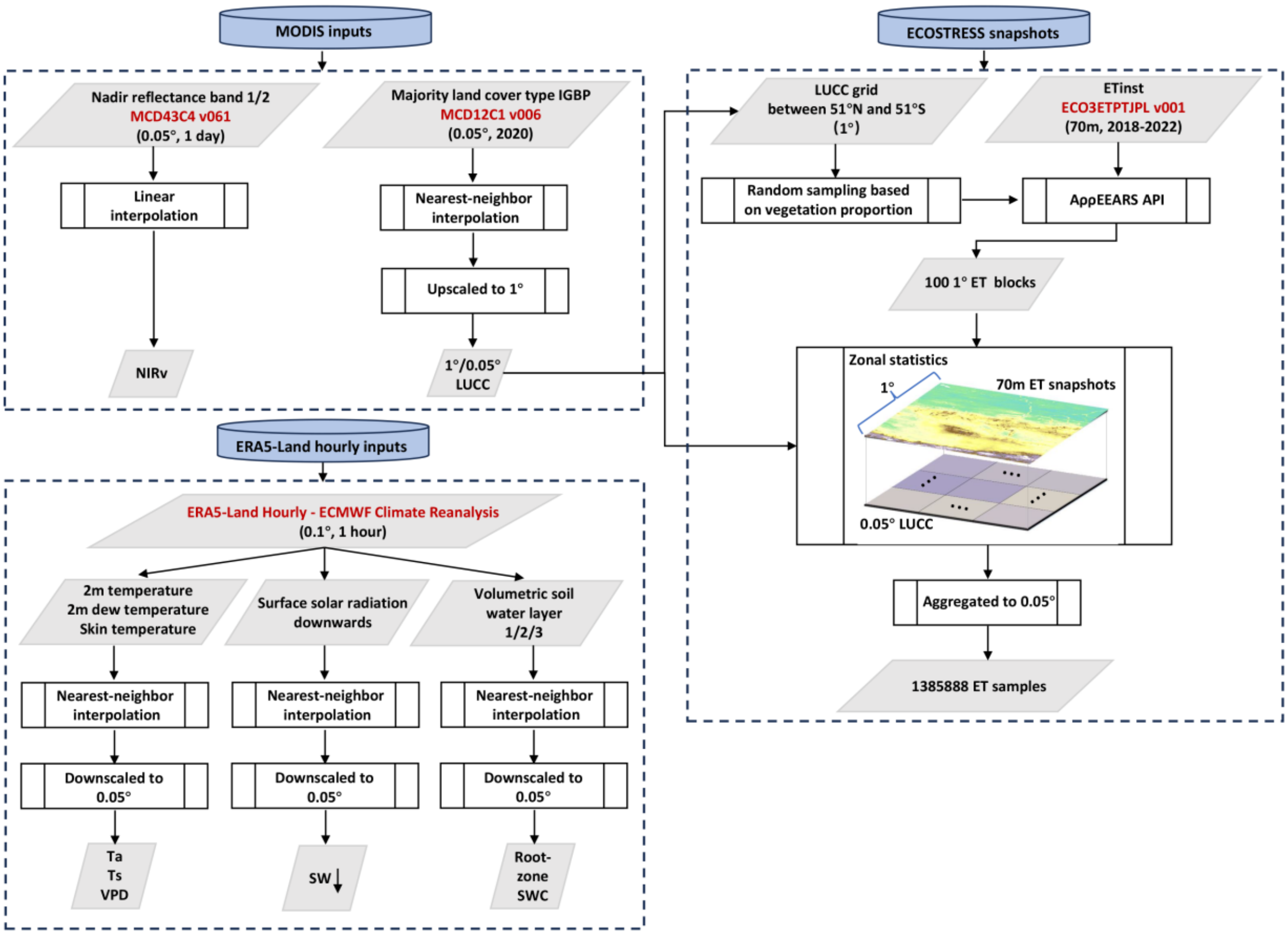

Fig. S19 Flowchart for the processing of the ECOSTRESS training dataset.

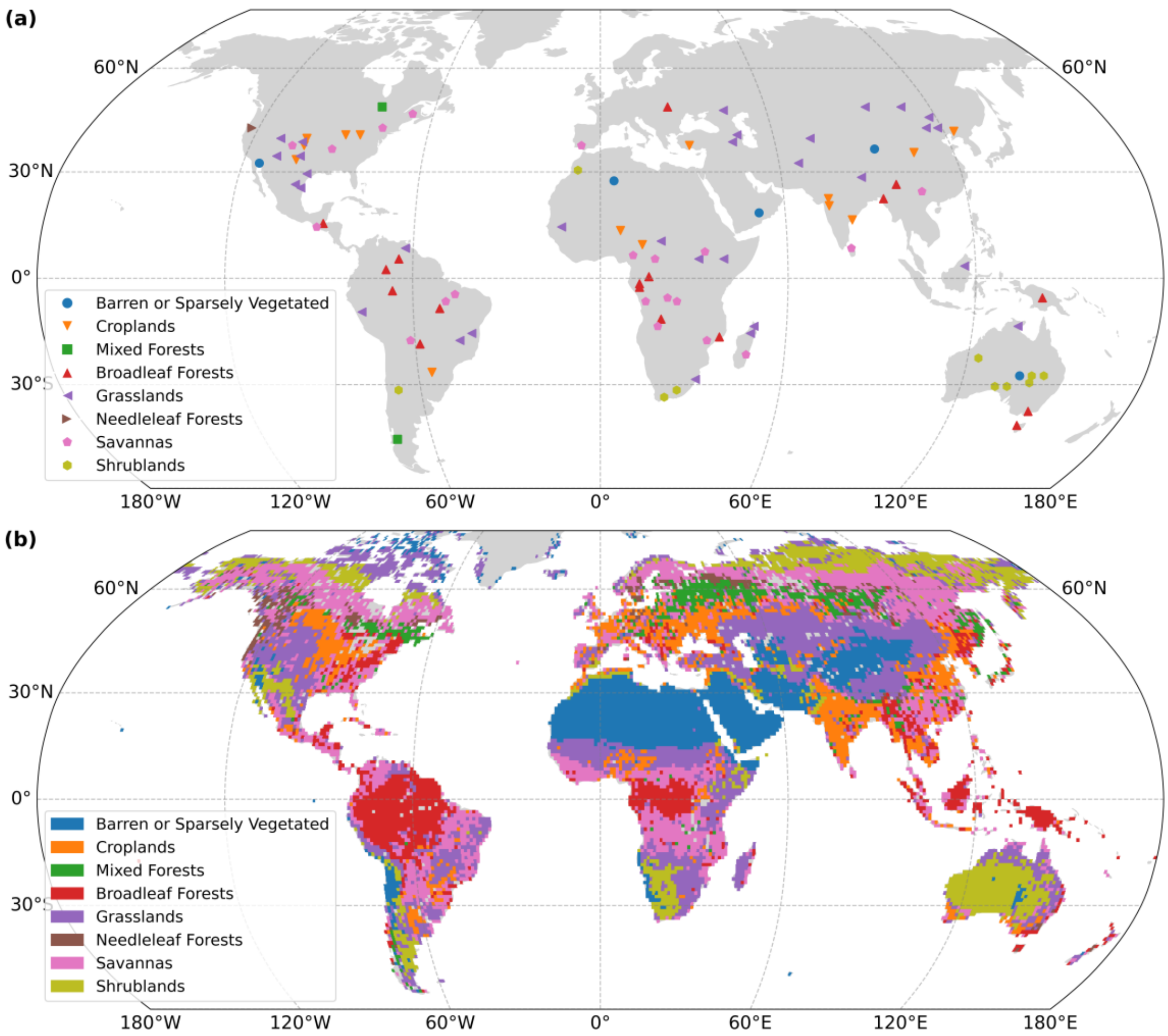

Fig. S20 (a) Distribution of 100 selected blocks (1°× 1°), (b) MODIS land cover map, resampled to 1°× 1°.

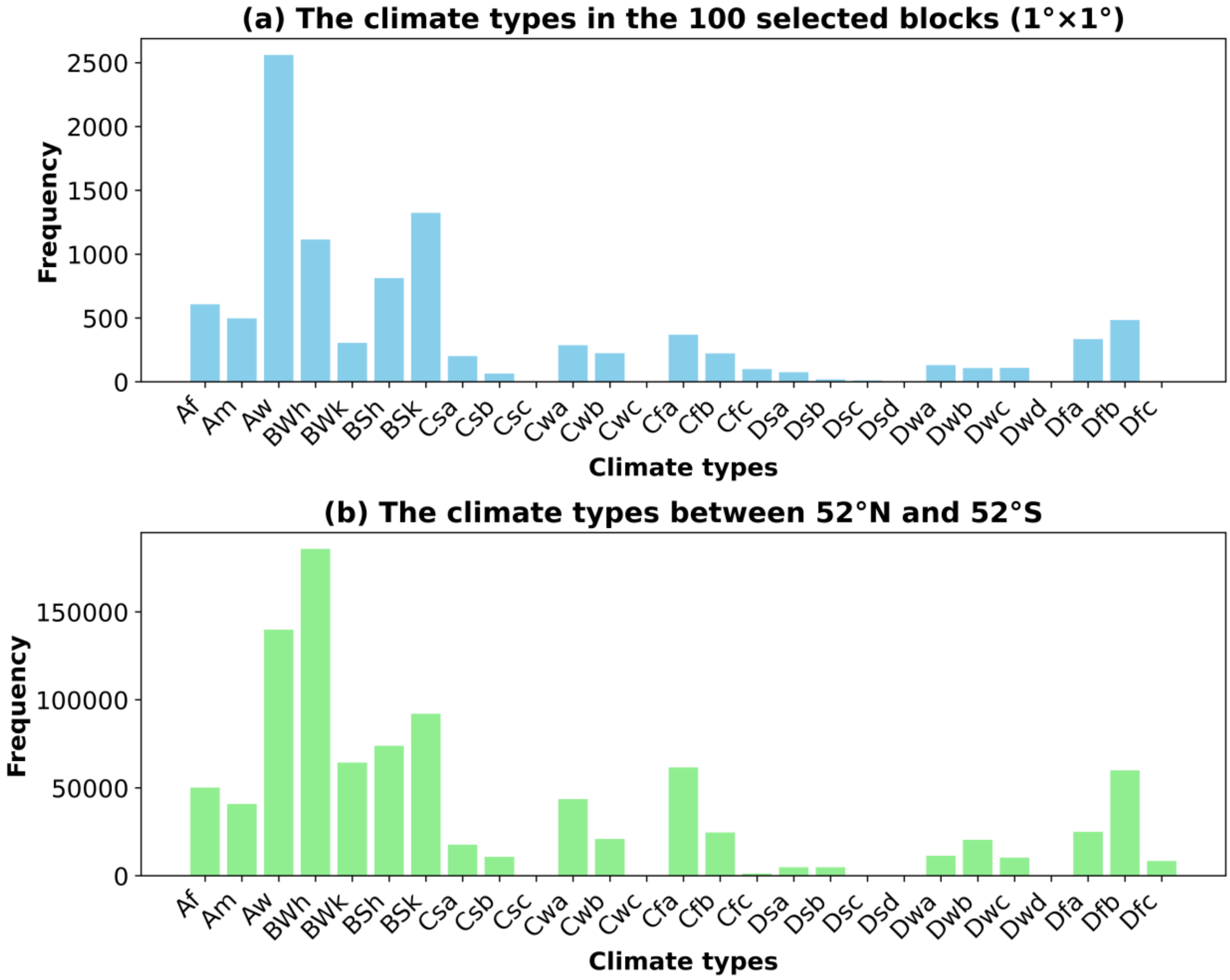

Fig. S21 Consistency of climate types between (a) the selected blocks and (b) ECOSTRESS coverage area. Af: Tropical Rainforest, Am: Tropical Monsoon, Aw: Tropical Savanna, BSh: Hot Semi-Arid, BSk: Cold Semi-Arid, Csa: Mediterranean, Hot Summer, Csb: Mediterranean, Warm Summer, Cwa: Subtropical, Dry Winter, Hot Summer, Cwb: Subtropical, Dry Winter, Warm Summer, Cwc: Subtropical, Dry Winter, Cold Summer, Cfa: Subtropical, Humid, Hot Summer, Cfb: Subtropical, Humid, Warm Summer, Cfc: Subtropical, Humid, Cold Summer, Dsa: Continental, Dry Summer, Hot Summer, Dsb: Continental, Dry Summer, Warm Summer, Dsc: Continental, Dry Summer, Cold Summer, Dsd: Continental, Dry Summer, Very Cold Winter, Dwa: Continental, Dry Winter, Hot Summer, Dwb: Continental, Dry Winter, Warm Summer, Dwc: Continental, Dry Winter, Cold Summer, Dwd: Continental, Dry Winter, Very Cold Winter, Dfa: Continental, Humid, Hot Summer, Dfb: Continental, Humid, Warm Summer, Dfc: Continental, Humid, Cold Summer.

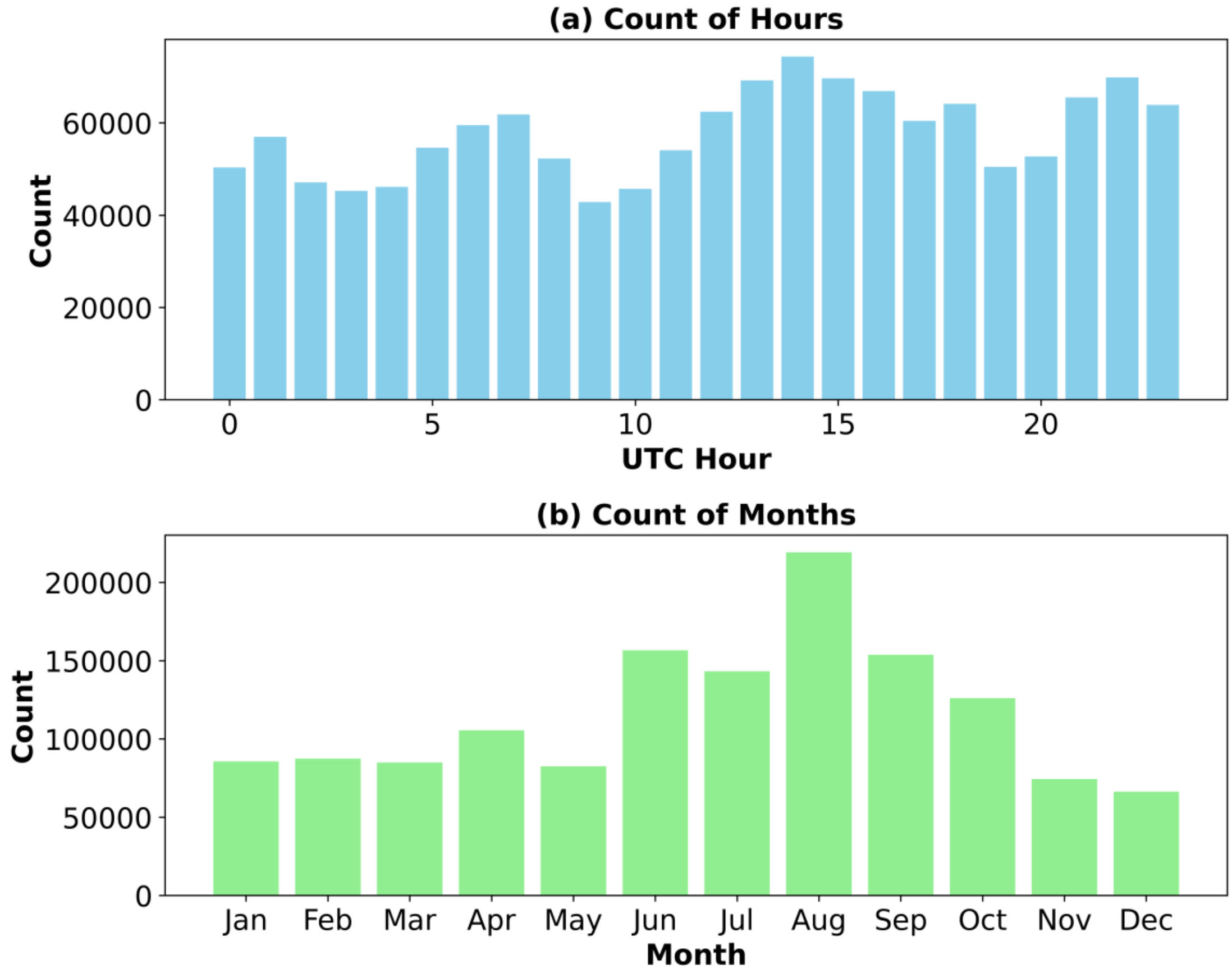

Fig. S22 Distribution of the 0.05° ECOSTRESS ET samples within (a) UTC hours and (b) months.

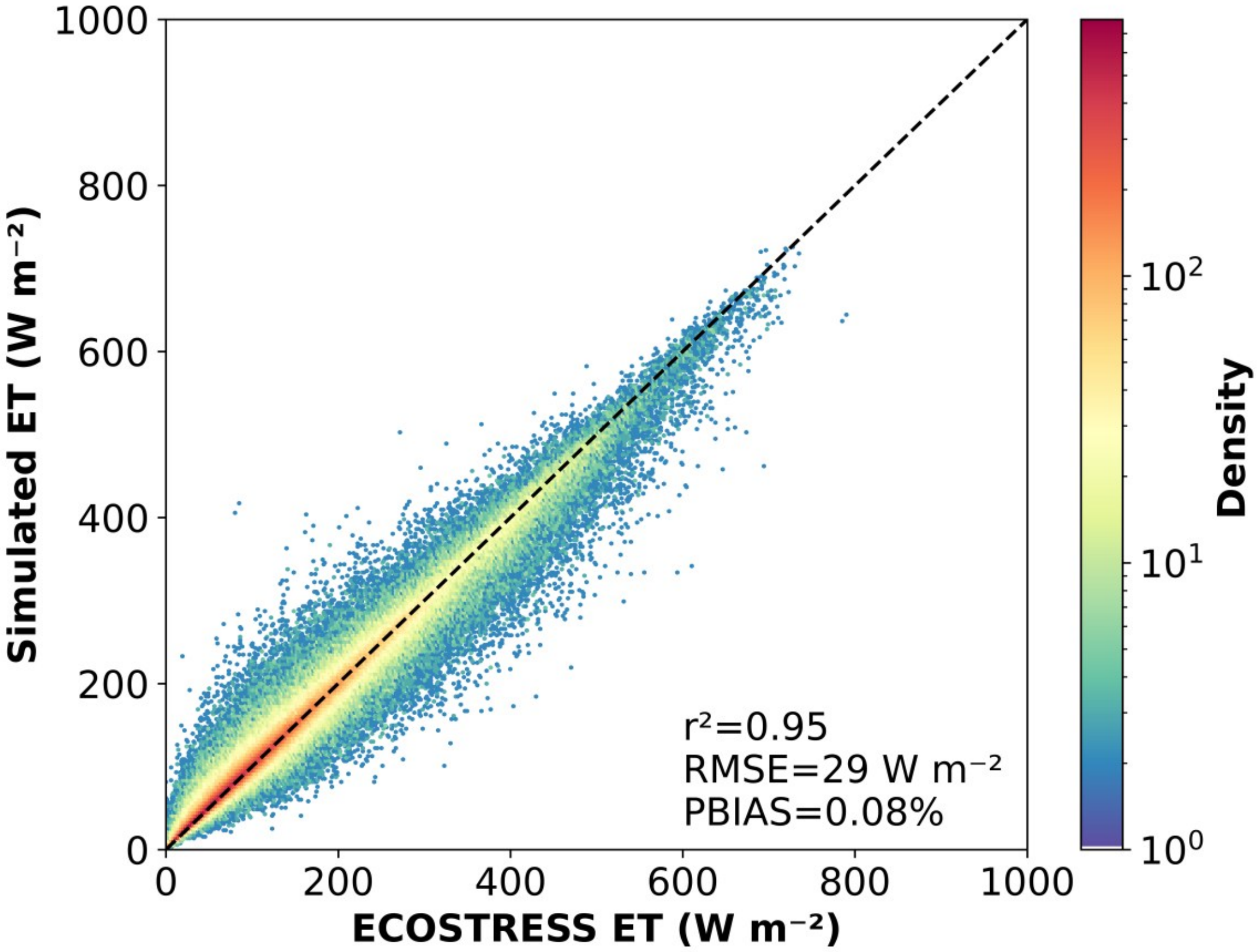

Fig. S23 The scatter plot of simulated ET by the Random Forest model against the original ECOSTRESS ET ($p < 1 \times 10^{-300}$, sample size = 415,773).

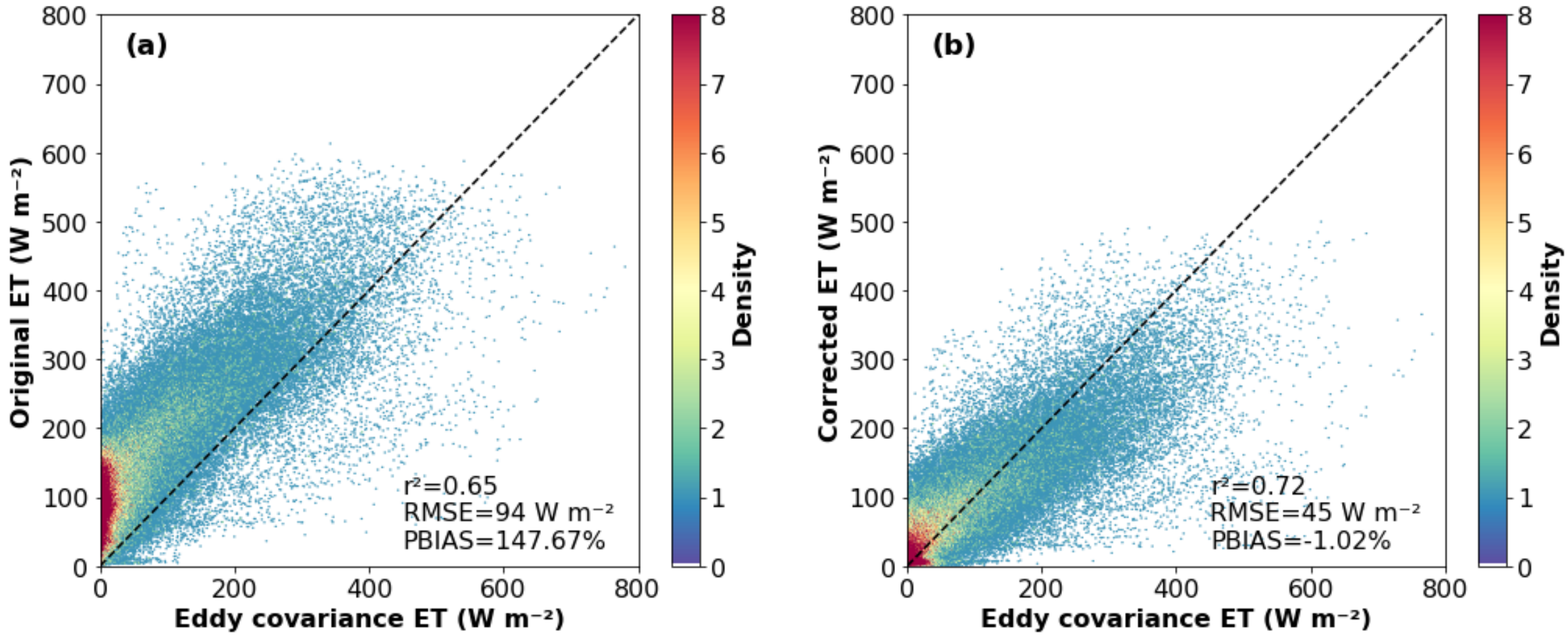

Fig. S24 The scatter plots of simulated ET against eddy covariance ET: (a) before correction ($p < 1 \times 10^{-300}$, sample size = 169,560) and (b) after correction ($p < 1 \times 10^{-300}$, sample size = 169,560).

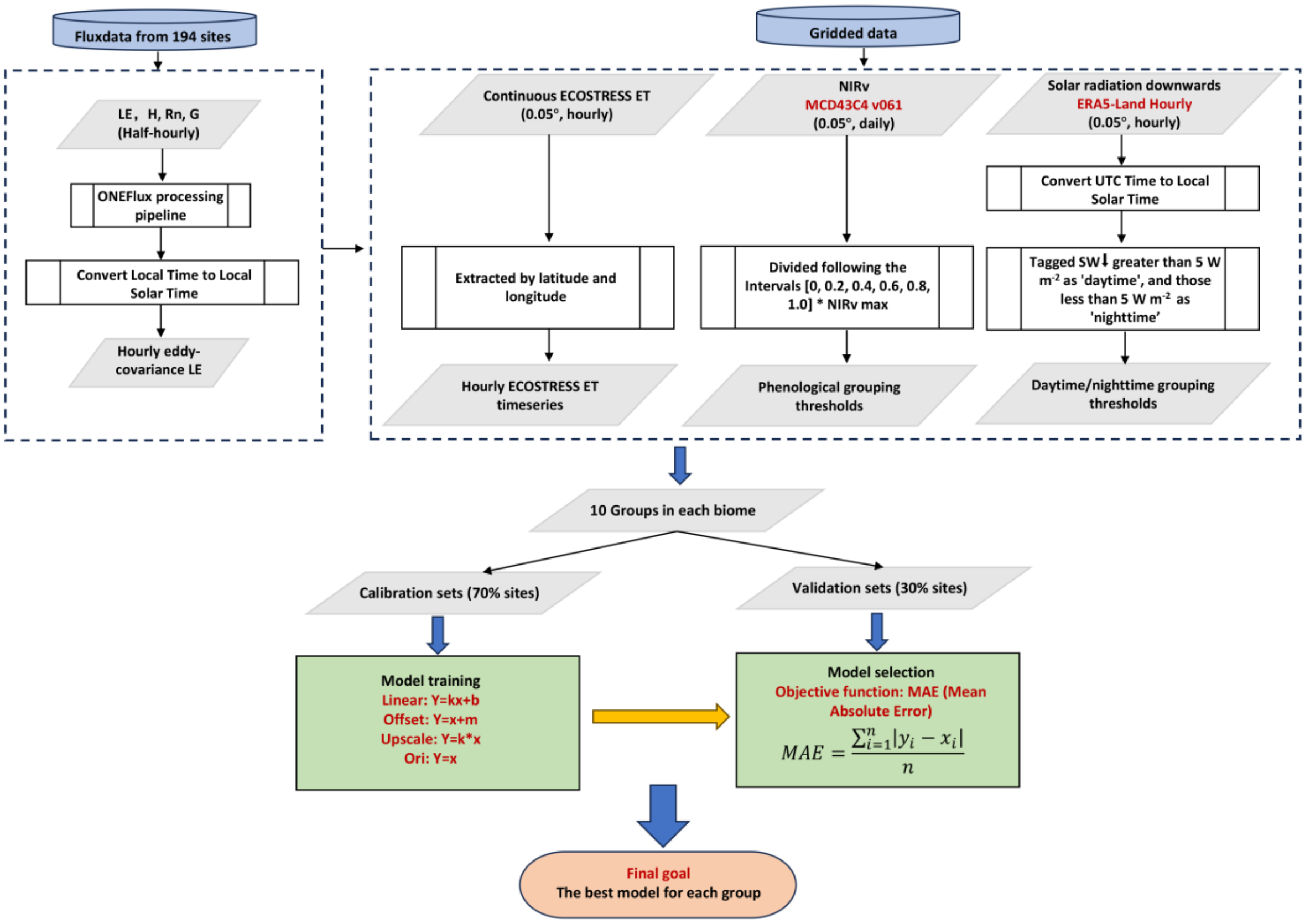

Fig. S25 Flowchart of ET correction.

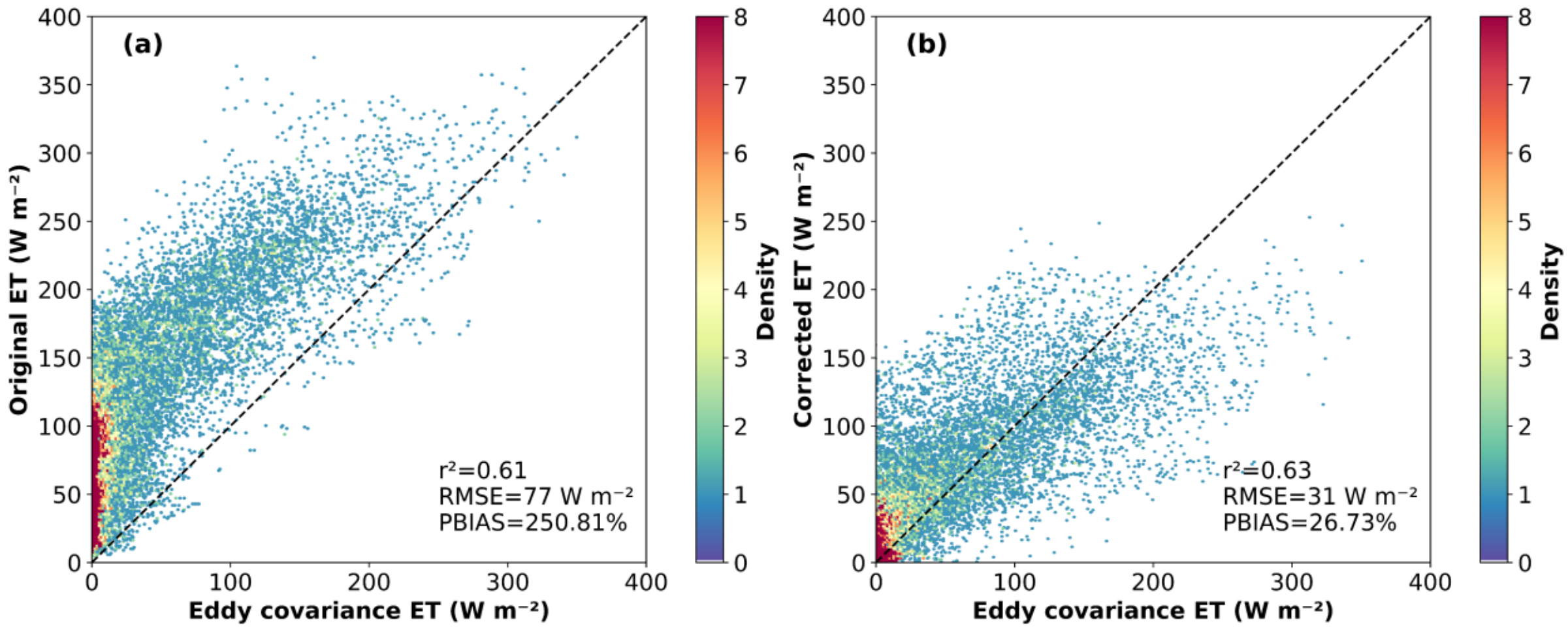

Fig. S26 The scatter plots of simulated ET against eddy covariance ET in high-latitude regions (north of 52°N) where ECOSTRESS coverage is absent: (a) before correction ($p < 1 \times 10^{-300}$, sample size = 24,864) and (b) after correction ($p < 1 \times 10^{-300}$, sample size = 24,864).

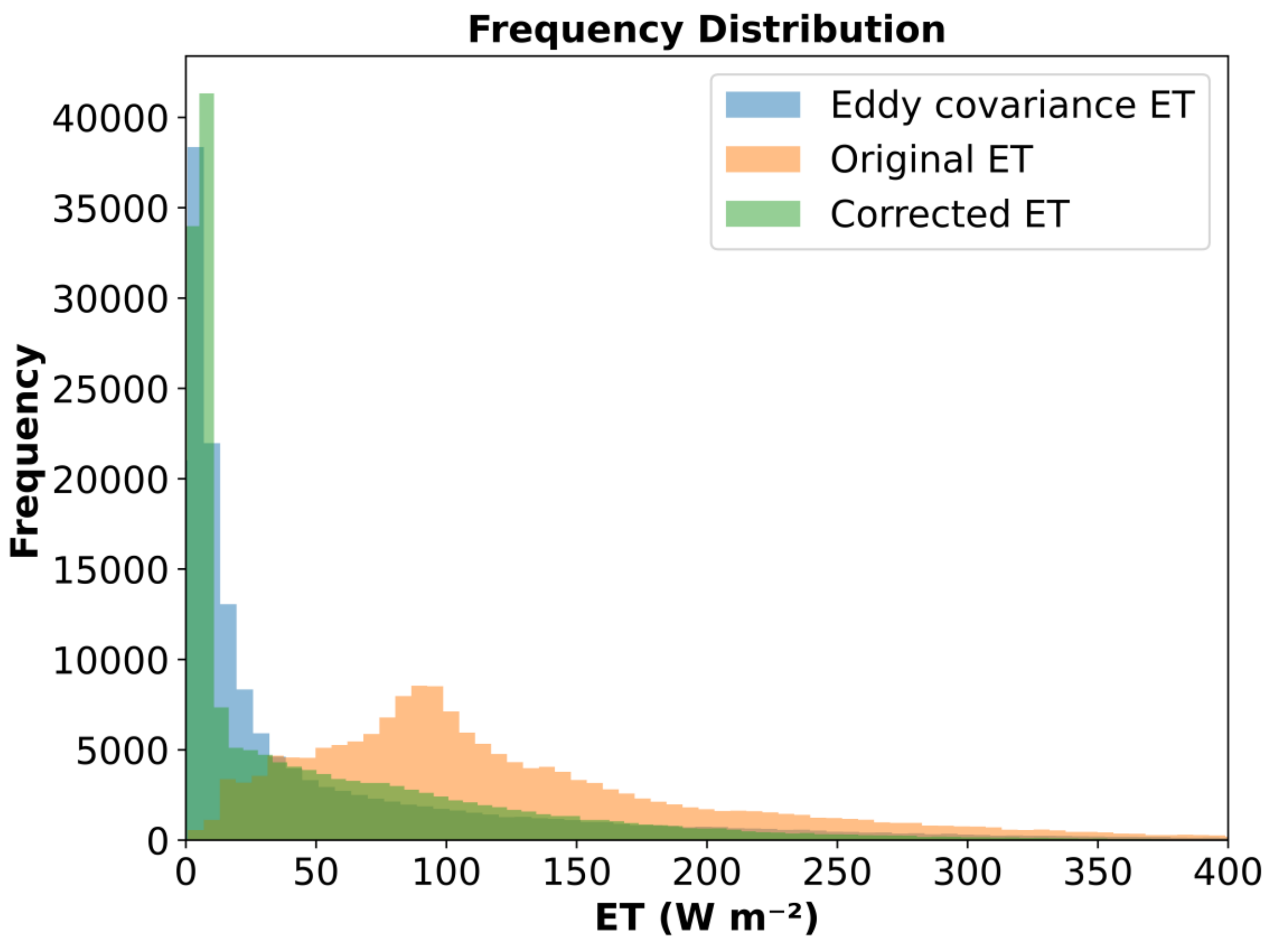

Fig. S27 Frequency distribution chart of eddy covariance ET, ECHO-ET estimates before correction, and ECHO-ET estimates with correction.

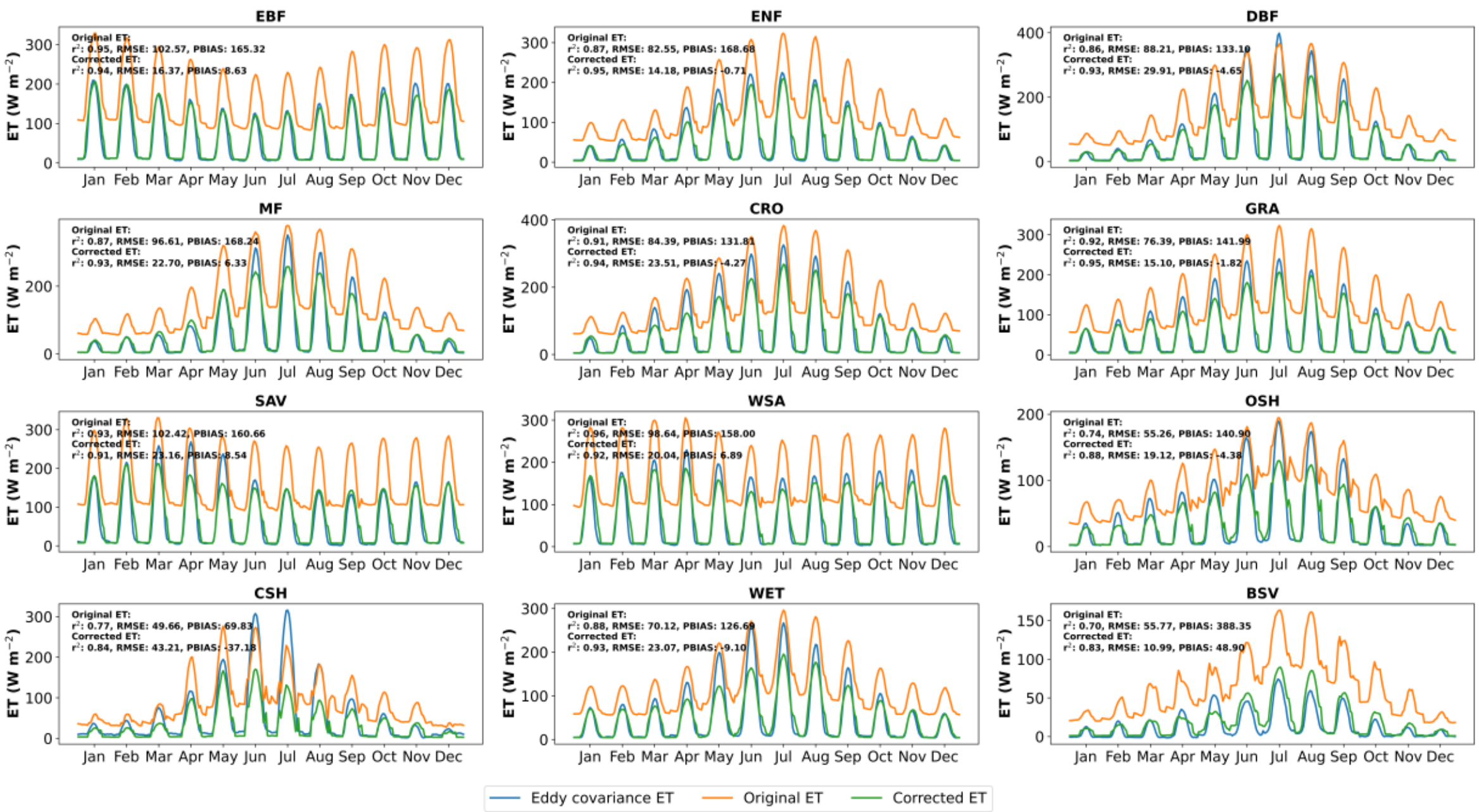

Fig. S28 Time series of eddy covariance ET, ECHO-ET estimates before correction, and ECHO-ET estimates after correction in each biome.

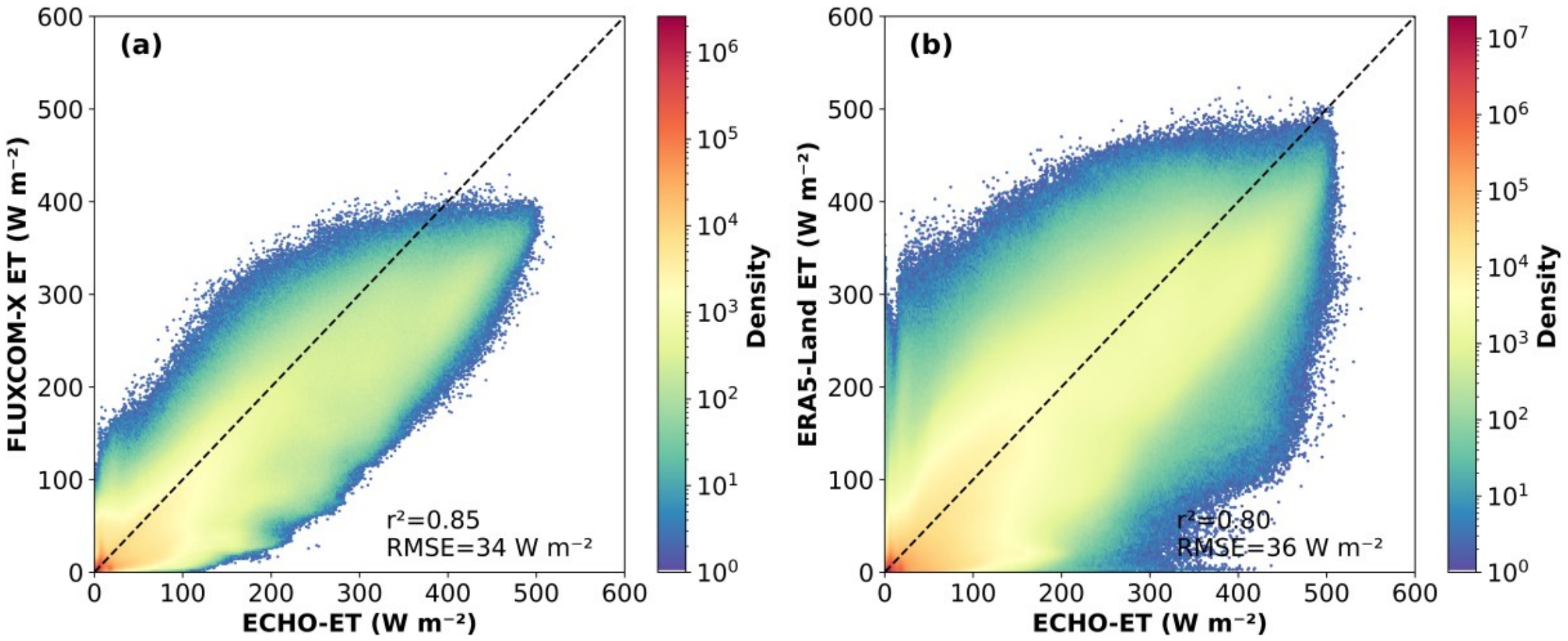

Fig. S29 The scatter plots of (a) FLUXCOM-X and (b) ERA5-Land ET against ECHO-ET at monthly-average diurnal scale (FLUXCOM-X: $p < 1 \times 10^{-300}$, sample size = 65,572,106; ERA5-Land hourly: $p < 1 \times 10^{-300}$, sample size = 412,319,539).

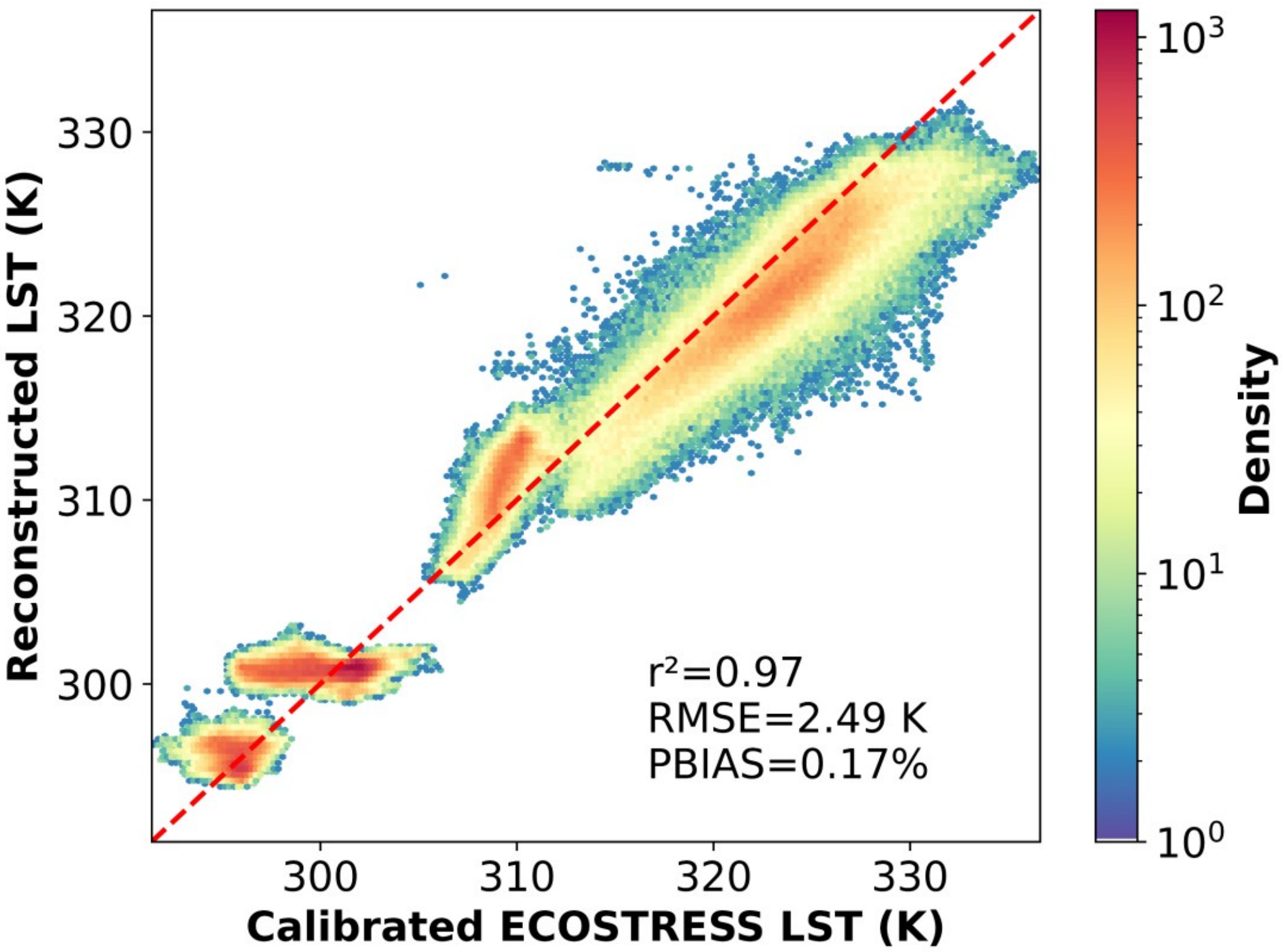

Fig. S30 The scatter plots of reconstructed LST against calibrated ECOSTRESS LST in the leave-one-out validation results ($p < 1 \times 10^{-300}$, sample size = 500,756).

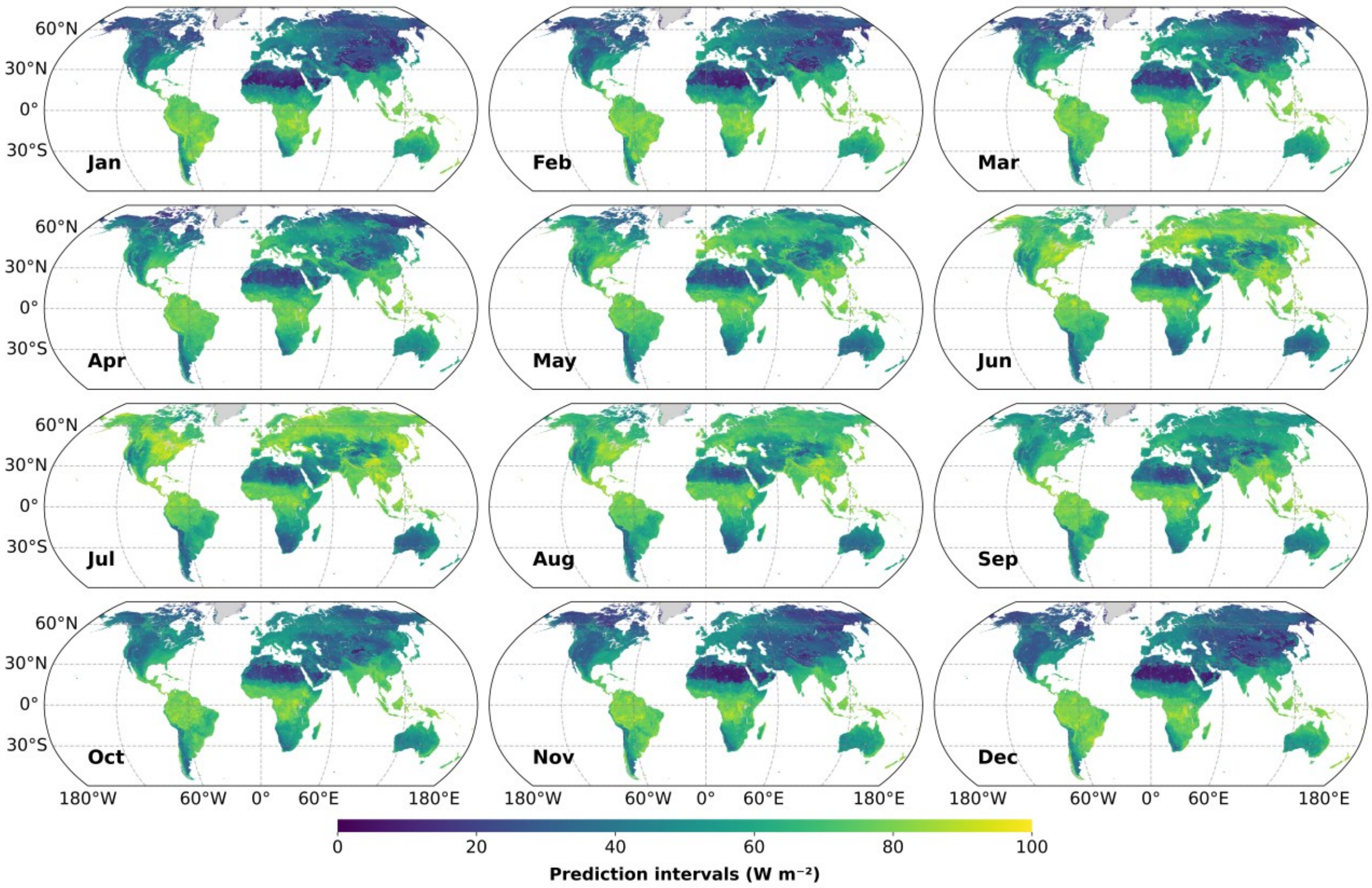

Fig. S31 Global patterns of daily-average hourly prediction intervals (PIs) for the Random Forest-based ET estimates.

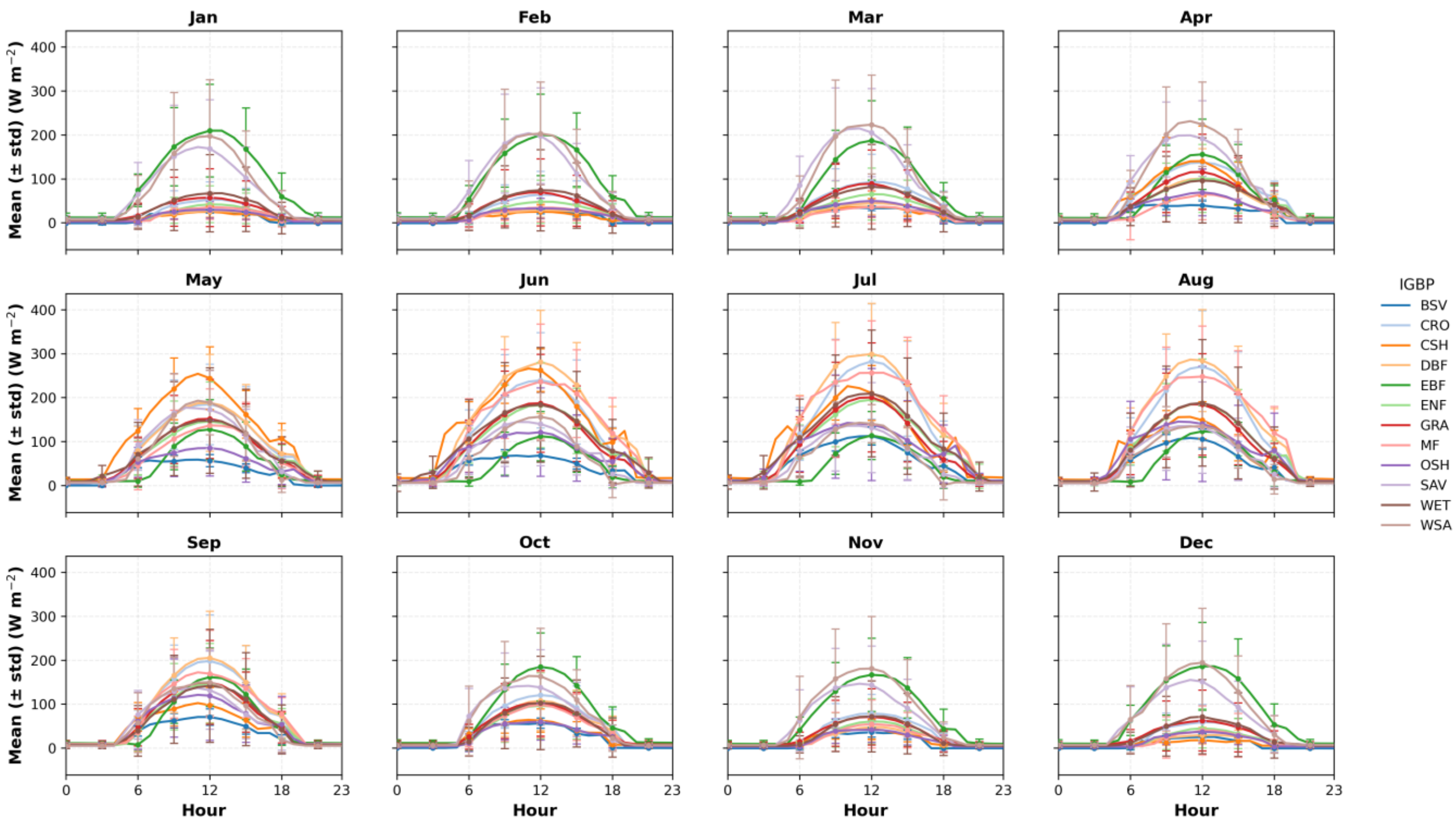

Fig. S32 Monthly diurnal cycles of hourly mean and standard deviation of corrected ECHO-ET across IGBP vegetation types.

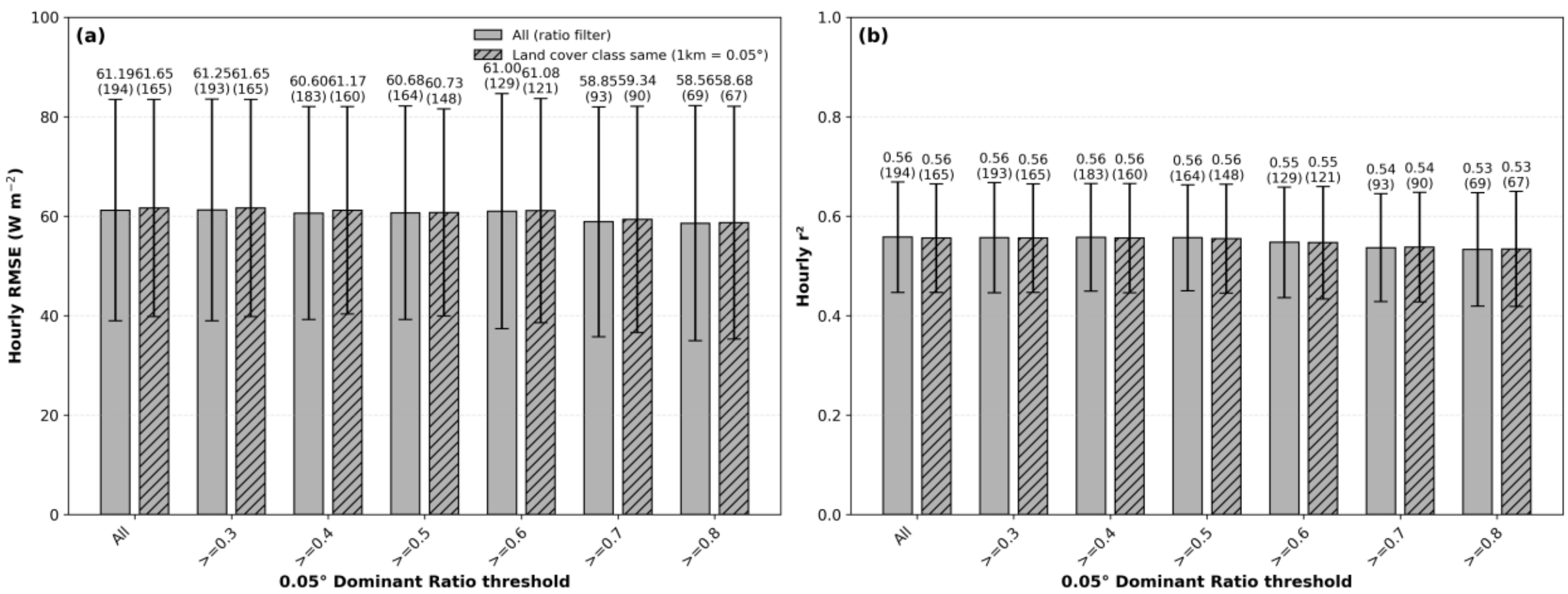

Fig. S33 Quantifying the impact of EC footprint-grid scale mismatch via land cover homogeneity thresholding and its effect on ECHO-ET validation ((a) RMSE and (b) r^2).

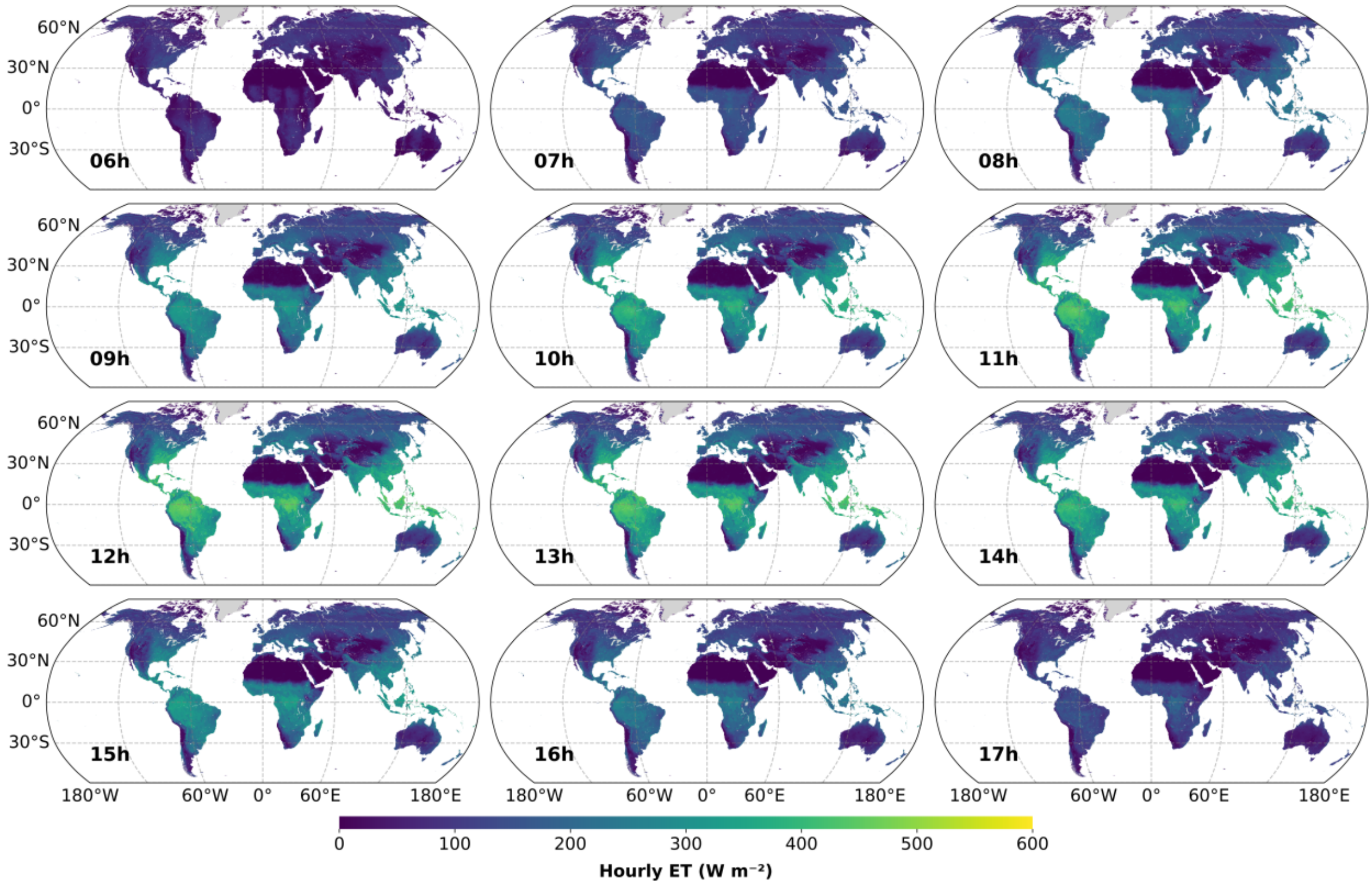

Fig. S34 Global diurnal patterns of ET during the peak-growing season.

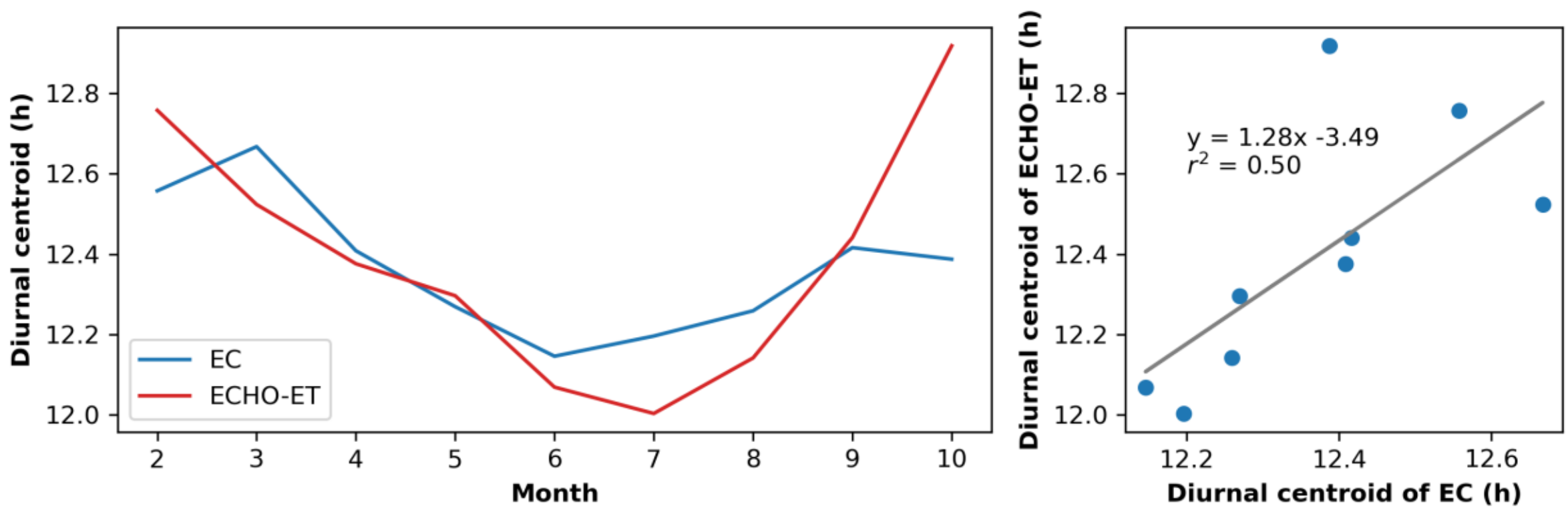

Fig. S35 Seasonal and correlation analysis of the diurnal centroid for ET (C_{ET}) above 60°N, derived from 23 eddy-covariance (EC) sites and ECHO-ET respectively.

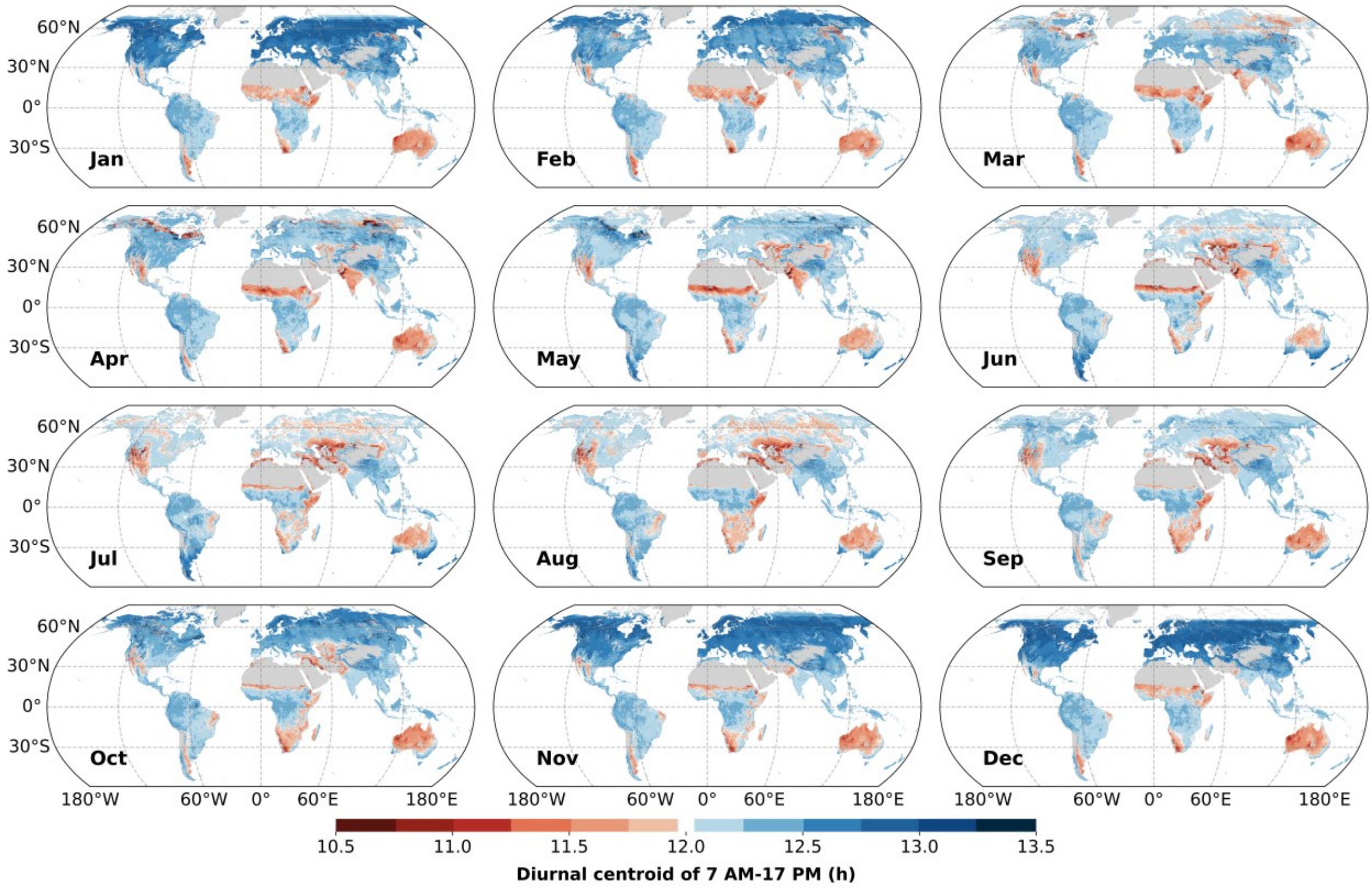

Fig. S36 Global patterns of the diurnal centroid for ET (C_{ET}) (7-17 h local solar time), derived from ECHO-ET.

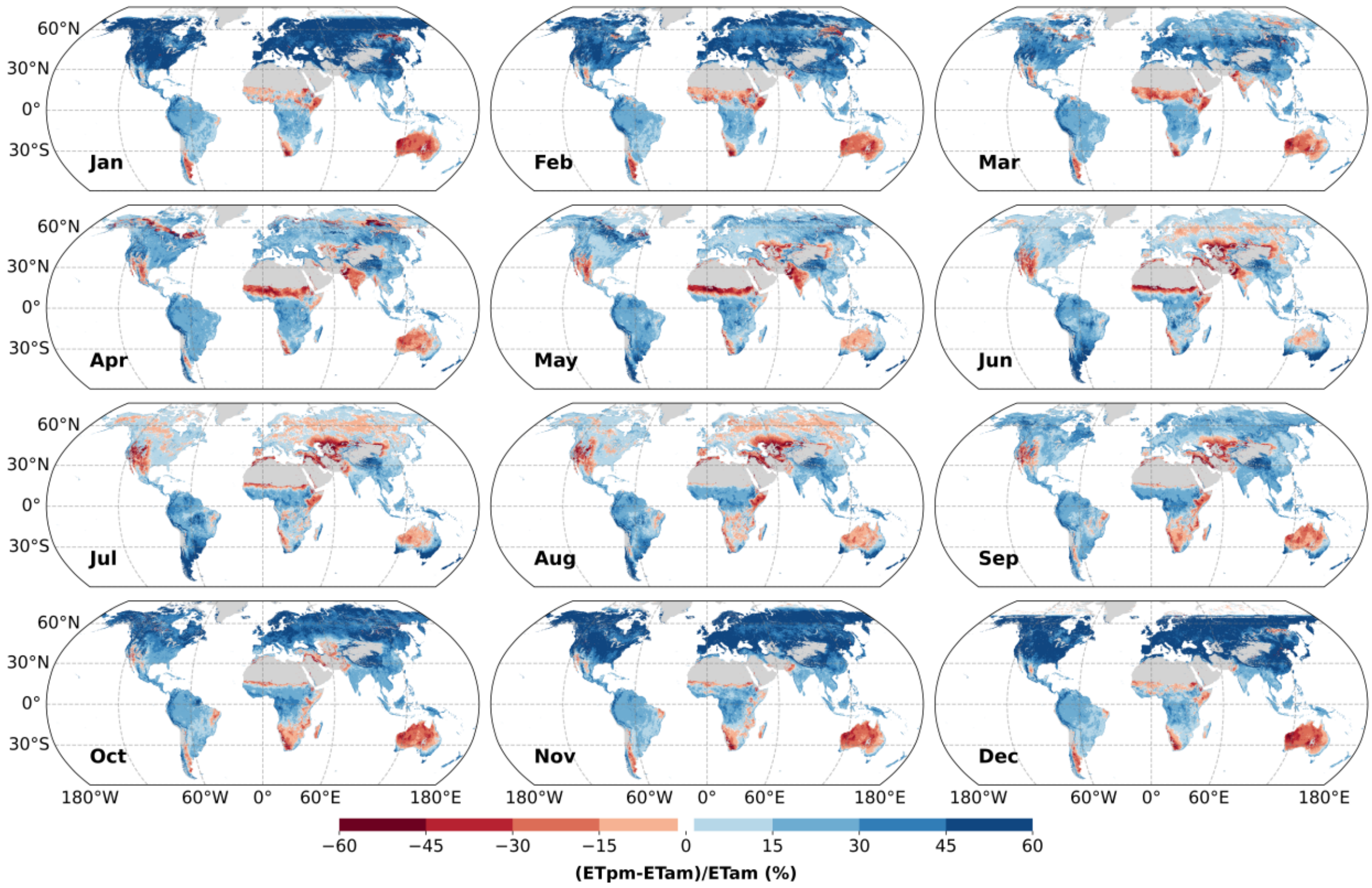

Fig. S37 Global patterns of the diurnal variation ((ET_{PM}-ET_{AM})/ET_{AM}) for gridded ET, derived from ECHO-ET.

Table S1 Summary of 0.05° ECOSTRESS ET samples.

**Land cover types**	**Count**
**Barren lands**	113426
**Closed shrublands**	9094
**Croplands**	243263
**Deciduous broadleaf forests**	28814
**Evergreen broadleaf forests**	48487
**Grasslands**	480658
**Mixed forests**	11911
**Needleleaf forests**	20952
**Open shrublands**	271539
**Savanna**	88978
**Urban**	3457
**Wetlands**	753
**Woody savanna**	64556
**Total**	1385888

Table S2 Metrics of Random Forest model validation.

Land cover types	r^2	RMSE ($W\ m^{-2}$)	PBIAS (%)
**Barren lands**	0.91	19	0.95
**Closed shrublands**	0.94	30	1.60
**Croplands**	0.91	27	-3.85
**Deciduous broadleaf forests**	0.95	32	-0.81
**Evergreen broadleaf forests**	0.92	44	-0.55
**Grasslands**	0.94	30	-0.43
**Mixed forests**	0.89	37	3.53
**Needleleaf forests**	0.84	46	4.25
**Open shrublands**	0.92	20	-0.07
**Savanna**	0.94	38	-1.50
**Urban**	0.83	49	4.65
**Wetlands**	0.88	48	2.43
**Woody savanna**	0.94	37	0.16

Table S3 Dataset names, links to access, and period covered.

Dataset names	Links to access	Period
**ECOSTRESS ET**	https://appeears.earthdatacloud.nasa.gov/	2018-2022
**ECOSTRESS LST**	https://appeears.earthdatacloud.nasa.gov/	August 2018
**ECOSTRESS cloud mask**	https://appeears.earthdatacloud.nasa.gov/	August 2018
**ERA5-Land reanalysis**	https://cds.climate.copernicus.eu/cdsapp#!/home	2018-2022
**AmeriFlux**	https://ameriflux.lbl.gov/	Supplementary Data 1
**ICOS**	https://www.icos-cp.eu/	
**OzFlux**	https://www.ozflux.org.au/	
**NEON**	https://ameriflux.lbl.gov/	
**HiWATER**	https://data.tpdc.ac.cn/	
**hPET**	https://doi.org/10.5523/bris.qb8ujazzda0s2aykkv0oq0ctp	2020
**MODIS MCD43C4**	https://search.earthdata.nasa.gov/	2018-2022
**MODIS MCD12C1**	https://search.earthdata.nasa.gov/	2020
**Landsat Collection 2 surface reflectance**	https://developers.google.com/earth-engine/datasets/catalog/LANDSAT_LC08_C02_T1_L2	August 2, 17, and September 2, 2018
**Köppen-Geiger climate classification**	https://hess.copernicus.org/articles/11/1633/2007/	-
**Palmer Drought Severity Index (PDSI)**	https://climate.northwestknowledge.net/TERRACLIMATE/index_directDownloads.php	2018-2022
**Standardized Precipitation Evapotranspiration Index (SPEI)**	https://zenodo.org/records/8060268	2019-2020
**FLUXCOM-X**	https://www.icos-cp.eu/data-products/fluxcom-x-global-fluxes-collection	2020
**ESA WorldCover 10m v200**	https://developers.google.com/earth-engine/datasets/catalog/ESA_WorldCover_v200	2021
