# Supplementary material for: NASA’s ECOSTRESS satellite reveals widespread midday depression in ecosystem evapotranspiration
Source: Nat Commun. 2026 Aug 25;17:8976. doi: 10.1038/s41467-026-76873-x (PMC13507112; doi:10.1038/s41467-026-76873-x)
Supplement: Supplementary file 2 [file 41467_2026_76873_MOESM2_ESM.pdf]

nature portfolio

Peer Review File

NASA’s ECOSTRESS satellite reveals widespread midday depression in ecosystem evapotranspiration

Corresponding Author: Professor Jingfeng Xiao

This file contains all reviewer reports in order by version, followed by all author rebuttals in order by version.

Version 0:

Reviewer comments:

Reviewer #1

(Remarks to the Author)
This manuscript investigates midday evapotranspiration (ET) depression using ECOSTRESS satellite observations combined with machine learning techniques. Midday ET depression has increasingly been identified from site to regional scales, and this study has the potential to contribute to our understanding of short-term water cycle dynamics at broader scales. However, in its current form, the manuscript faces several conceptual and methodological issues that may limit its suitability for Nature Communications. I encourage the authors to carefully address the concerns outlined below and to consider submit this work to a remote-sensing-focused journal (e.g., Remote Sensing of Environment).
General Comments
1. Uncertainty in ET estimation
ET quantification from satellite observations is inherently challenging due to complex interactions between water and energy fluxes across heterogeneous environments. While the authors integrate satellite data with machine learning to estimate daily ET, the associated uncertainties appear to be underestimated. 1) Data integration: The study combines datasets of vastly different spatial and temporal resolutions (e.g., Landsat: 30 m/16-day; MODIS: ~5.5 km/daily; ECOSTRESS: 70 m/hourly; ERA5-L: ~10 km/hourly; EC flux sites: varying footprints). Such heterogeneity introduces substantial uncertainty during interpolation, resampling, and model training, which requires a more explicit evaluation. 2) Model drivers: The ET model relies on climatic variables and NIRv, but excludes key predictors such as precipitation and LAI, which strongly influence ET dynamics, especially on interannual scales. The rationale for their omission needs clarification. 3) Ground-truth reference: EC-derived ET is used as the benchmark, yet its accuracy depends on the reliability of latent heat flux measurements. Potential biases—particularly during the first or last years of site operation due to instrument adjustments or malfunctions—should be discussed. Furthermore, the scale mismatch between flux footprints and satellite pixels warrants deeper consideration. 4) Compounding uncertainties: Although corrections have been applied, the complexity of the processes considered may amplify uncertainty. A more rigorous uncertainty quantification is needed.
2. Analysis of drought and heatwave impacts
The manuscript analyzes several case studies to examine the influence of drought and heatwaves on diurnal ET cycling. While illustrative, this approach weakens the generality and robustness of the conclusions. What is the global pattern linking droughts/heatwaves to ET cycling? Without broader analyses, the findings remain anecdotal. Temperature is the primary forcing of both droughts and heatwaves. Why not directly assess temperature anomalies instead of focusing only on climatic indices? The analysis currently lacks causal inference. Correlation analyses or causal frameworks are needed to substantiate the claim that droughts and heatwaves alter diurnal ET dynamics.

Specific comments:
Line 3. The title equates ET with plant water use, which is inaccurate. ET encompasses both transpiration and evaporation and is more closely related to water-use efficiency. The title should be revised for precision.
Line 28-29. It is expected that ERA5-L hourly reanalysis may not fully capture midday ET depression. The key question is why: what processes are missing, and what are the implications for improving reanalysis products?
Line 43-83. The extended introduction on flux tower limitations, satellite advantages, and ECOSTRESS features could be shortened. This style is more typical of remote sensing journals and may be excessive for Nature Communications.
Line 309. Direct comparisons without process-level evaluation are of limited value. While a full mechanistic analysis may be beyond scope, readers would benefit from discussion of why ERA5-L underperforms in this context and how such datasets might be improved.

Reviewer #2

(Remarks to the Author)
General comments to Author:

Review by William M. Hammond, Assistant Professor of Plant Ecophysiology, University of Florida:

This manuscript leverages high-resolution data from ECOSTRESS and ML models to develop high-resolution (in time and space) predictions of ET. This highly original work is significant in its global scale, groundbreaking in its revealing sub-daily patterns of ET (and especially, the timing of ET maxima and depression), and is likely to be of considerable interest across many fields working on land plants, terrestrial ecosystems defined by land plants, and communities working on earth system modeling. Overall I found the manuscript to be well-written, the figures and analyses are compelling, and the conclusions drawn are well supported by the evidence presented. I do have on general comment regarding how plant stomatal responses are framed in the paper, and I think considering this carefully and including the biophysical component (not just active plant responses) would strengthen the manuscript and perhaps also provide a more likely mechanism to point at to support to these primarily globally-synchronous findings regarding midday ET depression. The introduction frames the importance of stomatal closure in "conserving" water (e.g., some stimulus-response is at work; cited literature points to ABA etc.). However, another reason for the midday depression could be more passive—the declining water status (and loss of turgor pressure) in leaves. I think a more rigorous treatment of how and why stomata close in the introduction would strengthen the paper as it can point to the broadly observed biophysical basis for the phenomenon observed. Adding this would also strengthen conclusions drawn regarding the earlier onset of CET during droughts and heatwaves, as both are associated with lower water content (initially, or through time) in plants, leading to reduced turgor pressure and/or a more rapid acceleration toward closure. References regarding this https://doi.org/10.1111/pce.12774, have found ~80% of stomatal closure can be attributed to the biophysical consequence of reduced turgor, rather than to changes in light, intercellular CO_2 concentration, or other factors. As all land plants share stomata that are operated on the basis of turgor pressure, including this framing helps to support the globally cohesive observations, and the observed CET shifts to changing environmental conditions. Overall, I found this paper very exciting to read. The dataset generated will likely fuel many interesting studies on plant-environment interactions, and has profound implications for ongoing and further changes in the Earth system. Below, I include my line-specific comments for the authors to consider.

Line-specific comments:
L19-20: From the opening sentence, the depressing in water use is framed as a plant strategy to limit the loss of water, rather than a constraint on the ability of the plant to sustain maximum water transport. The authors should carefully consider if the evidence supports the current framing.
L35: Stomata close for more reasons than water conservation, this could be changed "and conserve water upon closure" for example, to highlight what you're emphasizing without making stomatal closure sounding single-purpose.
L37-39: By mid-day, it is also more likely that other mechanisms of stomatal closure (e.g., increasing CO_2 concentration in the leaf) may take place
L189: 'vegetative activity' is vague, plants have many active processes that proceed with or without active or maximum ET.
L190: change 'their' to 'its'
L200: consider changing 'extended to the left' to 'extended to earlier hours' or something more unit-specific.
L204-209: This is interesting. If you consider the biophysical constraints on stomatal closure, having a more consistent environment with a more consistent canopy (the EBF tropical forests have on average less variation in canopy area, canopy age, throughout the year than other types investigated), this could be another reason that small differences are observed in EBF. I am not sure there is sufficient evidence to support the current interpretation, "indicating the resilience of rainforests". Resilience would indicate an ability to recover from a non-optimal state. I guess 'tolerance' could fit here, but that doesn't seem quite right either. I interpreted your results to mean that EBF have a more consistent climate (water availability, temperature), thus the supply (water in ground, water in plant tissues), and demand (Atmospheric demand, but also the surface area cross which it is applied!) are more constant. Please consider being more nuanced here than pointing to resilience of rainforests. There is continental-scale evidence that some of Earth's wettest forests may have the narrowest of margins for tolerance and resilience to non-optimal conditions (e.g., as in https://doi.org/10.1111/gcb.15641). Pointing to stability in the CET as an indicator of resilience might build false confidence in these systems, when the CET stability may instead be emerging from not just environmental, but also structural stability in the plants/canopies themselves, which is more typical in EBF than other plant functional types.
L226: It would be interesting to include the dominant PFT(s) within each of these regions, noting it even just textually, to link to the prior section.
L250: Love this figure. Would be clearer if you replaced the _d and _n with clearly labeled "normal years" and "hotter-drought years" or something. This could be done in the legend for lines beneath the last panels, as it's got plenty of room.
L373: Consider expanding this sentence a bit. For example, stating a more complete picture like …. "Stress results initially in reduced turgor pressure, stomatal closure, and eventually wilting. Under prolonged, severe stress, embolism may form—and eventually lead to death of cells, organs, or whole organisms." Would leave the reader more informed than "this stress results in xylem embolism or leaf wilting". References supporting this sequence would/could include: https://doi.org/10.1038/s41586-018-0240-x; https://doi.org/10.1038/s43017-022-00272-1; and https://doi.org/10.1111/nph.12798.

L373-377: Here, the signal-response framing again is dominant, and no mention of the passive biophysical result of lost water at the cell-to-leaf scale, that is a plausible, evidence-supported mechanism for the observed mid-day depression in ET

as stomatal turgor cannot be sustained for maximum transpiration; all land plants are subject to these same constraints, where there exist a much greater variety in the biochemical responses/mechanisms that would make it a bit harder to support/explain such a globally cohesive patterning in midday ET dynamics!

L381: Peaks earlier in the day do avoid additional water loss during increased temperature, but to avoid heat stress, plants must transpire MORE, and not less (to lower leaf surface temperatures, or return the living cells to thermal optima for biochemical processes). Consider re-phrasing this to add in some of this important nuance.
L397: This is great. Consider adding the information that when comparing within the same location, across time, that midday depression is a stress indicator; comparing between sites this may not always strictly speaking be true (I don't think the paper currently asserts this, obviously for a variety of reasons the CET may be different due to plant structural, community compositional, dominant photosynthetic pathway, or other factors).

L405-407: Agreed! The utility of midday depression as a remotely-sensed signal from the plant (not the environment) is the reason I was most excited to read and review this paper.

L419: It remains unknown if under extreme heat waves, if plants will keep their stomata closed, or not. Whether this is driven biochemically or biophysically (as in the 'wrong-way response'), is uncertain. The methods developed her may help to investigate these emerging phenomenon, as heat extremes that are novel in the current era are emerging more frequently, for example the 2021 PNW heat dome, where heat caused significant damages. It is unclear if running out of water (as proposed in: https://doi.org/10.1093/treephys/tpac007) or getting too hot (as we proposed: https://doi.org/10.1093/treephys/tpac143) were dominant mechanisms of ecosystem-scale canopy die-back. Having a means to monitor sub-daily ET will be a fantastic tool to study these phenomena, and even to understand the mechanisms that predominate during such extreme events.

Reviewer #3

(Remarks to the Author)
The manuscript presents an important study on quantifying the global midday depression in plant evapotranspiration (ET) using ECOSTRESS observations and machine learning. The topic is timely and relevant, especially in the context of plant water stress under drought and heatwave conditions. However, there are several critical issues that need to be addressed before the manuscript can be considered for publication:
1. The manuscript repeatedly refers to "machine learning" in general terms. For reproducibility and clarity, the authors should explicitly state the specific algorithm(s) employed and provide details on model training, feature selection, and validation.
2. While the study highlights the advantages of ECOSTRESS, a comparative evaluation using other global ET products (e.g., MOD16, FLUXCOM, GLEAM, ERA5-Land) is necessary. Such comparisons would help demonstrate the added value of ECOSTRESS and contextualize the findings against existing datasets.
3. ECOSTRESS data is limited to latitudes between ~52°N and 52°S. The manuscript should discuss the implications of this coverage limitation, including potential uncertainties or biases in regions outside the covered area. The authors should also clarify whether the machine learning approach extrapolates to higher latitudes, and if so, how reliable such extrapolations are.
4. Include uncertainty estimates for the machine learning ET predictions.
5. Clarify the relationship between observed midday depression and shifts in peak ET (CET) across different climatic zones.
6. Consider discussing the temporal resolution of ECOSTRESS and its implications for capturing diurnal ET dynamics.

Version 1:

Reviewer comments:

Reviewer #1

(Remarks to the Author)
Thanks for your great work in the revision. My comments have been well addressed. I have no further questions.

Reviewer #2

(Remarks to the Author)
PEER REVIEW for "NASA's ECOSTRESS satellite reveals widespread midday
depression in ecosystem evapotranspiration" by Bu et al., submitted to Nature Commuunications
Peer review by William M. Hammond

General comments:
The new analysis considering the impacts of drought and heatwaves on ET is novel and provides another clear example of the utility of the global ECHO-ET dataset. By adding the analysis for heatwave stress, the authors have revealed that compound "hotter droughts" are most responsible for midday ET depression; this builds globally on a site- and plot-based series of observations that hotter-drought is driving plant stress and mortality globally in forests, for example. The authors could connect this new robust analysis to recent literature on these topics, pointing out that evidence from multiple approaches and scales are pointing towards the same conclusion: warming, especially atop drought stress, is increasingly

challenging the vital role of plants in ecosystems.

The authors have carefully re-frame their interpretation of plant water use to include a wider array of evidence (e.g., biophysical mechanisms, intracellular CO2 concentrations, and active hormonal controls. In this revision, the authors have sufficiently addressed my concerns with the prior version of the manuscript. In particular, the addition of an impacts of drought and heatwaves on diurnal ET cycles adds important context, and builds confidence in the ECHO-ET product. I expect that its contribution of the new ECHO-ET dataset, and the knowledge revealed here in its first application, will both be of high impact and interest to the readers of Nature Communications. A few line-specific comments follow, aimed at improving clarity of the manuscript and its figures.

Line-specific comments:
L33: change 'uses' to 'use'
L71: add a space between 'the' and '1'
L77-78: This could be clearer, something like "While covering a broad portion of Earth's land surface, earlier …(the existing sentence, without 'also')."
L274-276: This is an excellent contribution to our knowledge of drought vs. heatwave impacts, at such a broad scale. Well done!

L304-324: Fig. 6: Clearer legend labels at bottom of this figure. Are you sure this is 'drought years' and not 'hotter drought' years, based on the new analyses and importance of warming atop drought in driving observations? Even if the analyses were filtered for drought, did you check that these years were "average" in temperature" if they are warmer than long-term, please clearly label as hotter-drought or similar to provide a fuller picture of the difference in drivers between normal and anomalous years.

L383: "13PM" is odd; consider 13:00 or 01:00PM; I think 13:00 to be consistent, check all reported timings and report in the same standard/format, a 24h clock.

L443-445: Great job synthesizing these important mechanisms. In the final sentence of the paragraph, I think reminding the reader that this conclusion is supported by ECHO-ET at its scale of observation. Perhaps a sentence following this one, could indicate that ECHO-ET is sensing the canopy-scale over relatively broad areas, compared to cells, organs, and organisms which all likely vary in their dynamics at those finer spatial scales; nevertheless, the emergence of a globally coherent midday depression in ET really highlights the importance of this passive biophysical consequence of dehydration over signal-response. Interesting!

Reviewer #3

(Remarks to the Author)
No more comments.

Open Access This Peer Review File is licensed under a Creative Commons Attribution 4.0 International License, which

permits use, sharing, adaptation, distribution and reproduction in any medium or format, as long as you give appropriate credit to the original author(s) and the source, provide a link to the Creative Commons license, and indicate if changes were made.
In cases where reviewers are anonymous, credit should be given to 'Anonymous Referee' and the source.
The images or other third party material in this Peer Review File are included in the article's Creative Commons license, unless indicated otherwise in a credit line to the material. If material is not included in the article's Creative Commons license and your intended use is not permitted by statutory regulation or exceeds the permitted use, you will need to obtain permission directly from the copyright holder.
To view a copy of this license, visit https://creativecommons.org/licenses/by/4.0/

Responses to Reviewers' Comments

Review round: 1

Reviewer #1 (Remarks to the Author):

This manuscript investigates midday evapotranspiration (ET) depression using ECOSTRESS satellite observations combined with machine learning techniques. Midday ET depression has increasingly been identified from site to regional scales, and this study has the potential to contribute to our understanding of short-term water cycle dynamics at broader scales. However, in its current form, the manuscript faces several conceptual and methodological issues that may limit its suitability for Nature Communications. I encourage the authors to carefully address the concerns outlined below and to consider submit this work to a remote-sensing-focused journal (e.g., Remote Sensing of Environment).

Response: Thank you for this thoughtful assessment. We sincerely appreciate your constructive and insightful comments and the opportunity to clarify the conceptual contribution and broader relevance of our study.

While our framework leverages ECOSTRESS observations and machine learning, the central scientific advance lies in quantifying and interpreting **global diurnal water stress dynamics**, rather than in algorithm development itself. Specifically, our study is the first to quantify global midday depression in plant water use using a gapless, gridded hourly evapotranspiration (ET) product and to reveal its spatial and temporal structure across climate regimes and vegetation types. We believe that our manuscript fits into the scope of *Nature Communications* well.

Previous research has documented midday depression of ET or photosynthesis, primarily through eddy covariance (EC) measurements at individual sites. These EC towers typically cover footprints ranging from a few hundred meters to several kilometers, capturing highly localized ecosystem behavior. While such studies provide valuable mechanistic insights into stomatal regulation and energy–water coupling, their findings cannot be directly generalized to continental or global scales due to the sparseness of the EC sites and the significant spatial heterogeneity in climate, vegetation, and hydrologic regimes. Additionally, while recent studies utilizing data from geostationary satellites have begun investigating diurnal cycles of photosynthesis, no research has systematically examined global midday depression in plant water use. Geostationary satellites are constrained by limited spatial coverage, requiring complex mosaicking for global applications, and their kilometer-scale resolution restricts detection of finer-scale heterogeneity, such as field-level crop responses or patch-scale forest dynamics.

In contrast, our work leverages the novel ECHO-ET product, which integrates ECOSTRESS observations with machine learning into a globally consistent, hourly ET dataset. This allows for the first systematic quantification of midday ET depression on a global scale. Importantly, our analysis reveals that existing global hourly ET products have not captured this phenomenon,

highlighting the unique contribution of our approach to understanding global plant water use dynamics.

Importantly, our findings extend beyond detection. By quantifying the severity of midday depression globally, we reveal widespread diurnal water stress and demonstrate that midday depression emerges from the divergence between water limitation and energy limitation regimes. This provides new insight into short-term ecohydrological regulation and the coupling between plant hydraulics and atmospheric demand at broad scales. The implications are directly relevant to understanding climate–vegetation feedbacks, drought intensification, and sub-daily land–atmosphere interactions—topics that align closely with the interdisciplinary scope of *Nature Communications*.

In response to your suggestion, we have revised the manuscript to more clearly position our contributions, explicitly highlighting how our work builds upon—but fundamentally extends—prior studies from local and regional observations to a global, process-oriented perspective.

We believe that the novelty and broad audience of this effort and implications for understanding global ecohydrological dynamics make this work suitable for *Nature Communications*, a broad interdisciplinary journal.

General Comments

1. Uncertainty in ET estimation

ET quantification from satellite observations is inherently challenging due to complex interactions between water and energy fluxes across heterogeneous environments. While the authors integrate satellite data with machine learning to estimate daily ET, the associated uncertainties appear to be underestimated. 1) Data integration: The study combines datasets of vastly different spatial and temporal resolutions (e.g., Landsat: 30 m/16-day; MODIS: ~5.5 km/daily; ECOSTRESS: 70 m/hourly; ERA5-L: ~10 km/hourly; EC flux sites: varying footprints). Such heterogeneity introduces substantial uncertainty during interpolation, resampling, and model training, which requires a more explicit evaluation.

Response: Thank you for this important comment. We agree that integrating multi-source datasets with very different spatial and temporal resolutions can introduce uncertainty through resampling, interpolation, and scale mismatch. In the revised manuscript, we have strengthened our uncertainty assessment by adding new analyses to explicitly address these issues at multiple levels.

First, we quantified predictive uncertainty in the RF-based ECHO-ET estimates using a split conformal prediction framework. This approach provides distribution-free, finite-sample calibrated prediction intervals with guaranteed empirical coverage, independent of model complexity. The dataset was divided into model-fitting and independent calibration subsets, and uncertainty was derived from the variability across individual trees within the random forest. The resulting prediction intervals were calibrated to achieve nominal 90% coverage and were evaluated on an independent test set, where empirical coverage was consistent with the

nominal level. Importantly, because conformal prediction guarantees empirical coverage regardless of model complexity or input heterogeneity, uncertainties introduced during spatial harmonization and resampling are reflected in the calibrated prediction intervals. In the revision, we have added this analysis in the MATERIALS AND METHODS (Lines 792-822) and included the figure in the supplement (Fig. S31, as shown below).

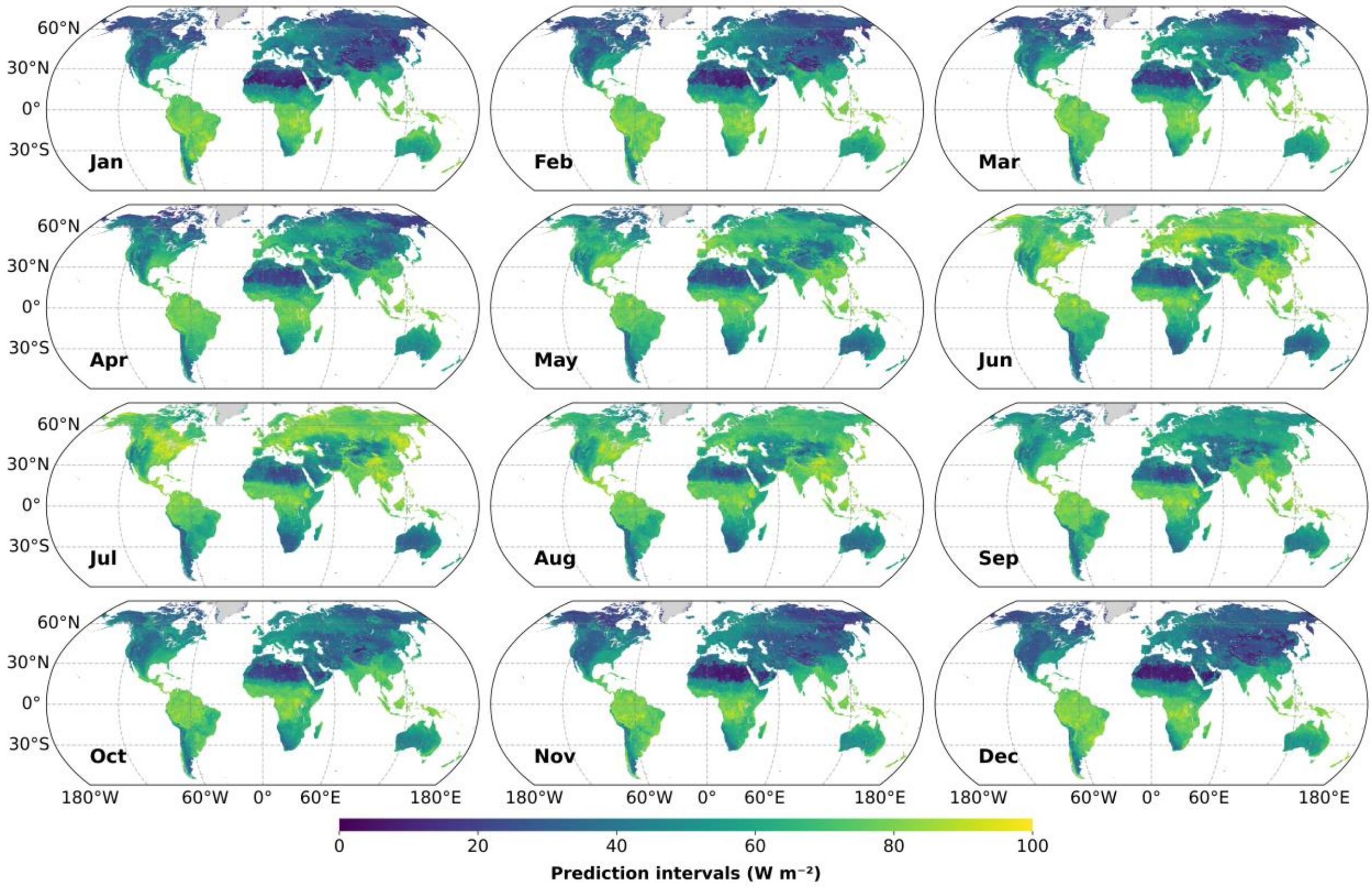

Fig. S31 Global patterns of daily-average hourly prediction intervals (PIs) for the Random Forest-based ET estimates.

Second, we followed the reviewer's useful suggestion and directly evaluated the influence of spatial scale mismatch between EC tower footprints (hundreds of meters) and the 0.05° (~5 km) ECHO-ET grid. Sites were filtered based on within-pixel land-cover heterogeneity using the 10 m WorldCover dataset. Dominant land-cover fractions were computed both at the grid scale and within a 1 km tower-centered buffer. Sensitivity analyses were conducted across multiple homogeneity thresholds and with an additional constraint requiring agreement between grid-scale and footprint-scale dominant classes. Across all thresholds, hourly RMSE and R^2 values remained relatively stable, with only marginal improvements under stricter homogeneity filtering. These results indicate that although scale mismatch contributes to uncertainty, it is not the primary source of model error. In the revision, we have added this analysis in the MATERIALS AND METHODS (Lines 841-857) and included the figure in the supplement (Fig. S33, as shown below).

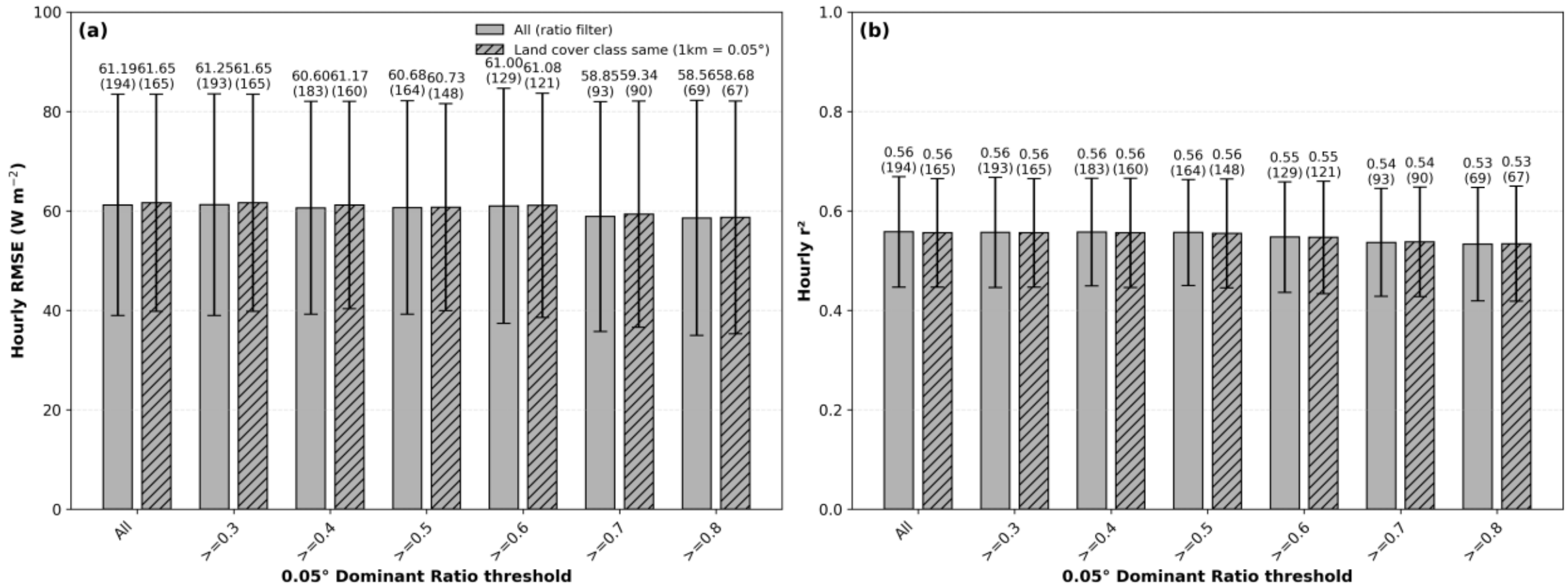

Fig. S33 Quantifying the impact of EC footprint-grid scale mismatch via land cover homogeneity thresholding and its effect on ECHO-ET validation ((a) RMSE and (b) r^2).

Overall, uncertainty in ECHO-ET is addressed through a combination of calibrated prediction intervals that capture model/input heterogeneity as well as sensitivity analyses of footprint–grid scale mismatch. Together, these complementary assessments demonstrate that uncertainties associated with multi-resolution data integration are explicitly evaluated and are not underestimated in our framework.

2) Model drivers: The ET model relies on climatic variables and NIRv, but excludes key predictors such as precipitation and LAI, which strongly influence ET dynamics, especially on interannual scales. The rationale for their omission needs clarification.

Response: We did not include precipitation and leaf area index (LAI) in the baseline model. Precipitation influences ET primarily through its effects on soil moisture storage and availability rather than through direct, instantaneous controls on surface fluxes. These effects are already explicitly represented by root-zone soil moisture, which integrates antecedent precipitation and hydrologic memory over relevant time scales. Including precipitation as an additional predictor would therefore provide limited independent information while increasing redundancy among hydrometeorological variables.

Similarly, LAI conveys information on canopy structure and greenness that largely overlaps with that captured by NIRv and related vegetation indices, which have been shown to be strongly correlated with photosynthetic activity and canopy density. Adding LAI would thus introduce substantial collinearity with existing vegetation proxies, potentially inflating model uncertainty without clear gains in predictive skill. Moreover, LAI products often differ in spatial resolution, retrieval assumptions, and temporal sampling relative to other inputs, which could exacerbate scale-mismatch and propagation of observational uncertainty. In addition, LAI products typically have substantial uncertainty, which could lead to significant uncertainty to our hourly ET estimates.

For these reasons, we excluded P and LAI from the baseline model to reduce multicollinearity and to ensure more robust and interpretable model behavior. We have added some sentences

(Lines 682-685) to clarify the rationale behind the omission of precipitation and LAI.

3) Ground-truth reference: EC-derived ET is used as the benchmark, yet its accuracy depends on the reliability of latent heat flux measurements. Potential biases—particularly during the first or last years of site operation due to instrument adjustments or malfunctions—should be discussed. Furthermore, the scale mismatch between flux footprints and satellite pixels warrants deeper consideration.

Response: We agree that the reliability of EC-derived ET as ground truth depends on measurement quality and that potential biases—particularly during the early or late years of site operation—should be carefully examined. We have addressed these concerns through additional sensitivity analyses and expanded discussion.

Regarding potential biases during the first or last years of site operation, we performed a sensitivity analysis excluding the initial and final years of each EC site record to assess potential start-of-operation calibration issues or end-of-operation sensor degradation and malfunctions. The model was re-evaluated after removing these boundary years. As shown in the figure below, the validation metrics after excluding the first and last years of each site record (RMSE = 45.004 W m^{-2} and r^2 = 0.718, as shown below) were virtually identical to those obtained from the full-record analysis (RMSE = 45.030 W m^{-2} and r^2 = 0.717), indicating negligible influence of potential start- or end-of-operation biases. This indicates that potential early- or late-stage instrumental adjustments do not materially affect the calibration or validation of ECHO-ET. In the revision, we have discussed this in the MATERIALS AND METHODS (Lines 718-726).

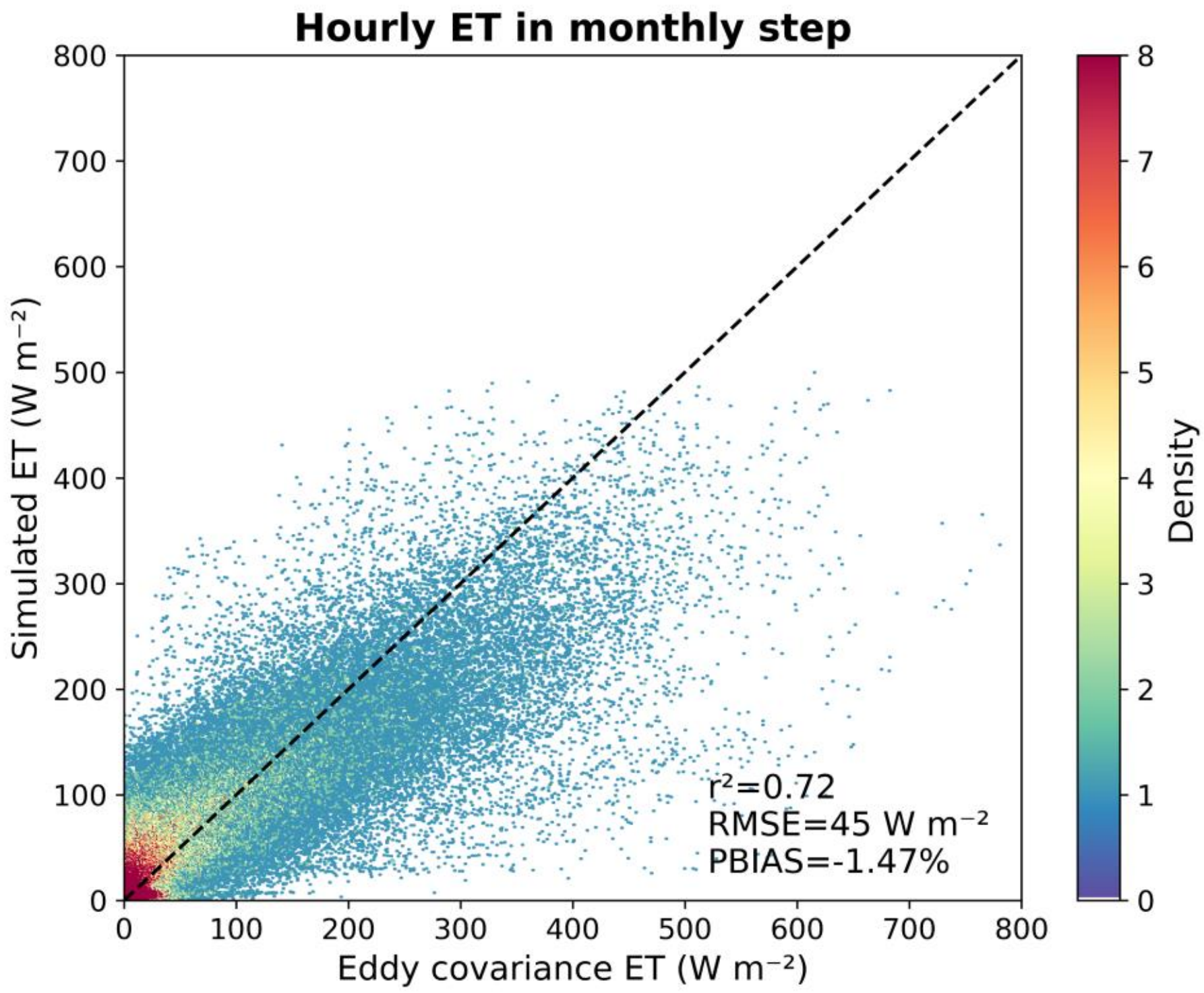

Figure The scatter plots of simulated ET against eddy covariance ET after excluding the first and last years of each site record.

We agree that the scale mismatch between flux footprints and satellite pixels warrants deeper consideration. In the revision, we explicitly evaluated this issue through a land-cover homogeneity–based sensitivity analysis. Please also our response to your comment #1 above for details.

4) Compounding uncertainties: Although corrections have been applied, the complexity of the processes considered may amplify uncertainty. A more rigorous uncertainty quantification is needed.

Response: We agree that ET estimation involves tightly coupled water and energy processes, and that combining corrections across multiple steps can potentially compound uncertainties. In the revised manuscript, we strengthened uncertainty quantification by explicitly characterizing uncertainty at both the model-prediction stage and the EC-based correction stage, and by evaluating their behavior across space, season, and biomes.

First, we quantified predictive uncertainty in the RF-based ECHO-ET estimates using calibrated conformal prediction intervals. This framework is designed to remain valid even under complex, nonlinear model behavior and heterogeneous inputs, because it calibrates uncertainty empirically on an independent calibration subset and was further checked on an independent test set. We report spatially and temporally aggregated uncertainty diagnostics (e.g., prediction interval widths) to demonstrate how uncertainty varies across latitude and season, rather than assuming a constant error level (Fig. S31; Lines 792-822). Please see our response to your comment #1 above.

Second, we explicitly propagated uncertainty in the EC-based correction using an uncertainty-aware calibration framework (Fig. S32, as shown below). We used energy-balance-closure corrected EC ET from the ONEFlux dataset. Measurement uncertainty was explicitly propagated using the joint uncertainty estimate, which integrates both random measurement errors and uncertainties associated with the energy balance closure correction. For sites lacking reliable uncertainty estimates, we adopted a conservative fallback uncertainty defined as 20% of the absolute LE, while preserving missing values. Uncertainty propagation was performed independently for each site using a Monte Carlo approach. For each realization, EC ET values were randomly sampled within their uncertainty bounds (corrected EC ET ± uncertainty), and a regression model was constructed between the sampled EC ET and uncorrected ET. This procedure was repeated 1,000 times, yielding an ensemble of corrected ECHO-ET estimates. The resulting uncertainty showed physically consistent diurnal patterns (low at night and peaking near midday) and systematic differences across vegetation types, indicating that uncertainty is not artificially suppressed by the correction procedure. In the revision, we have added this analysis in the MATERIALS AND METHODS (Lines 823-840) and included the figure in the supplement (Fig. S32).

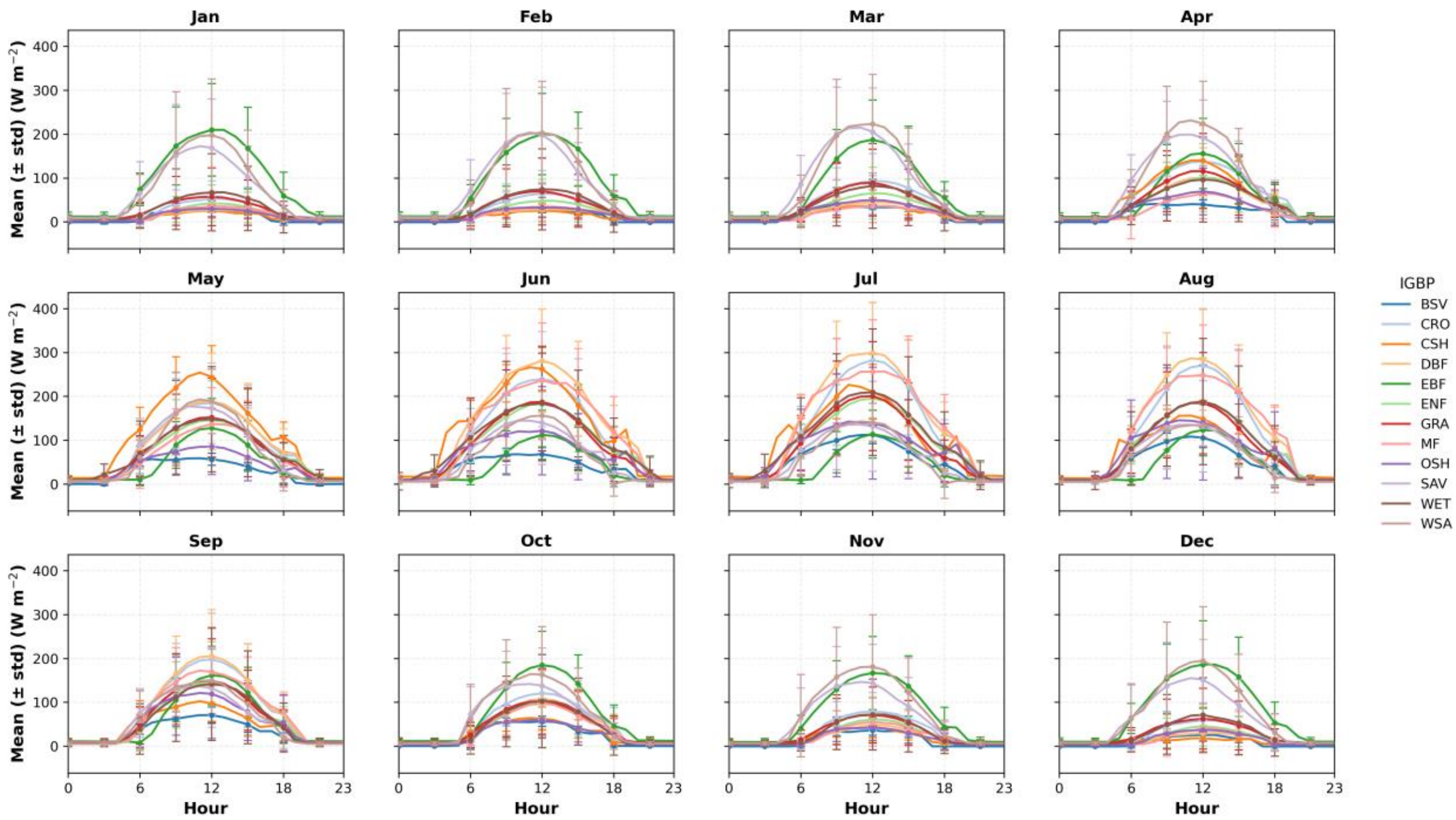

Fig. S32 Monthly diurnal cycles of hourly mean and standard deviation of corrected ECHO-ET across IGBP vegetation types.

Together, these complementary analyses provide a more rigorous and transparent uncertainty quantification for ECHO-ET, and we have expanded the MATERIALS AND METHODS (Lines 792-840) to clearly document the uncertainty framework and its evaluation.

2. Analysis of drought and heatwave impacts

The manuscript analyzes several case studies to examine the influence of drought and heatwaves on diurnal ET cycling. While illustrative, this approach weakens the generality and robustness of the conclusions. What is the global pattern linking droughts/heatwaves to ET cycling? Without broader analyses, the findings remain anecdotal. Temperature is the primary forcing of both droughts and heatwaves. Why not directly assess temperature anomalies instead of focusing only on climatic indices? The analysis currently lacks causal inference. Correlation analyses or causal frameworks are needed to substantiate the claim that droughts and heatwaves alter diurnal ET dynamics.

Response:

Thank you for this constructive and insightful comment. We agree that global-scale illustrations would better support generalized conclusions, and we appreciate the opportunity to clarify and strengthen the analytical framework.

1. From case studies to global patterns

Although the manuscript includes several representative case studies for visualization, our conclusions are not based solely on anecdotal events. In the revised manuscript, we investigated

drought and heatwave impacts using a global, pixel-level analysis across all identified events over the peak growing season within the study period (Fig. 4, as shown below). For each drought month (PDSI ≤ −2.0) and heatwave event (≥7 consecutive extreme-temperature days), we computed event-level anomalies in the diurnal ET centroid relative to near-normal baseline conditions at the same pixel. These anomaly maps were then aggregated globally to quantify spatially coherent patterns. The spatial pattern reveals a broad and coherent advancement of the ET centroid during drought events, with the majority of affected grid cells exhibiting morning shift. These patterns emerge robustly when pooling all events and are not driven by a few illustrative cases. We have described this in the MATERIALS AND METHODS (Lines 947-968) and RESULTS (Lines 225-245).

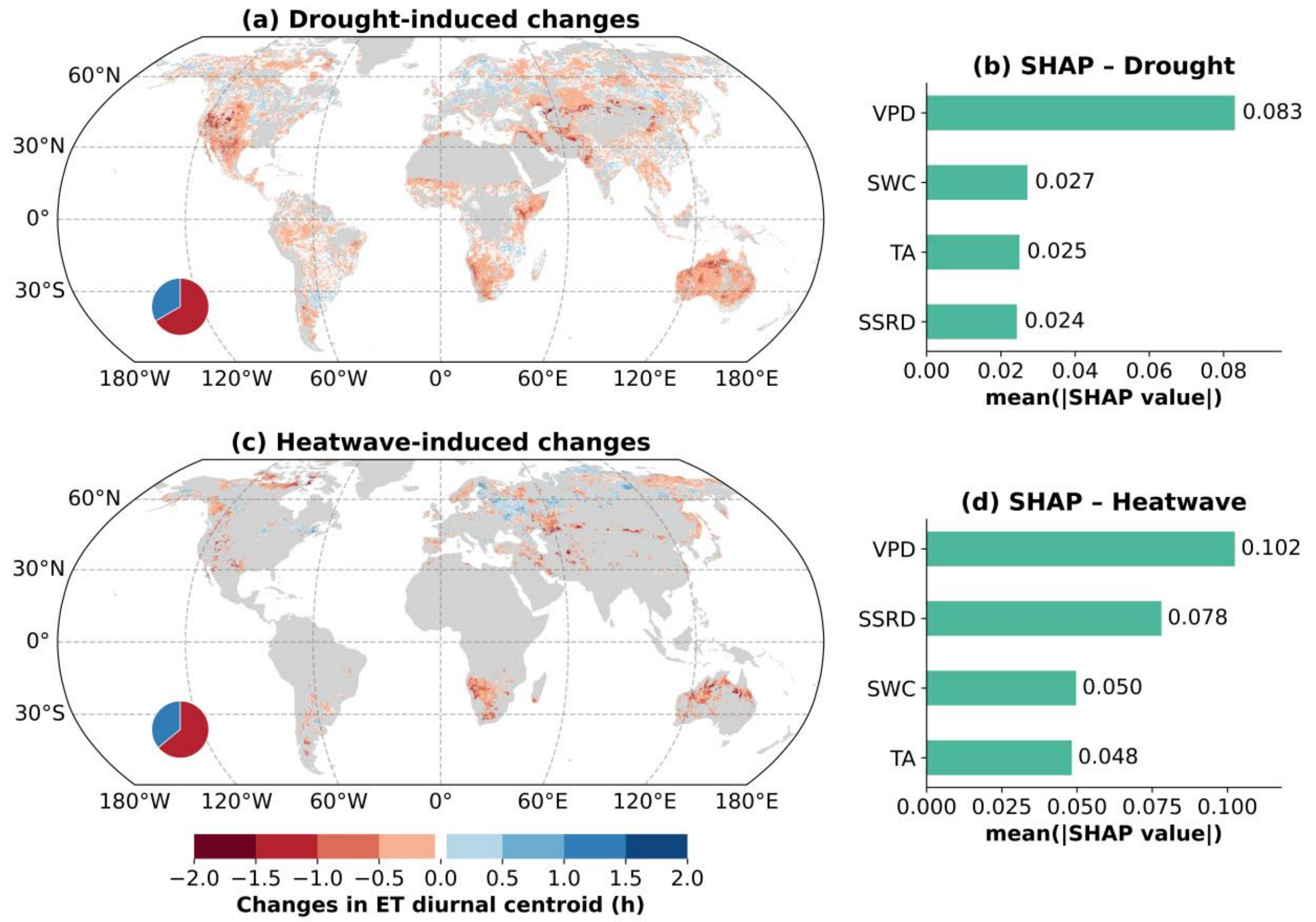

Fig. 4 Global patterns and drivers of drought- and heatwave-induced shifts in diurnal centroid of ET (C_{ET}) during peak-growing season. (a) Global spatial distribution of drought-induced changes in the C_{ET}. C_{ET} anomalies was calculated as the difference between C_{ET} during drought conditions and the corresponding near-normal baseline at the same location. Negative values (red) indicate earlier C_{ET} (advanced ET peak), whereas positive values (blue) indicate delayed timing. (b) Relative importance of environmental drivers explaining drought-induced C_{ET} anomalies, quantified using Shapley Additive Explanations (SHAP) values. Bars represent the mean absolute SHAP value (mean |SHAP|) for vapor pressure deficit (VPD), soil water content (SWC), air temperature (TA), and downward shortwave radiation (SSRD), indicating each variable's overall contribution to model predictions. (c) Global spatial distribution of heatwave-induced changes in C_{ET}. As in (a), negative values denote earlier C_{ET} and positive values denote delayed C_{ET}. (d) SHAP-based attribution of heatwave-induced C_{ET} anomalies. Pie charts in (a) and (c) summarize the global proportion of

pixels exhibiting earlier (red) versus delayed (blue) C_{ET} shifts.

2. Why analyze drought and heatwaves instead of temperature alone?

We agree that temperature is a key forcing of both droughts and heatwaves. However, drought and heatwaves represent fundamentally different stress regimes: drought primarily reflects prolonged soil moisture deficits, while heatwaves represent short-term atmospheric demand extremes and heat stress. Although temperature is central to both phenomena, their ecohydrological impacts differ in mechanism and timescale. For example, heatwaves intensify vapor pressure deficit and energy loading over days, whereas drought alters plant hydraulic status and soil moisture constraints over weeks to months. To address your concern, we expanded our analysis to explicitly examine temperature anomalies alongside soil moisture and radiation anomalies (Fig. 4 and Lines 262-276). This allows us to disentangle whether ET centroid shifts are more strongly associated with atmospheric demand (temperature/VPD anomalies) or hydrologic limitation (soil moisture deficits). The results show that temperature anomalies alone do not fully explain midday depression patterns; rather, the strongest shifts occur when high temperature coincides with low soil moisture, highlighting the role of compound water-energy stress. We have described this in the MATERIALS AND METHODS (Lines 969-987) and RESULTS (Lines 262-276).

3. Toward causal inference to illustrate droughts and heatwaves alter diurnal ET dynamics

We employed a SHapley Additive exPlanations (SHAP)–based attribution framework to quantify the relative contribution of each meteorological driver to ET anomaly shifts (Fig. S9, as shown below). This provides a process-oriented interpretation of how atmospheric demand and water availability jointly regulate diurnal ET dynamics. The attribution results strengthen the inference that droughts and heatwaves systematically alter diurnal ET cycling at global scale. We have described this in the MATERIALS AND METHODS (Lines 969-987) and Results (Fig. 4, S9 and Lines 262-276).

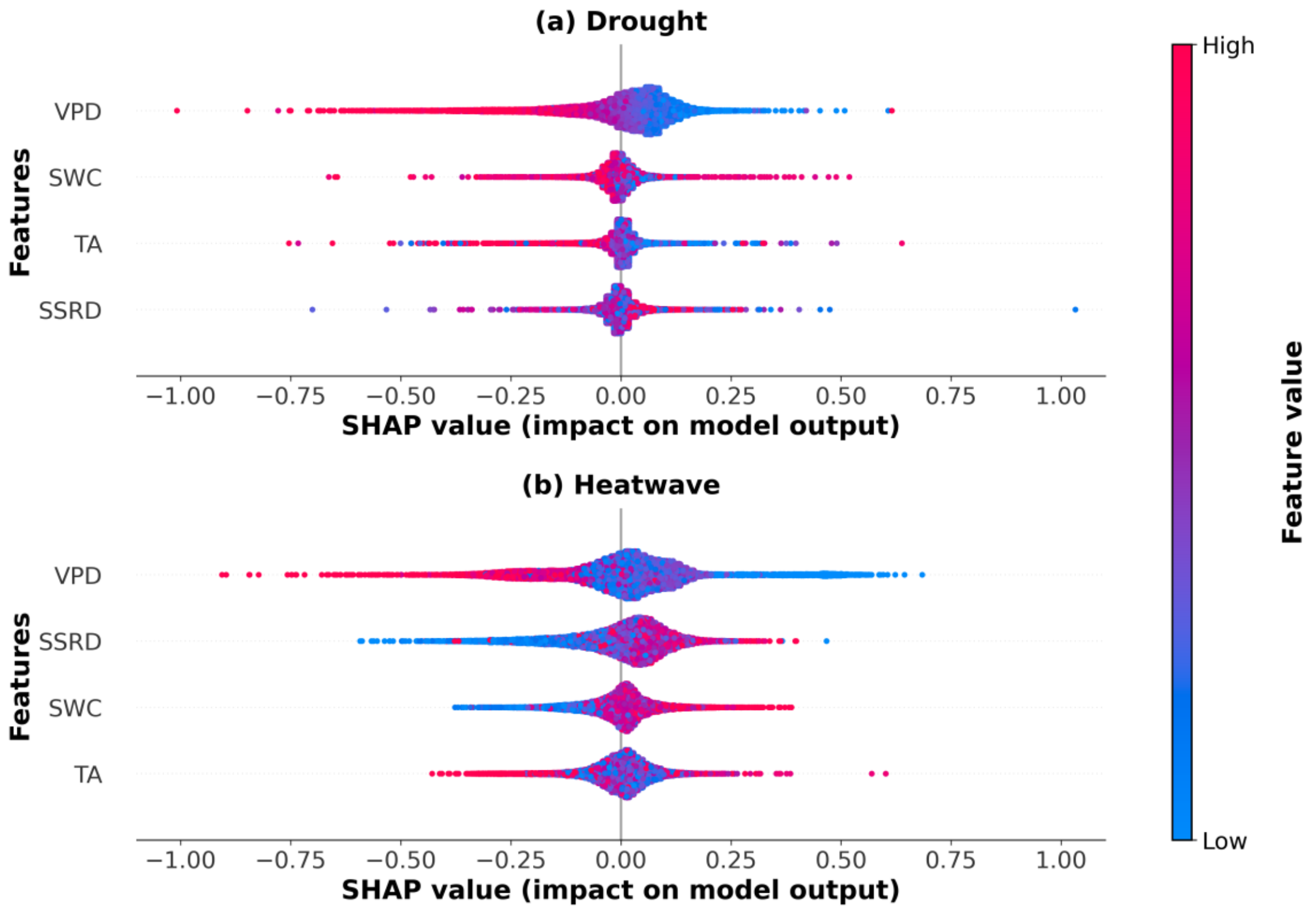

Fig. S9 SHAP-based attribution of vapor pressure deficit (VPD), soil water content (SWC), air temperature (TA), and downward shortwave radiation (SSRD) under (a) drought and (b) heatwave conditions.

We believe these revisions substantially enhance the generality and robustness of our conclusions.

Specific comments:

Line 3. The title equates ET with plant water use, which is inaccurate. ET encompasses both transpiration and evaporation and is more closely related to water-use efficiency. The title should be revised for precision.

Response: We agree that evapotranspiration (ET) is more suitable than plant water use as ET includes both transpiration and evaporation. In the revision, we have changed “plant water use” to “ecosystem evapotranspiration” in the title.

Line 28-29. It is expected that ERA5-L hourly reanalysis may not fully capture midday ET depression. The key question is why: what processes are missing, and what are the implications for improving reanalysis products?

Response: ERA5-L hourly reanalysis, like other global reanalysis products, relies on a combination of observational data, numerical models, and assimilation techniques to estimate environmental variables such as evapotranspiration (ET) at a global scale. However, midday ET depression, which is driven by fine-scale interactions between plant physiology, microclimate, and water availability, may not be fully captured due to several factors, such as

model resolution, stomatal regulation and water availability, and data assimilation process. In the revision, we have discussed this and implications for improving future reanalysis products as follows (Lines 505-520).

"Compared to ECOSTRESS, the coarse resolution FLUXCOM-X and ERA5-Land cannot capture the patterns of diurnal shifts in ET, especially in drylands, which likely reflects limitations in its representation of vegetation water stress. For FLUXCOM-X, which is based on machine learning, a key limitation likely arises from the absence of satellite observations at different times of day; instead, its diurnal variability is primarily driven by hourly meteorological inputs. As a result, it cannot adequately resolve the diurnal dynamics of stomatal conductance, plant water stress, and ET. Similarly, in ERA5-Land, stomatal conductance in land surface schemes is often parameterized as a quasi-steady function of radiation, humidity, and soil moisture, with limited sensitivity to rapid increases in VPD or plant hydraulic constraints. Moreover, the coarse spatial resolution of both products mixes heterogeneous land-cover types and contrasting plant functional responses within a pixel, so averaging across differing diurnal ET patterns tends to smear out the actual sub-daily dynamics. These findings highlight the need for improved representation of plant water stress processes in reanalysis products over the course of the diurnal cycle, including stronger VPD sensitivity of stomatal conductance, dynamic plant hydraulic constraints, and tighter coupling between carbon assimilation and transpiration."

Line 43-83. The extended introduction on flux tower limitations, satellite advantages, and ECOSTRESS features could be shortened. This style is more typical of remote sensing journals and may be excessive for Nature Communications.

Response: Thank you for this helpful comment. We agree that the Introduction should remain concise. However, the discussion of flux tower limitations, satellite-based advantages, and the specific capabilities of ECOSTRESS provides essential context for defining the research gap and clarifying the novelty of our study. In the revision, we have condensed and streamlined these paragraphs while retaining the key arguments necessary to motivate our approach. The total number of words for these paragraphs has been reduced by 20%.

Line 309. Direct comparisons without process-level evaluation are of limited value. While a full mechanistic analysis may be beyond scope, readers would benefit from discussion of why ERA5-L underperforms in this context and how such datasets might be improved.

Response: We agree with you that direct comparisons alone are insufficient and that the manuscript can benefit from a discussion of underlying processes. While a full mechanistic evaluation is beyond the scope of this study, we have expanded the Discussion to clarify why ERA5-Land may underperform in capturing midday ET depression. As we also compared our data with another dataset – FLUXCOM-X in the revision, we also discussed the performance of this additional dataset. Specifically, we note that water-stress controls on stomatal conductance and transpiration—particularly rapid responses to high VPD and plant hydraulic limitation—are likely insufficiently represented in current reanalysis frameworks. We also

discuss implications for improving future reanalysis products (Lines 505-520).

"Compared to ECOSTRESS, the coarse resolution FLUXCOM-X and ERA5-Land cannot capture the patterns of diurnal shifts in ET, especially in drylands, which likely reflects limitations in its representation of vegetation water stress. For FLUXCOM-X, which is based on machine learning, a key limitation likely arises from the absence of satellite observations at different times of day; instead, its diurnal variability is primarily driven by hourly meteorological inputs. As a result, it cannot adequately resolve the diurnal dynamics of stomatal conductance, plant water stress, and ET. Similarly, in ERA5-Land, stomatal conductance in land surface schemes is often parameterized as a quasi-steady function of radiation, humidity, and soil moisture, with limited sensitivity to rapid increases in VPD or plant hydraulic constraints. Moreover, the coarse spatial resolution of both products mixes heterogeneous land-cover types and contrasting plant functional responses within a pixel, so averaging across differing diurnal ET patterns tends to smear out the actual sub-daily dynamics. These findings highlight the need for improved representation of plant water stress processes in reanalysis products over the course of the diurnal cycle, including stronger VPD sensitivity of stomatal conductance, dynamic plant hydraulic constraints, and tighter coupling between carbon assimilation and transpiration."

Reviewer #2 (Remarks to the Author):

General comments to Author:

Review by William M. Hammond, Assistant Professor of Plant Ecophysiology, University of Florida:

This manuscript leverages high-resolution data from ECOSTRESS and ML models to develop high-resolution (in time and space) predictions of ET. This highly original work is significant in its global scale, groundbreaking in its revealing sub-daily patterns of ET (and especially, the timing of ET maxima and depression), and is likely to be of considerable interest across many fields working on land plants, terrestrial ecosystems defined by land plants, and communities working on earth system modeling. Overall I found the manuscript to be well-written, the figures and analyses are compelling, and the conclusions drawn are well supported by the evidence presented. I do have on general comment regarding how plant stomatal responses are framed in the paper, and I think considering this carefully and including the biophysical component (not just active plant responses) would strengthen the manuscript and perhaps also provide a more likely mechanism to point at to support to these primarily globally-synchronous findings regarding midday ET depression. The introduction frames the importance of stomatal closure in "conserving" water (e.g., some stimulus-response is at work; cited literature points to ABA etc.). However, another reason for the midday depression could be more passive—the declining water status (and loss of turgor pressure) in leaves. I think a more rigorous treatment of how and why stomata close in the introduction would strengthen the paper as it can point to the broadly observed biophysical basis for the phenomenon observed. Adding this would also strengthen conclusions drawn regarding the earlier onset of CET during droughts and

heatwaves, as both are associated with lower water content (initially, or through time) in plants, leading to reduced turgor pressure and/or a more rapid acceleration toward closure. References regarding this https://doi.org/10.1111/pce.12774, have found ~80% of stomatal closure can be attributed to the biophysical consequence of reduced turgor, rather than to changes in light, intercellular CO2 concentration, or other factors. As all land plants share stomata that are operated on the basis of turgor pressure, including this framing helps to support the globally cohesive observations, and the observed CET shifts to changing environmental conditions. Overall, I found this paper very exciting to read. The dataset generated will likely fuel many interesting studies on plant-environment interactions, and has profound implications for ongoing and further changes in the Earth system. Below, I include my line-specific comments for the authors to consider.

Response: We thank you for the highly positive evaluation of our manuscript and the thoughtful comments. We are especially grateful for the insightful suggestion regarding the framing of stomatal responses and the underlying biophysical mechanisms of midday ET depression. We have improved our manuscript accordingly. Detailed responses are outlined below.

Line-specific comments:

L19-20: From the opening sentence, the depressing in water use is framed as a plant strategy to limit the loss of water, rather than a constraint on the ability of the plant to sustain maximum water transport. The authors should carefully consider if the evidence supports the current framing.

Response: We have revised the sentence as follows (Lines 20-22) to avoid framing midday depression as an adaptive strategy and instead describe it as a reduction in transpiration associated with both stomatal regulation and hydraulic limitations, consistent with the available evidence.

"Plants often exhibit a midday depression in water use (i.e., transpiration), reflecting a constraint on their ability to sustain maximum water transport, which may occur at the cost of reduced photosynthesis."

L35: Stomata close for more reasons than water conservation, this could be changed "and conserve water upon closure" for example, to highlight what you're emphasizing without making stomatal closure sounding single-purpose.

Response: We have revised the sentence as suggested (Lines 36-37):

"Plants primarily lose water through transpiration via stomata in their leaves, which open to assimilate CO_2 for photosynthesis and conserve water upon closure."

L37-39: By mid-day, it is also more likely that other mechanisms of stomatal closure (e.g., increasing CO2 concentration in the leaf) may take place

Response: We have revised the sentence as follows (Lines 39-42):

"Under environmental stresses, such as high atmospheric demand and water scarcity, typically in the afternoon, plants tend to close stomata, reducing water loss and photosynthesis, with additional influence from increasing internal leaf CO_2 concentration."

We also cited the paper by Rodriguez-Dominguez, C. M., et al. (2016). "Most stomatal closure in woody species under moderate drought can be explained by stomatal responses to leaf turgor." Plant, Cell & Environment 39(9): 2014-2026.

L189: 'vegetative activity' is vague, plants have many active processes that proceed with or without active or maximum ET.

Response: We have modified "vegetative activity" into "key physiological processes such as carbon assimilation and stomatal regulation" (Lines 184-185).

L190: change 'their' to 'its'

Response: Because "vegetative activity" was revised to "key physiological processes such as carbon assimilation and stomatal regulation," the subject is now plural; therefore, the plural pronoun "their" is grammatically appropriate and has been retained (Lines 185).

L200: consider changing 'extended to the left' to 'extended to earlier hours' or something more unit-specific.

Response: Modified (Lines 196-197).

L204-209: This is interesting. If you consider the biophysical constraints on stomatal closure, having a more consistent environment with a more consistent canopy (the EBF tropical forests have on average less variation in canopy area, canopy age, throughout the year than other types investigated), this could be another reason that small differences are observed in EBF. I am not sure there is sufficient evidence to support the current interpretation, "indicating the resilience of rainforests". Resilience would indicate an ability to recover from a non-optimal state. I guess 'tolerance' could fit here, but that doesn't seem quite right either. I interpreted your results to mean that EBF have a more consistent climate (water availability, temperature), thus the supply (water in ground, water in plant tissues), and demand (Atmospheric demand, but also the surface area cross which it is applied!) are more constant. Please consider being more nuanced here than pointing to resilience of rainforests. There is continental-scale evidence that some of Earth's wettest forests may have the narrowest of margins for tolerance and resilience to non-optimal conditions (e.g., as in https://doi.org/10.1111/gcb.15641). Pointing to stability in the CET as an indicator of resilience might build false confidence in these systems, when the CET stability may instead be emerging from not just environmental, but also structural stability in the plants/canopies themselves, which is more typical in EBF than other plant functional types.

Response: We thank the reviewer for highlighting the distinction between resilience and stability. We have revised the interpretation to avoid framing the limited seasonal C_{ET} variability in EBF as evidence of resilience. Instead, we now attribute this pattern to the relatively stable climatic and structural conditions of tropical evergreen forests, which may constrain both water supply and atmospheric demand and lead to more consistent diurnal timing. This interpretation is more consistent with recent continental-scale evidence suggesting that some wet tropical forests may operate close to physiological thresholds under changing environmental conditions. We revised the sentences as follows (Lines 203-207):

"Among these, EBF exhibited the smallest differences in mean and median C_{ET} between the two periods. This muted seasonal variability in EBF likely reflects the relatively stable environmental conditions and canopy structure characteristic of tropical evergreen forests, including limited seasonal variation in water availability, temperature, and effective canopy area."

L226: It would be interesting to include the dominant PFT(s) within each of these regions, noting it even just textually, to link to the prior section.

Response: In the revised manuscript, we added a brief textual description of the dominant PFTs for each region (Lines 284-285).

"Both study regions are primarily water-limited ecosystems dominated by herbaceous plants and shrubs."

L250: Love this figure. Would be clearer if you replaced the _d and _n with clearly labeled "normal years" and "hotter-drought years" or something. This could be done in the legend for lines beneath the last panels, as it's got plenty of room.

Response: Modified (Fig. 6, as shown below).

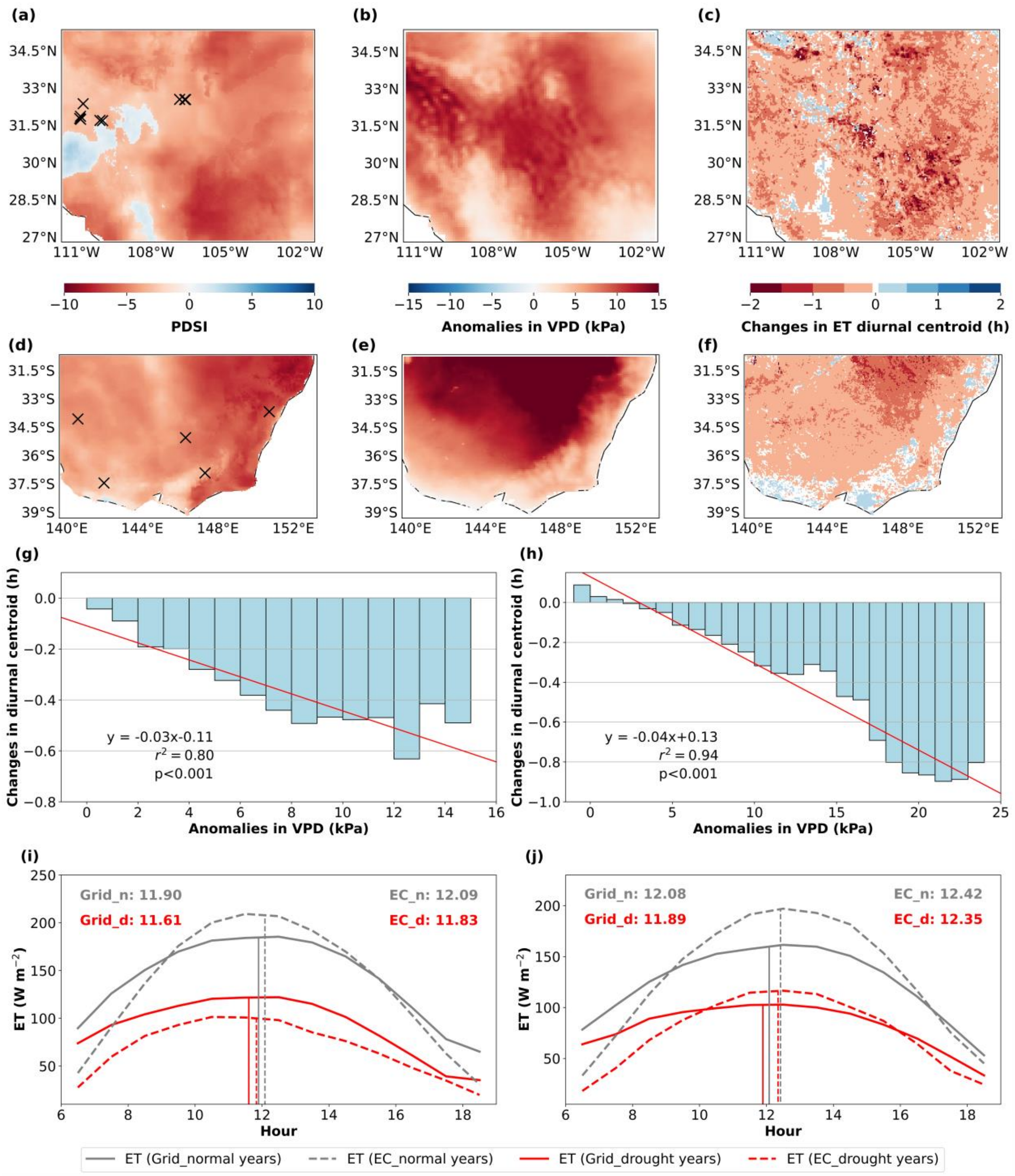

Fig. 6 Effects of drought and heatwave compound events on diurnal variations of evapotranspiration (ET) in (a-c, g, i) southwestern North America during August 2020 and (d-f, h, j) southern Australia during December 2019.

L373: Consider expanding this sentence a bit. For example, stating a more complete picture like …. "Stress results initially in reduced turgor pressure, stomatal closure, and eventually wilting. Under prolonged, severe stress, embolism may form—and eventually lead to death of cells, organs, or whole organisms." Would leave the reader more informed than "this stress results in xylem embolism or leaf wilting". References supporting this sequence would/could include: https://doi.org/10.1038/s41586-018-0240-x; https://doi.org/10.1038/s43017-022-

00272-1; and https://doi.org/10.1111/nph.12798.

Response: In the revised manuscript, we expanded the sentence to describe the progressive nature of plant hydraulic stress and its physiological consequences. Specifically, we now state that water stress initially reduces leaf and guard cell turgor pressure, leading to stomatal closure and suppressed transpiration; with continued stress, loss of turgor can result in visible wilting. Under prolonged and severe conditions, hydraulic failure may occur through xylem embolism formation, potentially leading to cell, organ, or whole-plant mortality. We incorporated the suggested references (e.g., Choat et al., 2018; Delzon et al., 2014; McDowell et al., 2022) to support this mechanistic sequence and to provide a stronger biophysical foundation for interpreting the observed midday ET depression patterns. See more details in Lines 436-438.

"Water stress results initially in reduced turgor pressure and stomatal closure, eventually leading to wilting. Under prolonged or severe deficit, hydraulic failure occurs as xylem embolisms may form, potentially leading to the death of cells, organs, or the entire organisms."

L373-377: Here, the signal-response framing again is dominant, and no mention of the passive biophysical result of lost water at the cell-to-leaf scale, that is a plausible, evidence-supported mechanism for the observed mid-day depression in ET as stomatal turgor cannot be sustained for maximum transpiration; all land plants are subject to these same constraints, where there exist a much greater variety in the biochemical responses/mechanisms that would make it a bit harder to support/explain such a globally cohesive patterning in midday ET dynamics!

Response: We appreciate this suggestion. We revised the text to emphasize passive biophysical constraints on water transport at the cell-to-leaf scale as a primary mechanism underlying midday ET depression, with active stomatal regulation acting as a secondary response within these limits. This framing better reflects the ubiquity of midday depression across land plants and avoids over-attribution to signaling-driven adaptation. See more details in Lines 436-445.

"Water stress results initially in reduced turgor pressure and stomatal closure, eventually leading to wilting. Under prolonged or severe deficit, hydraulic failure occurs as xylem embolisms may form, potentially leading to the death of cells, organs, or the entire organisms. At sub-daily timescales, even moderate imbalances between atmospheric demand and plant water supply can transiently reduce turgor at the cell-to-leaf scale, constraining the ability of stomata to sustain maximum conductance. Although plants possess a range of physiological and biochemical mechanisms to modulate stomatal behavior and mitigate hydraulic risk, these active responses operate within shared biophysical limits on water transport. Consequently, midday depression in ET emerges as a largely passive and ubiquitous outcome of water loss exceeding supply, rather than solely a coordinated, signaling-driven response."

L381: Peaks earlier in the day do avoid additional water loss during increased temperature, but to avoid heat stress, plants must transpire MORE, and not less (to lower leaf surface temperatures, or return the living cells to thermal optima for biochemical processes). Consider re-phrasing this to add in some of this important nuance.

Response: Thank you for this insightful comment. We agree that avoidance of heat stress does not necessarily imply reduced transpiration; on the contrary, evaporative cooling through transpiration is a key mechanism by which plants regulate leaf temperature and maintain favorable thermal conditions for biochemical processes. We have therefore revised the text to remove the implication that earlier diurnal peaks arise from an active strategy to avoid heat stress. Instead, we now frame the earlier peak in vegetation activity as a consequence of increasing atmospheric demand and water limitation later in the day, which can constrain physiological processes despite the cooling benefits of transpiration. This revision better reflects the inherent trade-off between water loss and thermal regulation under hot and dry conditions. See more details in Lines 448-451.

"Vegetation activity is susceptible to environmental stresses during summer, particularly in drylands, and often peaks earlier in the day as increasing atmospheric demand later in the day constrains physiological processes, despite the role of transpiration in alleviating heat stress."

L397: This is great. Consider adding the information that when comparing within the same location, across time, that midday depression is a stress indicator; comparing between sites this may not always strictly speaking be true (I don't think the paper currently asserts this, obviously for a variety of reasons the CET may be different due to plant structural, community compositional, dominant photosynthetic pathway, or other factors).

Response: We agree that midday depression should be interpreted differently when comparing temporal changes within the same location versus spatial differences across sites. We have revised the sentence in Lines 465 and 478-480.

L405-407: Agreed! The utility of midday depression as a remotely-sensed signal from the plant (not the environment) is the reason I was most excited to read and review this paper.

Response: Thank you very much for this encouraging comment. We are delighted that the interpretation of midday depression as a remotely sensed plant signal resonated with you.

L419: It remains unknown if under extreme heat waves, if plants will keep their stomata closed, or not. Whether this is driven biochemically or biophysically (as in the 'wrong-way response'), is uncertain. The methods developed her may help to investigate these emerging phenomenon, as heat extremes that are novel in the current era are emerging more frequently, for example the 2021 PNW heat dome, where heat caused significant damages. It is unclear if running out of water (as proposed in: https://doi.org/10.1093/treephys/tpac007) or getting too hot (as we proposed: https://doi.org/10.1093/treephys/tpac143) were dominant mechanisms of ecosystem-scale canopy die-back. Having a means to monitor sub-daily ET will be a fantastic tool to study these phenomena, and even to understand the mechanisms that predominate during such extreme events.

Response: Thank you for the affirmative comment on the importance of our study. We agree that under extreme heatwaves it remains uncertain whether plants will maintain stomatal

closure, partially reopen, or become constrained by hydraulic or thermal limits, which is still actively debated.

In the revised manuscript, we expanded this section to better acknowledge these mechanistic uncertainties. We now clarify that while midday ET depression is often interpreted as a signature of stomatal regulation under stress, the underlying drivers may involve both passive hydraulic constraints and temperature-induced physiological or structural limits. Importantly, we emphasize that the framework developed here—providing globally consistent, sub-daily ET dynamics—offers a powerful observational tool to investigate such emerging phenomena. By tracking shifts in the timing and magnitude of midday depression during extreme events, ECHO-ET can help diagnose whether ecosystems primarily exhibit hydraulic limitation, thermal constraint, or combined stress responses. We have revised the text accordingly to better highlight this forward-looking application and to position our dataset as a means to advance understanding of vegetation resilience under increasingly frequent climate extremes (Lines 488-500).

We appreciate this suggestion, as it strengthens the discussion and better situates our findings within ongoing debates about plant responses to extreme heat and drought.

Reviewer #3 (Remarks to the Author):

The manuscript presents an important study on quantifying the global midday depression in plant evapotranspiration (ET) using ECOSTRESS observations and machine learning. The topic is timely and relevant, especially in the context of plant water stress under drought and heatwave conditions. However, there are several critical issues that need to be addressed before the manuscript can be considered for publication:

Response: We sincerely thank you for positive evaluation of our manuscript and thoughtful feedback and valuable comments. These constructive comments have helped us significantly improve the quality of our manuscript. Detailed responses are provided below.

1. The manuscript repeatedly refers to "machine learning" in general terms. For reproducibility and clarity, the authors should explicitly state the specific algorithm(s) employed and provide details on model training, feature selection, and validation.

Response: Thank you for this suggestion. In the original manuscript, we specified the machine learning approach — random forest, described training procedure, predictor handling, and validation strategy, and reported the associated performance metrics in the Methods. In the revision, we have specified the specific machine learning method in the Introduction (Line 82) and also revised the Methods to provide sufficient methodological detail for reproducibility (Lines 676-680).

"We used a machine learning method, the Random Forest (RF) algorithm, to develop the predictive ET model. The model consisted of 100 trees and minimized mean squared error. All

predictor variables were included without prior feature elimination, allowing the model to account for nonlinear relationships and feature interactions. Bootstrap aggregation was applied during training, with no constraint on tree depth and a minimum leaf size of one."

2. While the study highlights the advantages of ECOSTRESS, a comparative evaluation using other global ET products (e.g., MOD16, FLUXCOM, GLEAM, ERA5-Land) is necessary. Such comparisons would help demonstrate the added value of ECOSTRESS and contextualize the findings against existing datasets.

Response: Thank you for this important suggestion. We agree that situating ECHO-ET within the context of existing global ET products is essential for demonstrating its added value. In the original manuscript, we compared our dataset with another global ET product (ERA5-Land), demonstrating the unique contribution of ECOSTRESS-based hourly ET in advancing understanding of diurnal plant water-use dynamics (Fig. 8, as shown below).

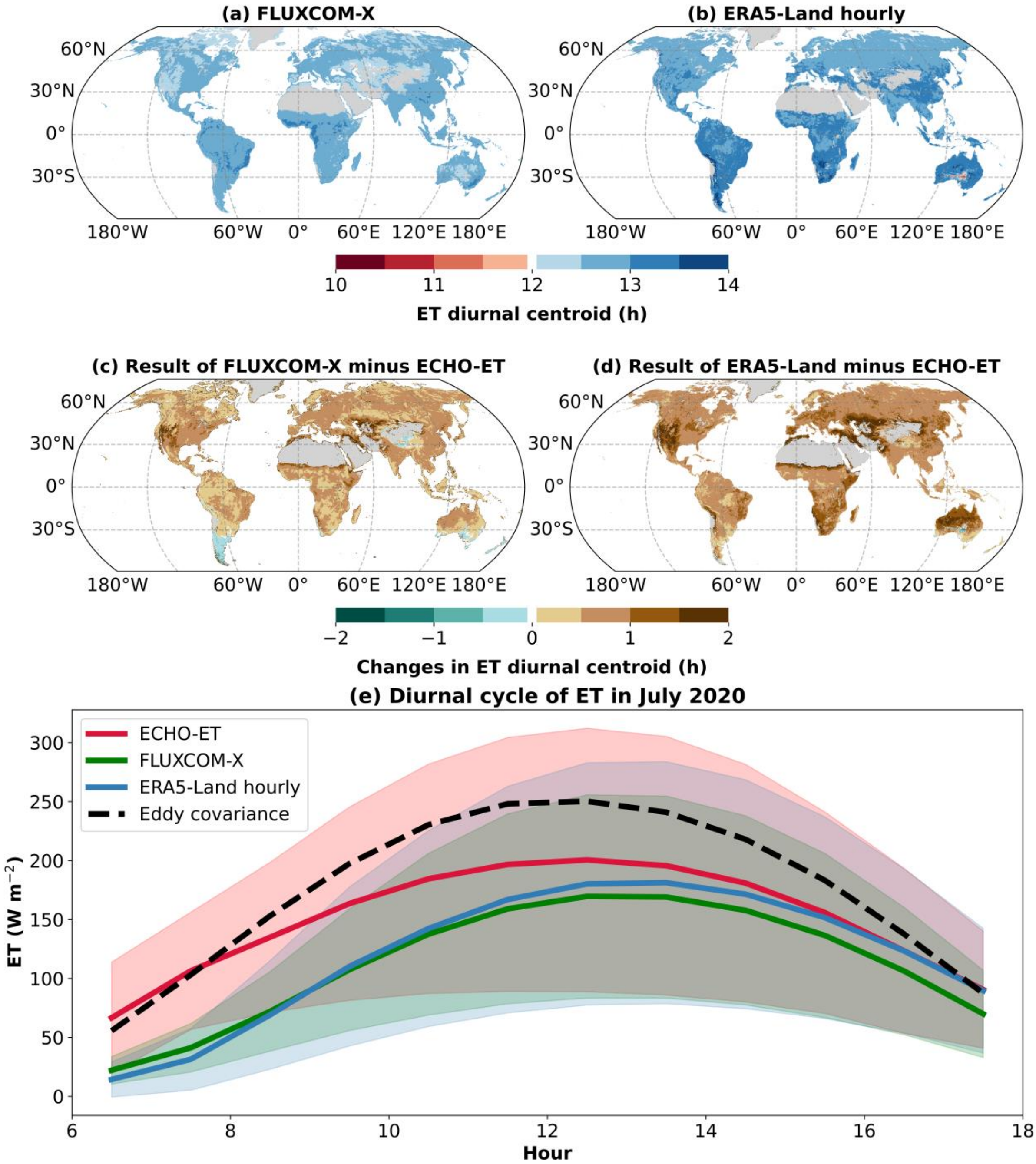

Fig. 8 Diurnal variation of global hourly ET in July 2020 derived from FLUXCOM-X and ERA5-Land. Compared to ECHO-ET and eddy covariance (EC) observations, FLUXCOM-X and ERA5-Land substantially overestimated ET in the afternoon. (a) Global diurnal centroid in ET (CET) from FLUXCOM-X. (b) Global CET from ERA5-Land. (c) Difference in CET between FLUXCOM-X and ECHO-ET. (d) Difference in CET between ERA5-Land and ECHO-ET. (c) Diurnal cycling of ET from ECHO-ET, FLUXCOM-X, ERA5-Land, and EC. The shaded area represents the range of error.

In the revised manuscript, we have expanded the comparative analysis against another global hourly ET dataset — FLUXCOM. MOD16 and GLEAM are satellite-based products at daily or longer timescales and are not capable of diagnosing sub-daily ET dynamics. The comparison

focuses not only on mean ET magnitudes, but also on sub-daily behavior and the timing of ET peaks, which is central to this study.

The results showed that in terms of ET magnitude, ECHO-ET exhibits strong positive agreement with both FLUXCOM-X (r^2 = 0.85, RMSE = 34 W m^{-2}) and ERA5-Land hourly (r^2 = 0.80, RMSE = 36 W m^{-2}) at the monthly-averaged diurnal scale, with slightly better consistency observed for FLUXCOM-X (Fig. S29, as shown below).

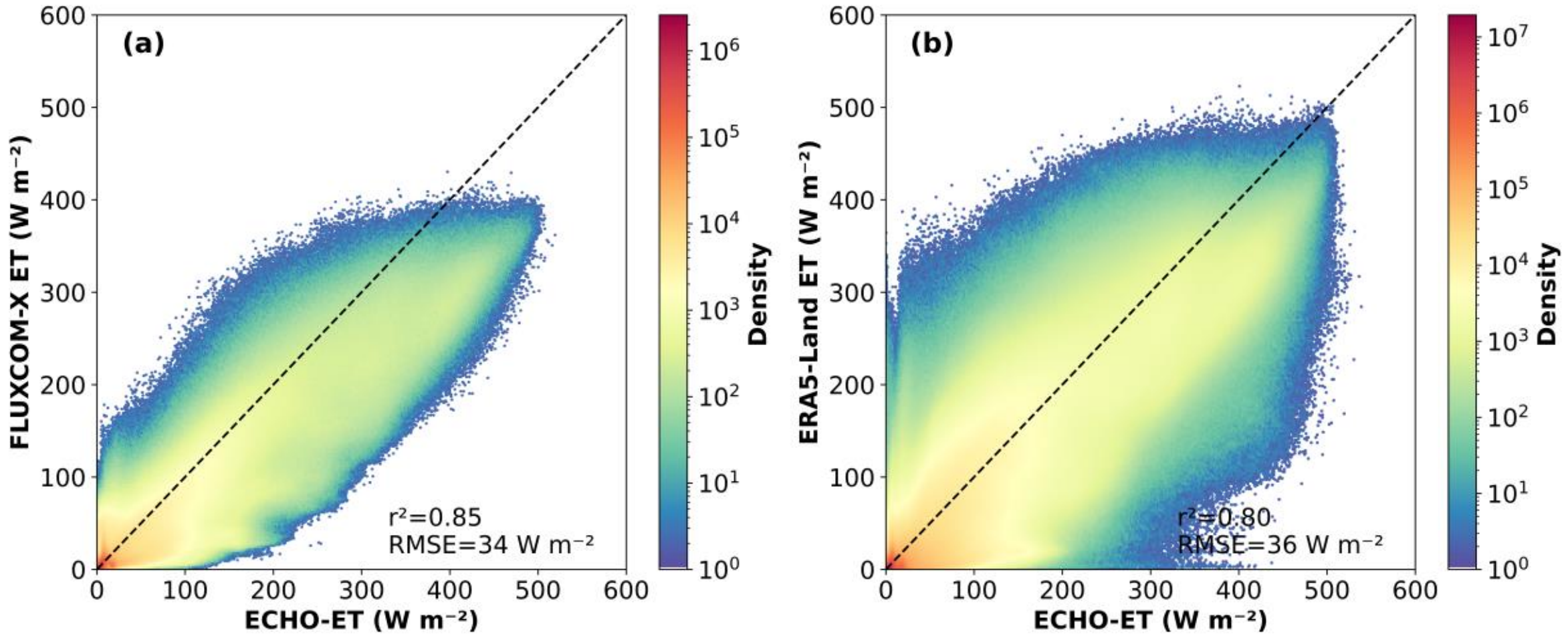

Fig. S29 The scatter plots of (a) FLUXCOM-X and (b) ERA5-Land ET against ECHO-ET at monthly-average diurnal scale.

We then compared the diurnal ET cycle from ECHO-ET with that of FLUXCOM-X. Across vegetated regions, ECHO-ET showed an earlier ET centroid closely matching EC observations, whereas FLUXCOM-X exhibited a slight delay and ERA5-Land showed a pronounced afternoon lag (up to ~1-2 hours), consistently overestimating afternoon dominance across months. ECHO-ET uniquely resolves sub-daily variability and systematically identifies earlier ET centroids under drought and heatwave conditions. In contrast, reanalysis-driven products such as ERA5-Land tend to produce smoother diurnal cycles and often underestimate the magnitude of midday depression, particularly in water-limited regions.

We have clarified in the manuscript that the added value of ECOSTRESS lies not in altering large-scale annual ET totals, but in enabling global, observation-constrained analysis of sub-daily ET timing and stress responses—an aspect not directly accessible from existing global datasets. This distinction helps contextualize our findings and demonstrates that ECHO-ET complements, rather than replaces, current global ET products.

Please see Fig. 8, Fig. S12-S16, Fig. S29 as well as Lines 363-382 and 735-740 for more details.

3. ECOSTRESS data is limited to latitudes between ~52°N and 52°S. The manuscript should discuss the implications of this coverage limitation, including potential uncertainties or biases in regions outside the covered area. The authors should also clarify whether the machine learning approach extrapolates to higher latitudes, and if so, how reliable such extrapolations are.

Response: We agree that the latitudinal coverage limitation of ECOSTRESS (approximately 52°N-52°S) warrants explicit discussion, particularly regarding model extrapolation and uncertainty at higher latitudes.

To evaluate extrapolation performance, in the original manuscript, we conducted an independent comparison against 28 flux tower sites located in high-latitude regions beyond the nominal ECOSTRESS coverage. Despite the lack of direct ECOSTRESS observations in these areas, the model remained reliable, achieving $R^2 = 0.63$ and RMSE = 31 W m^{-2} (Fig. S26, as shown below). The diurnal centroid of ET derived from the gridded ET data closely matched that at EC flux sites, including the above 60°N region where the ECOSTRESS cannot cover (Fig. S35, as shown below). See Lines 726-729 and 903-905 for more details.

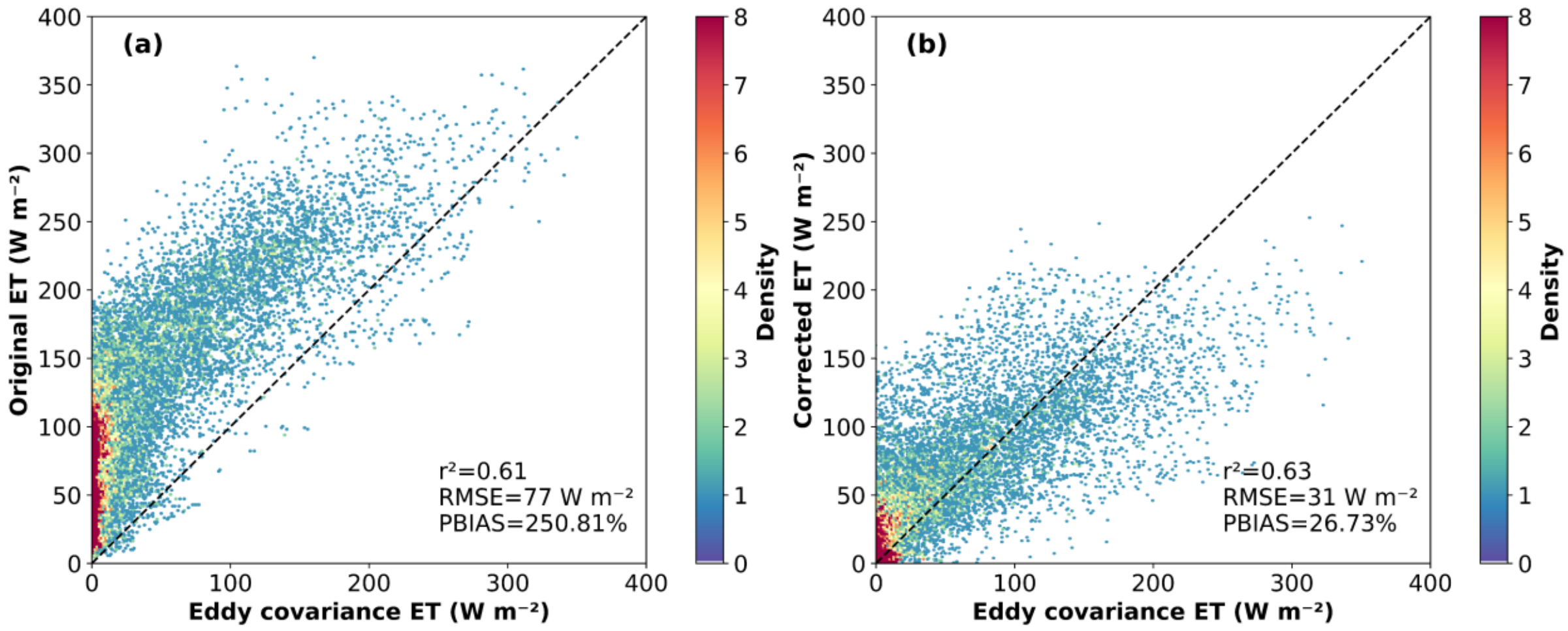

Fig. S26 The scatter plots of simulated ET against eddy covariance ET in high-latitude regions (north of 52°N) where ECOSTRESS coverage is absent: (a) before correction and (b) after correction.

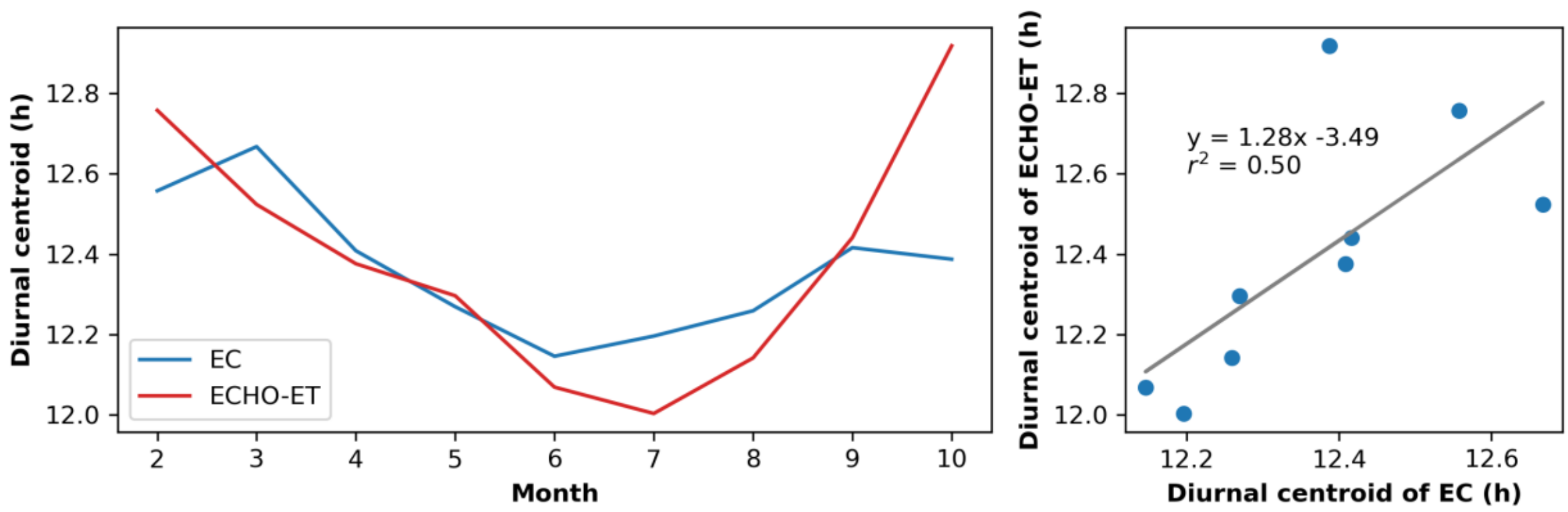

Fig. S35 Seasonal and correlation analysis of the diurnal centroid for ET (C_{ET}) above 60°N, derived from 23 eddy-covariance (EC) sites and ECHO-ET respectively.

In the revised manuscript, we further strengthened this assessment by explicitly examining predictive uncertainty across latitudinal gradients. our uncertainty analysis showed that high-latitude regions did not exhibit systematically larger prediction intervals compared to mid-latitude or semi-arid regions (Fig. S31, as shown below). The absence of amplified uncertainty

in these zones suggests that reduced ECOSTRESS observational coverage did not propagate into inflated predictive uncertainty in the global product. See Lines 818-822 for more details.

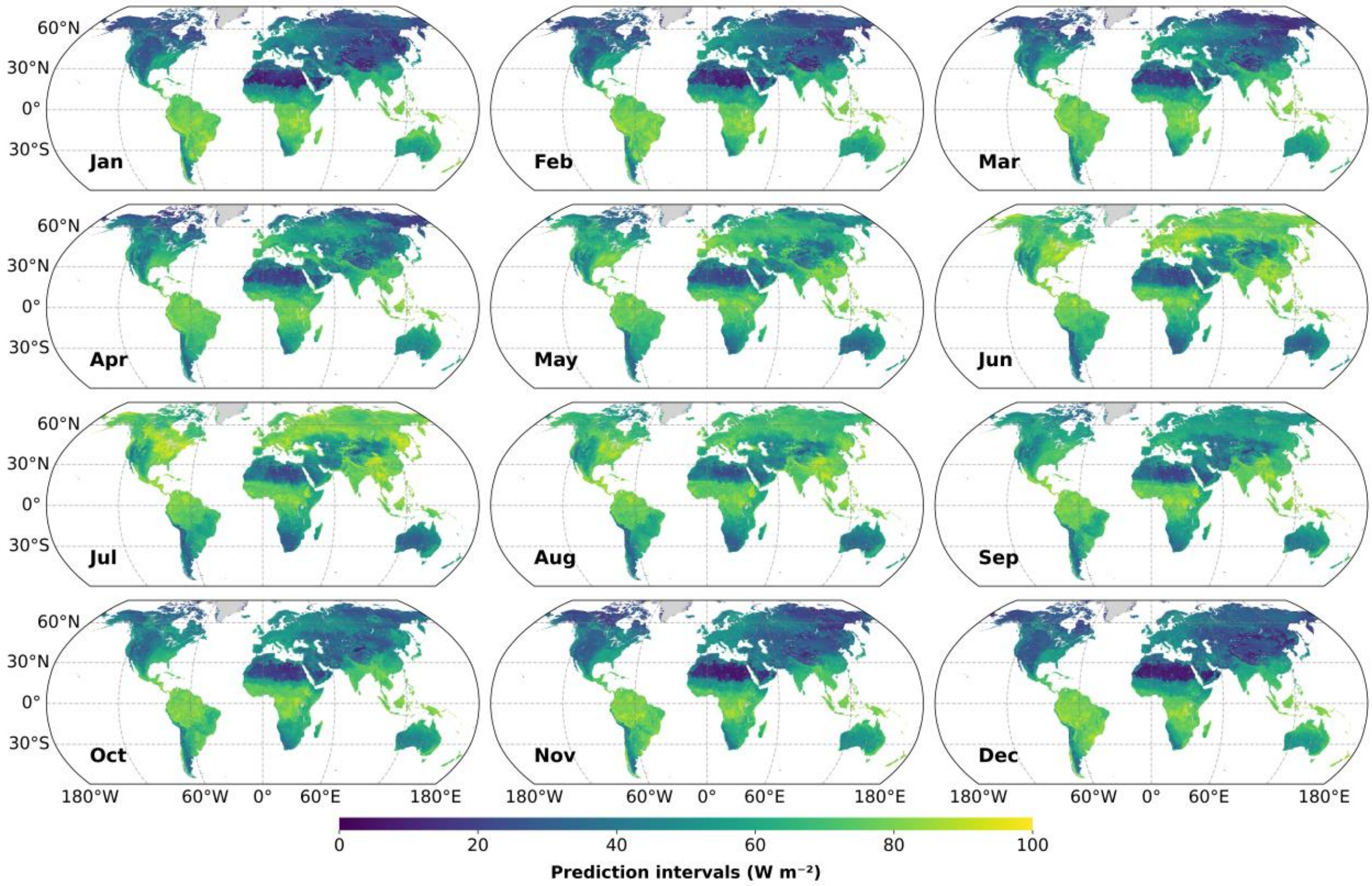

Fig. S31 Global patterns of daily-average hourly prediction intervals (PIs) for the Random Forest-based ET estimates.

In the manuscript, we explicitly acknowledge the ECOSTRESS coverage limitation, clarify the extrapolation framework, and present additional validation and uncertainty diagnostics for high-latitude regions. While we recognize that any spatial extrapolation inherently introduces additional uncertainty, independent tower validation and calibrated prediction intervals indicate that the model remains robust beyond the nominal ECOSTRESS observational domain. Importantly, water stress is generally less pronounced at high latitudes due to lower atmospheric demand and greater soil moisture availability, reducing the likelihood of strong water-limitation-driven responses. This ecological context further supports the stability of model performance in these regions.

4. Include uncertainty estimates for the machine learning ET predictions.

Response: Thank you for this important comment. In the revised manuscript, we have included uncertainty estimates for our ECHO-ET predictions. Please see our response to Reviewer 1's Comment #1 for details.

5. Clarify the relationship between observed midday depression and shifts in peak ET (CET) across different climatic zones.

Response: We agree that explicitly linking midday depression to shifts in the diurnal centroid

of ET across climate zones strengthens the interpretability of the results.

The shift in centroid of evapotranspiration (C_{ET}) arises directly from changes in the temporal distribution of ET within the diurnal cycle. C_{ET} is defined as the ET-weighted mean time of day; therefore, any asymmetric suppression of ET during the afternoon reduces the relative contribution of later hours to the daily integral, effectively shifting the centroid earlier. Fig. S8 (as shown below) shows that C_{ET} varies systematically across Plant Functional Types (PFTs) and Köppen climate zones, with both convergence and divergence patterns emerging under different environmental constraints. Fig. 3 (as shown below) further clarifies this climate-dependent behavior by stratifying regions into energy-limited and water-limited regimes within temperate and tropical zones. The Kolmogorov–Smirnov statistics and Cohen's d effect sizes confirm that these C_{ET} distribution differences between energy- and water-limited regimes are statistically significant. Taken together, these results demonstrate that observed midday depression corresponds directly to systematic shifts in C_{ET}, and that the magnitude and timing of these shifts depend on climatic water–energy balance. This climate-stratified analysis provides a mechanistic interpretation of how hydroclimatic regime modulates the timing of peak ET globally.

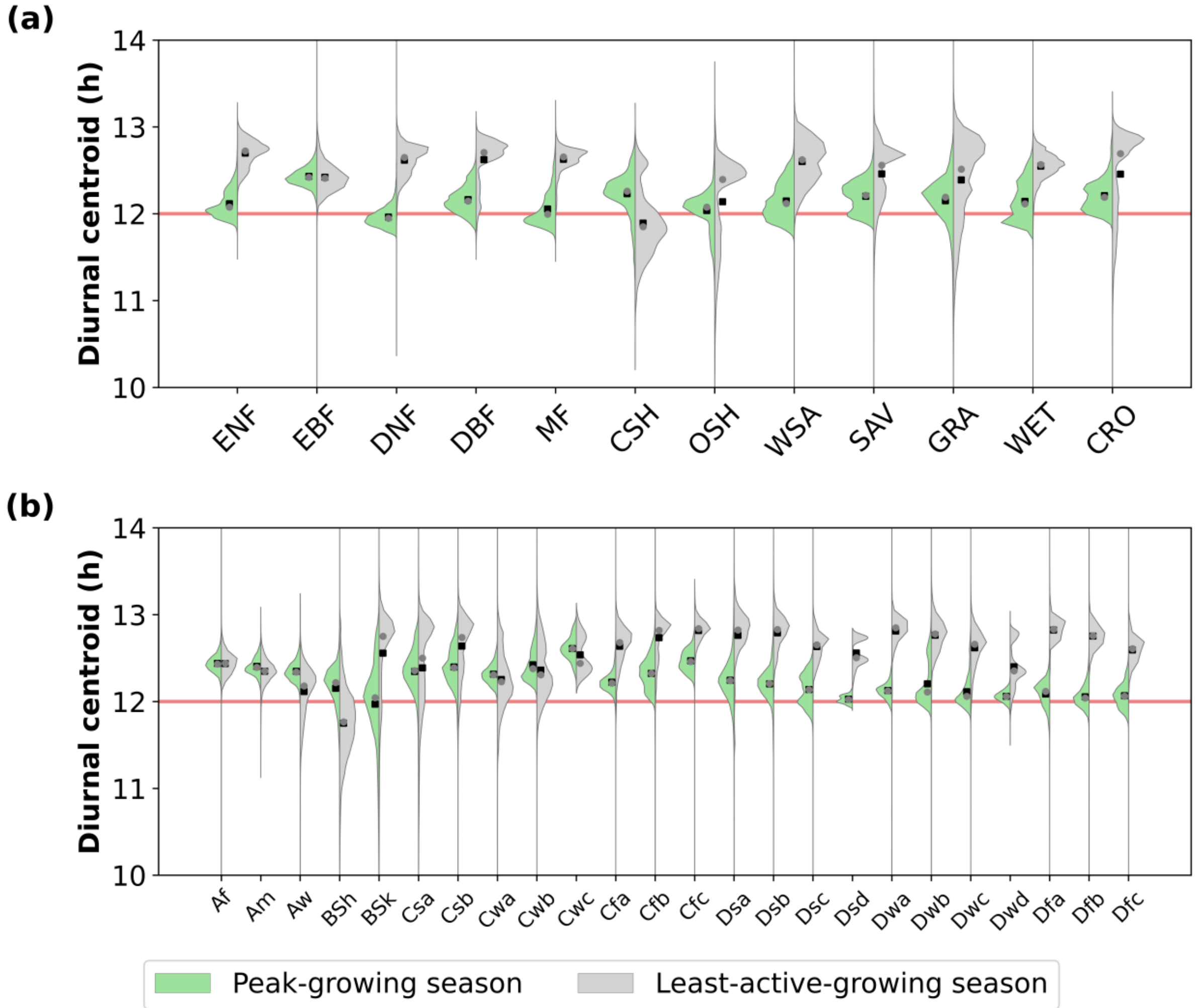

Fig. S8 Convergence and divergence in diurnal variation of ET across Plant Functional Types (PFTs) in different climate zones, and mean (black rectangles) and median (grey circles) values are also reported, (a) PFTs, (b)Koppen climate categories.

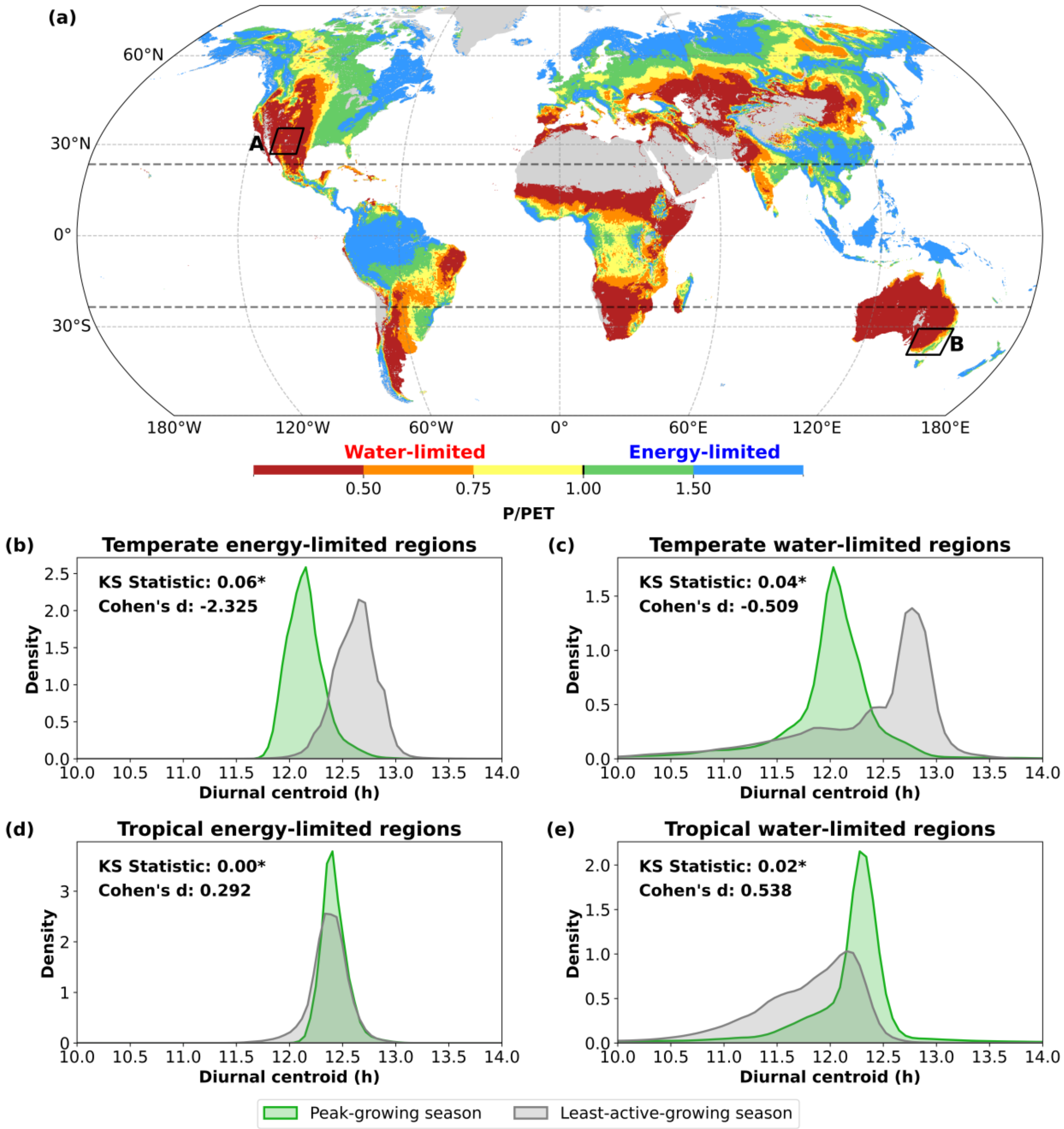

Fig. 3 Convergence and divergence in diurnal variations in evapotranspiration (ET) across environmental conditions.

Please see Lines 173-209 for more details.

6. Consider discussing the temporal resolution of ECOSTRESS and its implications for capturing diurnal ET dynamics.

Response: Thank you for this important comment. We agree that the temporal sampling of ECOSTRESS is central to interpreting its ability to resolve diurnal ET dynamics.

Although ECOSTRESS does not provide continuous hourly observations at each location, its precessing orbit results in varying local overpass times, allowing different portions of the diurnal cycle to be sampled over successive days. In our original manuscript, by integrating these temporally distributed snapshots within a machine learning framework, we developed continuous hourly ET estimates at the global scale and named this dataset ECHO-ET. Validation against eddy covariance observations demonstrates that the resulting diurnal cycles, including peak timing and midday depression, are physically realistic and robust (see Lines 640-740).

In the revised manuscript, we further confirm that by resolving sub-daily ET dynamics at global scale, ECHO-ET captures shifts in the timing and magnitude of midday depression, thereby providing a direct and physiologically meaningful indicator of plant water stress beyond traditional meteorological drought metrics (see Fig. 4 as shown below; Lines 225-245).

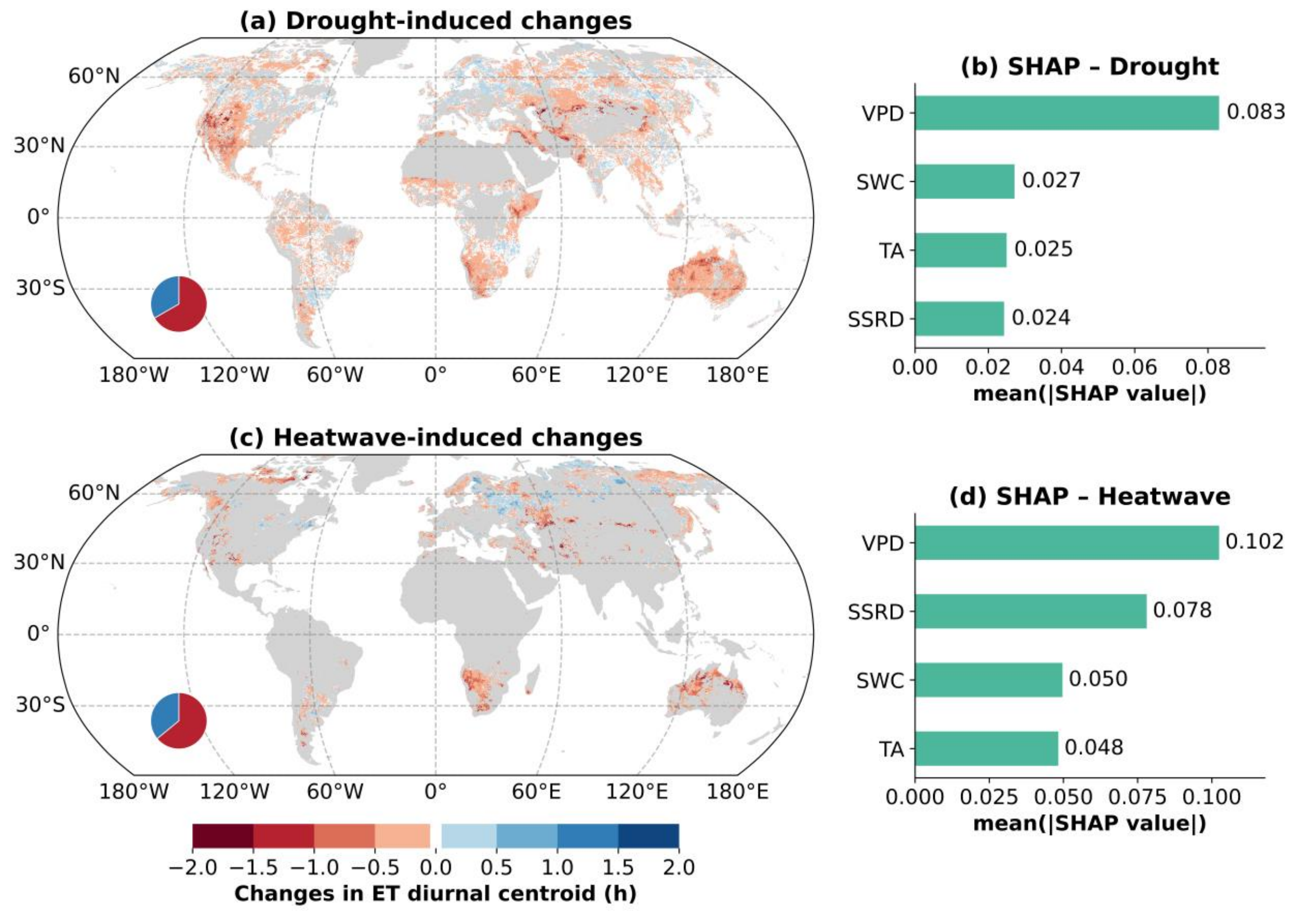

Fig. 4 Global patterns and drivers of drought- and heatwave-induced shifts in diurnal centroid of ET (C_{ET}) during peak-growing season. (a) Global spatial distribution of drought-induced changes in the C_{ET}. C_{ET} anomalies was calculated as the difference between C_{ET} during drought conditions and the corresponding near-normal baseline at the same location. Negative values (red) indicate earlier C_{ET} (advanced ET peak), whereas positive values (blue) indicate delayed timing. (b) Relative importance of environmental drivers explaining drought-induced C_{ET} anomalies, quantified using Shapley Additive Explanations (SHAP)

values. Bars represent the mean absolute SHAP value (mean |SHAP|) for vapor pressure deficit (VPD), soil water content (SWC), air temperature (TA), and downward shortwave radiation (SSRD), indicating each variable's overall contribution to model predictions. (c) Global spatial distribution of heatwave-induced changes in C_{ET}. As in (a), negative values denote earlier C_{ET} and positive values denote delayed C_{ET}. (d) SHAP-based attribution of heatwave-induced C_{ET} anomalies. Pie charts in (a) and (c) summarize the global proportion of pixels exhibiting earlier (red) versus delayed (blue) C_{ET} shifts.

We have clarified this point to emphasize that while instantaneous coverage of ECOSTRESS snapshots is sparse, our ECHO-ET dataset developed with a machine learning framework enables reliable characterization of diurnal ET dynamics and detecting global diurnal water stress. Please see Lines 396-410 and 481-523 for more details.

Responses to Reviewers' Comments

Review round: 2

Reviewer #1 (Remarks to the Author):

Thanks for your great work in the revision. My comments have been well addressed. I have no further questions.

Response: We sincerely thank the reviewer for the positive evaluation of our revised manuscript and for acknowledging that the comments have been well addressed. We greatly appreciate the reviewer's earlier constructive and insightful feedback, which significantly helped improve the quality and clarity of our work.

Reviewer #2 (Remarks to the Author)

PEER REVIEW for "NASA's ECOSTRESS satellite reveals widespread midday depression in ecosystem evapotranspiration" by Bu et al., submitted to Nature Commuunications
Peer review by William M. Hammond

General comments:
The new analysis considering the impacts of drought and heatwaves on ET is novel and provides another clear example of the utility of the global ECHO-ET dataset. By adding the analysis for heatwave stress, the authors have revealed that compound "hotter droughts" are most responsible for midday ET depression; this builds globally on a site- and plot-based series of observations that hotter-drought is driving plant stress and mortality globally in forests, for example. The authors could connect this new robust analysis to recent literature on these topics, pointing out that evidence from multiple approaches and scales are pointing towards the same conclusion: warming, especially atop drought stress, is increasingly challenging the vital role of plants in ecosystems.

The authors have carefully re-frame their interpretation of plant water use to include a wider array of evidence (e.g., biophysical mechanisms, intracellular CO_2 concentrations, and active hormonal controls. In this revision, the authors have sufficiently addressed my concerns with the prior version of the manuscript. In particular, the addition of an impacts of drought and heatwaves on diurnal ET cycles adds important context, and builds confidence in the ECHO-ET product. I expect that its contribution of the new ECHO-ET dataset, and the knowledge revealed here in its first application, will both be of high impact and interest to the readers of Nature Communications. A few line-specific comments follow, aimed at improving clarity of the manuscript and its figures.

Response: We sincerely thank the reviewer for the positive evaluation of our revised manuscript and for recognizing the novelty and utility of the new drought and heatwave

analysis. We also thank the reviewer for his earlier constructive and insightful comments that significantly helped us improve our manuscript. Detailed responses are outlined below.

Line-specific comments:
L33: change ‘uses’ to ‘use’

Response: Modified (Lines 34).

L71: add a space between ‘the’ and ‘1’

Response: Modified (Lines 71).

L77-78: This could be clearer, something like “While covering a broad portion of Earth’s land surface, earlier …(the existing sentence, without ‘also’).”

Response: We thank the reviewer for this helpful suggestion. We have revised the sentence as suggested (Lines 77-78).

L274-276: This is an excellent contribution to our knowledge of drought vs. heatwave impacts, at such a broad scale. Well done!

Response: We sincerely thank the reviewer for this positive comment. We are pleased that the reviewer found this broad-scale analysis of drought versus heatwave impacts valuable. This new analysis has helped us better clarify the role of compound hot-drought stress in shaping midday ET depression.

L304-324: Fig. 6: Clearer legend labels at bottom of this figure. Are you sure this is ‘drought years’ and not ‘hotter drought’ years, based on the new analyses and importance of warming atop drought in driving observations? Even if the analyses were filtered for drought, did you check that these years were “average” in temperature” if they are warmer than long-term, please clearly label as hotter-drought or similar to provide a fuller picture of the difference in drivers between normal and anomalous years.

Response: Modified (Fig. 6, as shown below).

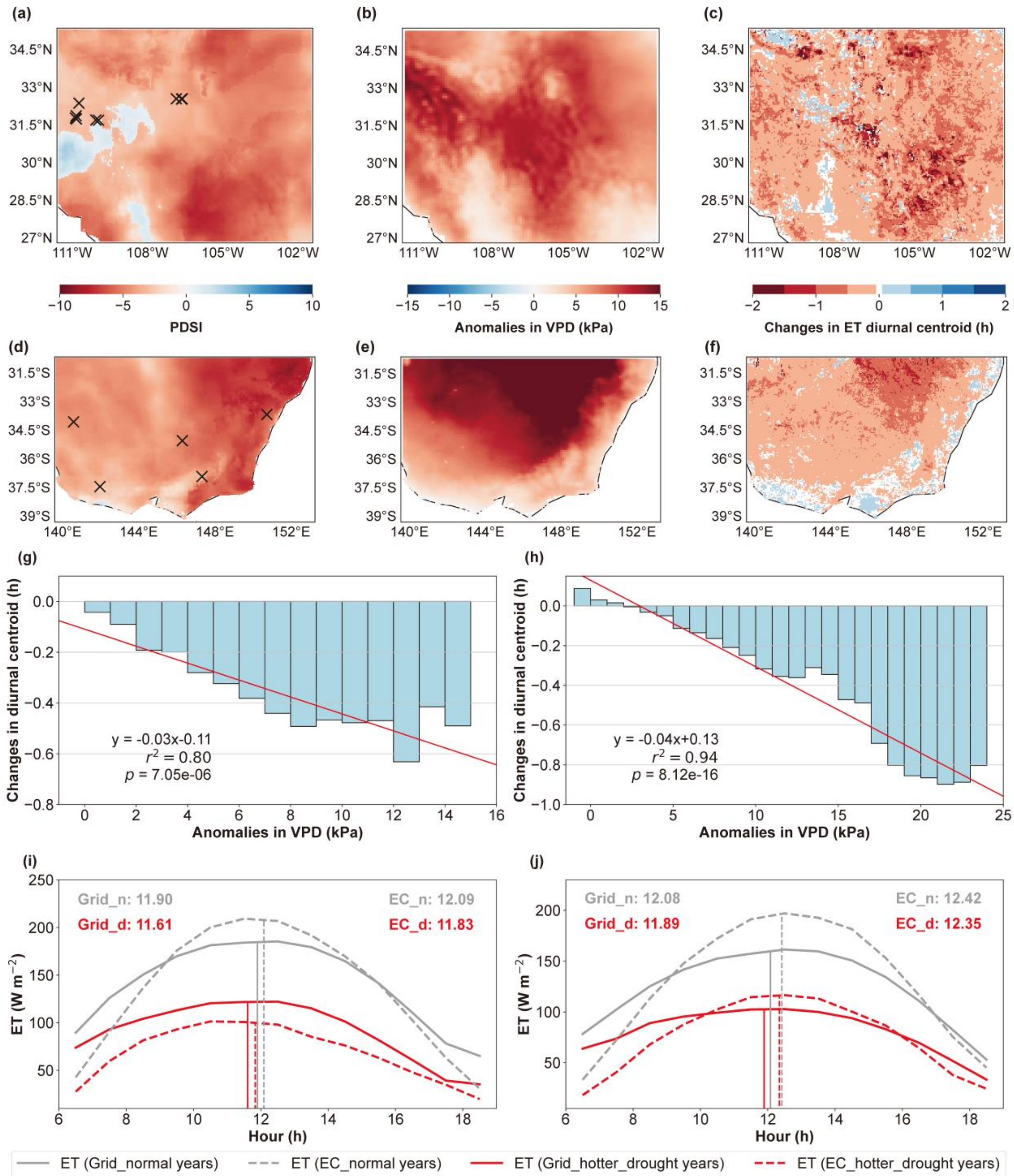

L383: "13PM" is odd; consider 13:00 or 01:00PM; I think 13:00 to be consistent, check all reported timings and report in the same standard/format, a 24h clock.

Response: We thank the reviewer for pointing this out. We agree that "13PM" is a little odd. Because the figures report time using decimal hours, we have revised the manuscript to use decimal time consistently throughout the text and figures. For example, "13PM" has been changed to "13.00 h local solar time," and other reported timings have been checked and standardized accordingly. This format is also consistent with our reporting of canopy evaporation timing, such as 12.52 h local solar time.

L443-445: Great job synthesizing these important mechanisms. In the final sentence of the paragraph, I think reminding the reader that this conclusion is supported by ECHO-ET at its scale of observation. Perhaps a sentence following this one, could indicate that ECHO-ET is sensing the canopy-scale over relatively broad areas, compared to cells, organs, and organisms which all likely vary in their dynamics at those finer spatial scales; nevertheless, the emergence of a globally coherent midday depression in ET really highlights the importance of this passive biophysical consequence of dehydration over signal-response. Interesting!

Response: We appreciate the reviewer's thoughtful suggestion. We agree that our conclusion should more clearly reflect the observational scale of ECHO-ET. We have added a sentence to clarify that ECHO-ET senses canopy-scale ET over relatively broad areas, whereas cellular-, organ-, and organism-level responses may vary at finer spatial scales (Lines 470-474).

Reviewer #3 (Remarks to the Author)

No more comments.

Response: We sincerely thank the reviewer for the positive evaluation of our revised manuscript and for confirming that there are no further comments. We greatly appreciate the reviewer's constructive and insightful feedback throughout the review process, which has helped us improve the clarity, interpretation, and overall quality of the manuscript.
