# Supplementary material for: NASA’s ECOSTRESS satellite reveals widespread midday depression in ecosystem evapotranspiration
Source: Nat Commun. 2026 Aug 25;17:8976. doi: 10.1038/s41467-026-76873-x (PMC13507112; doi:10.1038/s41467-026-76873-x)
Supplement: Supplementary file 3 [file 41467_2026_76873_MOESM3_ESM.pdf]

Description of Additional Supplementary Files

File Name: Supplementary Data 1.xlsx

Description: Description of the eddy covariance flux sites used in this study: Site ID, site name, country, start year and end year of the measurements, latitude (Lat), longitude (Lon), vegetation type (Veg), and climate type (Clim). The hourly and monthly-average hourly statistics (R^2, RMSE, PBIAS) for validating ECHO-ET are also provided.
