# Supplementary material for: NASA’s ECOSTRESS satellite reveals widespread midday depression in ecosystem evapotranspiration
Source: Nat Commun. 2026 Aug 25;17:8976. doi: 10.1038/s41467-026-76873-x (PMC13507112; doi:10.1038/s41467-026-76873-x)
Supplement: Supplementary file 4 [file 41467_2026_76873_MOESM5_ESM.pdf]

nature portfolio

Corresponding author(s): Jingfeng Xiao

Last updated by author(s): Jun 15, 2026

Reporting Summary

Nature Portfolio wishes to improve the reproducibility of the work that we publish. This form provides structure for consistency and transparency in reporting. For further information on Nature Portfolio policies, see our Editorial Policies and the Editorial Policy Checklist.

Statistics

For all statistical analyses, confirm that the following items are present in the figure legend, table legend, main text, or Methods section.

n/a	Confirmed	
☐	☒	The exact sample size (*n*) for each experimental group/condition, given as a discrete number and unit of measurement
☒	☐	A statement on whether measurements were taken from distinct samples or whether the same sample was measured repeatedly
☐	☒	The statistical test(s) used AND whether they are one- or two-sided<br>*Only common tests should be described solely by name; describe more complex techniques in the Methods section.*
☒	☐	A description of all covariates tested
☐	☒	A description of any assumptions or corrections, such as tests of normality and adjustment for multiple comparisons
☐	☒	A full description of the statistical parameters including central tendency (e.g. means) or other basic estimates (e.g. regression coefficient) AND variation (e.g. standard deviation) or associated estimates of uncertainty (e.g. confidence intervals)
☐	☒	For null hypothesis testing, the test statistic (e.g. *F*, *t*, *r*) with confidence intervals, effect sizes, degrees of freedom and *P* value noted<br>*Give P values as exact values whenever suitable.*
☒	☐	For Bayesian analysis, information on the choice of priors and Markov chain Monte Carlo settings
☒	☐	For hierarchical and complex designs, identification of the appropriate level for tests and full reporting of outcomes
☐	☒	Estimates of effect sizes (e.g. Cohen's *d*, Pearson's *r*), indicating how they were calculated

Our web collection on statistics for biologists contains articles on many of the points above.

Software and code

Policy information about availability of computer code

Data collection	All data were programmatically retrieved from public repositories: NASA AppEEARS API for ECOSTRESS ET/LST and cloud masks; Google Earth Engine (JavaScript Code Editor and Python API) for Landsat Collection-2 surface reflectance, MODIS MCD43C4/MCD12C1 and ESA WorldCover 10m v200; the Copernicus Climate Data Store (CDS API) for ERA5-Land; and the AmeriFlux/NEON/ICOS/OzFlux/HiWATER portals for tower observations; Integrated Carbon Observation System: ICOS for FLUXCOM-X. Retrieval and initial QA/QC (QA-bit masks, range checks, time-zone harmonization) were performed with short custom Python scripts. No commercial software was required for data collection.
Data analysis	All analyses were conducted in Python (3.10+) using NumPy v1.26.4 for array-based numerical calculations, raster masking, missing-value handling, and centroid calculations; pandas v2.0.3 for tabular data processing and Excel I/O; xarray v2023.6.0 and rioxarray v0.17.0 for gridded data handling; rasterio v1.3.9 and GDAL v3.6.4 for geospatial I/O, GeoTIFF reading and writing, reprojection, resampling, raster metadata handling, and raster-based post-processing; GeoPandas v0.14.2 and Shapely v2.0.2 for vector data processing, point buffering, and shapefile generation; Requests v2.31.0 for submitting and downloading ECOSTRESS data requests through the AppEEARS API; scikit-learn v1.5.1 for modeling, including train/test splitting, Random Forest modeling, nested cross-validation with fixed random seeds, and model evaluation; SHAP v0.50.0 for attribution analysis; joblib v1.4.2 for saving and loading trained models; and SciPy v1.17.0 for statistical analyses, including curve fitting, linear regression, Pearson correlation, and Kolmogorov–Smirnov tests. Figures were produced using Matplotlib v3.7.2 for plotting, figure layout, colormap design, annotations, and PDF/EPS figure export; Salem v0.3.11 for geospatial visualization support; Cartopy v0.22.0 for map projections, geographic gridlines, and Natural Earth basemap features; and Seaborn v0.12.2 for kernel density plots. ArcPy was additionally used for ArcGIS-based raster–vector operations, including zonal statistics, feature generation, and extraction of raster values to points.

For manuscripts utilizing custom algorithms or software that are central to the research but not yet described in published literature, software must be made available to editors and reviewers. We strongly encourage code deposition in a community repository (e.g. GitHub). See the Nature Portfolio guidelines for submitting code & software for further information.

Data

Policy information about availability of data

All manuscripts must include a data availability statement. This statement should provide the following information, where applicable:

- Accession codes, unique identifiers, or web links for publicly available datasets
- A description of any restrictions on data availability
- For clinical datasets or third party data, please ensure that the statement adheres to our policy

ECOSTRESS-ET（https://appeears.earthdatacloud.nasa.gov/）
ECOSTRESS-LST（https://appeears.earthdatacloud.nasa.gov/）
ECOSTRESS cloud mask（https://appeears.earthdatacloud.nasa.gov/）
ERA5-Land reanalysis（https://cds.climate.copernicus.eu/cdsapp#!/home）
AmeriFlux（https://ameriflux.lbl.gov/）
ICOS（https://www.icos-cp.eu/）
OzFlux（https://www.ozflux.org.au/）
NEON（https://ameriflux.lbl.gov/）
HiWATER（https://data.tpdc.ac.cn/）
hPET（https://doi.org/10.5523/bris.q8bujazzda0s2aykkvo0q0ctp）
MODIS MCD43C4（https://search.earthdata.nasa.gov/）
MODIS MCD12C1（https://search.earthdata.nasa.gov/）
Landsat Collection 2 surface reflectance（https://developers.google.com/earth-engine/datasets/catalog/LANDSAT_LC08_C02_T1_L2）
Köppen-Geiger climate classification（https://hess.copernicus.org/articles/11/1633/2007/）
Palmer Drought Severity Index（PDSI, https://climate.northwestknowledge.net/TERRACLIMATE/index_directDownloads.php）
Standardized Precipitation Evapotranspiration Index（SPEI, https://zenodo.org/records/8060268）
FLUXCOM-X (https://www.icos-cp.eu/data-products/fluxcom-x-global-fluxes-collection)
ESA WorldCover 10m v200 (https://developers.google.com/earth-engine/datasets/catalog/ESA_WorldCover_v200)

Research involving human participants, their data, or biological material

Policy information about studies with human participants or human data. See also policy information about sex, gender (identity/presentation), and sexual orientation and race, ethnicity and racism.

Reporting on sex and gender	Not applicable. This study did not involve human participants or human data.
Reporting on race, ethnicity, or other socially relevant groupings	Not applicable.
Population characteristics	Not applicable.
Recruitment	Not applicable.
Ethics oversight	Not applicable.

Note that full information on the approval of the study protocol must also be provided in the manuscript.

Field-specific reporting

Please select the one below that is the best fit for your research. If you are not sure, read the appropriate sections before making your selection.

☐ Life sciences ☐ Behavioural & social sciences ☒ Ecological, evolutionary & environmental sciences

For a reference copy of the document with all sections, see nature.com/documents/nr-reporting-summary-flat.pdf

Ecological, evolutionary & environmental sciences study design

All studies must disclose on these points even when the disclosure is negative.

Study description	Objective: To quantify quantified the widespread midday depression in evapotranspiration (ET) globally<br>Design: Synthesis combining ET measurements from the ECOsystem Spaceborne Thermal Radiometer Experiment on Space Station (ECOSTRESS) and machine learning.<br>Units & replication: Hourly ET dataset (unit: W m-2) from 2018 to 2022 using ECOSTRESS ET snapshots.<br>Response variables: ECOSTRESS ET, predictors include meteorology and vegetation indices, and flux-tower records.

Research sample	The 1385888 samples of 0.05 deg comprised from instantaneous ET snapshots across the globe were collected from ECOSTRESS L3_ET_PT-JPL product (ECO3ETPTJPL.001). We used MODIS and ERA5-Land for vegetation index/meteorology and flux-tower records for ET correction/validation.
Sampling strategy	We selected 100 distinct 1° × 1° blocks for downloading ECOSTRESS ET snapshots. This selection was based on the proportional representation of various land cover types present within the ECOSTRESS coverage area.
Data collection	ECOSTRESS ET (70 m) was filtered for cloud and view geometry. For the flux data, those years with consecutive high-quality observations during the growing season were selected for analysis. In addition, only days with complete observations throughout the day were used.
Timing and spatial scale	We developed a global gridded, hourly ET dataset from 2018 to 2022 at 0.05 deg.
Data exclusions	The ECOSTRESS ET product (ECO3ETPTJPL.001) processed only high-quality raster data, requiring precise geolocation (<50 m) and clear-sky conditions, to minimize the impact of geolocation issues and cloud contamination on the imagery.
Reproducibility	Codes and workflow are available at https://zenodo.org/records/20790910
Randomization	To ensure the model's accuracy across different land cover types, we allocated 70% of the dataset from each land cover type, including numerous grid cells with ET and all explanatory variable data, for training, while reserving the remaining 30% for validation purposes.
Blinding	Blinding during data acquisition was not feasible because all inputs were collected automatically from public satellite products and eddy-covariance networks. Model performance was reported only on a geographically/temporally held-out test set that remained unseen until final evaluation. All analyses were executed by scripted pipelines with fixed random seeds and change logs.

Did the study involve field work? ☐ Yes ☒ No

Reporting for specific materials, systems and methods

We require information from authors about some types of materials, experimental systems and methods used in many studies. Here, indicate whether each material, system or method listed is relevant to your study. If you are not sure if a list item applies to your research, read the appropriate section before selecting a response.

Materials & experimental systems

n/a	Involved in the study
☒	☐ Antibodies
☒	☐ Eukaryotic cell lines
☒	☐ Palaeontology and archaeology
☒	☐ Animals and other organisms
☒	☐ Clinical data
☒	☐ Dual use research of concern
☒	☐ Plants

Methods

n/a	Involved in the study
☒	☐ ChIP-seq
☒	☐ Flow cytometry
☒	☐ MRI-based neuroimaging

Plants

Seed stocks	Not applicable. This study did not involve plants.
Novel plant genotypes	Not applicable. This study did not involve plants.
Authentication	Not applicable. This study did not involve plants.
